# Upregulation of lipid metabolism genes in the breast prior to cancer diagnosis

**DOI:** 10.1038/s41523-020-00191-8

**Published:** 2020-10-06

**Authors:** Natascia Marino, Rana German, Xi Rao, Ed Simpson, Sheng Liu, Jun Wan, Yunlong Liu, George Sandusky, Max Jacobsen, Miranda Stovall, Sha Cao, Anna Maria V. Storniolo

**Affiliations:** 1 Susan G. Komen Tissue Bank at the IU Simon Cancer Center, Indianapolis, IN 46202 USA; 2 Department of Medicine, Indiana University School of Medicine, Indianapolis, IN 46202 USA; 3 Department of Medical and Molecular Genetics, Indiana University School of Medicine, Indianapolis, IN 46202 USA; 4 Pathology and Laboratory Medicine, Indiana University School of Medicine, Indianapolis, IN 46202 USA; 5 Department of Biostatistics, Indiana University School of Medicine, Indianapolis, IN 46202 USA

**Keywords:** Breast cancer, Cancer epidemiology, Risk factors, Oncogenesis

## Abstract

Histologically normal tissue adjacent to the tumor can provide insight of the microenvironmental alterations surrounding the cancerous lesion and affecting the progression of the disease. However, little is known about the molecular changes governing cancer initiation in cancer-free breast tissue. Here, we employed laser microdissection and whole-transcriptome profiling of the breast epithelium prior to and post tumor diagnosis to identify the earliest alterations in breast carcinogenesis. Furthermore, a comprehensive analysis of the three tissue compartments (microdissected epithelium, stroma, and adipose tissue) was performed on the breast donated by either healthy subjects or women prior to the clinical manifestation of cancer (labeled “susceptible normal tissue”). Although both susceptible and healthy breast tissues appeared histologically normal, the susceptible breast epithelium displayed a significant upregulation of genes involved in fatty acid uptake/transport (CD36 and AQP7), lipolysis (LIPE), and lipid peroxidation (AKR1C1). Upregulation of lipid metabolism- and fatty acid transport-related genes was observed also in the microdissected susceptible stromal and adipose tissue compartments, respectively, when compared with the matched healthy controls. Moreover, inter-compartmental co-expression analysis showed increased epithelium-adipose tissue crosstalk in the susceptible breasts as compared with healthy controls. Interestingly, reductions in natural killer (NK)-related gene signature and CD45+/CD20+ cell staining were also observed in the stromal compartment of susceptible breasts. Our study yields new insights into the cancer initiation process in the breast. The data suggest that in the early phase of cancer development, metabolic activation of the breast, together with increased epithelium-adipose tissue crosstalk may create a favorable environment for final cell transformation, proliferation, and survival.

## Introduction

Despite improved screening leading to a decline in its incidence rate, breast cancer still poses a substantial public health burden. About 279,100 new breast cancer cases and 42,690 breast cancer deaths are estimated to occur in 2020 in the United States^1^. While there has been a dramatic improvement in disease detection and treatment, our understanding of the factors involved in tumor initiation and our ability to selectively interfere with this process are very limited. It has been reported that the histologically normal but genetically altered tissue surrounding the tumor (also known as “normal adjacent to the tumor”, NAT) promotes mechanisms for increased replicative capacity, genomic instability, and, therefore, provides a microenvironment that supports cancer progression^2^. Whether the tumor affects the NAT or vice versa remains still unclear, especially in the context of the earliest phases of cancer development. Pre-malignant cells are exposed to a myriad of altered forces and signals from the surrounding microenvironment, including diffusible growth factors, inflammatory cytokines, free fatty acids, and matrix-remodeling enzymes. These paracrine and autocrine factors can mediate intercellular crosstalk and dramatically modify the cells’ behavior and therefore tumor development and progression^3–5^. Understanding the pre-malignant field is critical for both elucidating the impact of the microenvironmental changes on cancer initiation and for the development of biomarkers for breast cancer risk assessment.

Until recently, the research community was limited to studying pre-malignancy in mouse models and immortalized cell lines^6^. Breast tissue obtained from either women undergoing reduction mammoplasty or NAT have been used as poor substitutes for healthy controls. However, their normalcy has been questioned as they may be affected by hyperproliferative conditions and likely harbor genetic and/or epigenetic aberrations^7,8^.

The establishment of the Susan G. Komen Tissue Bank at the IU Simon Cancer Center (KTB), the only repository of truly normal breast tissue, offers the possibility to address this limitation. In the thirteen years since its foundation, the KTB has collected breast tissue core biopsies from more than 5500 donors^9^. The well-annotated human breast tissue specimens from the KTB represent an excellent system in which to investigate both the physiology of the normal breast^7,10^ and breast cancer development^8,11,12^.

As expected^1^, a relatively small number of KTB donors (≈5%) diagnosed with breast cancer a few years post-donation. The specimens originally donated by these women (here labeled “susceptible normal tissue”) provide a window into the earliest phases of breast cancer development. Hence, to acquire new insights into the breast cancer initiation process, we examined how cancer-prone breast tissue differs from the contralateral breast and, especially, from matched healthy breast tissue.

In this study, we investigated the transcriptomic profile of the breast epithelium prior to and post tumor diagnosis, and the differences between susceptible normal and matched healthy breast tissues. In the latter, for a more comprehensive analysis, microdissection of the three tissue compartments (epithelium, stroma, and adipose tissue) was performed. Our data suggest that a metabolic rewiring, displayed as an increased expression of genes involved in lipid metabolism and adipogenesis, is one of the first features of the breast affected by tumorigenesis. This metabolic activation is also reflected by an increased co-expression network interaction between the epithelial compartment and the surrounding adipose tissue. Furthermore, we investigated immune cell infiltration by examining both immune cell-related signatures and specific immunostaining in the breast tissues. The susceptible breast tissue showed a reduction in resting natural killer (NK) and B cells, suggesting that an immunosuppressive phenotype may promote a pro-tumorigenic environment.

## Results

### Comparison of the breast epithelium transcriptome prior to and post-cancer diagnosis

**Fig. 1:**
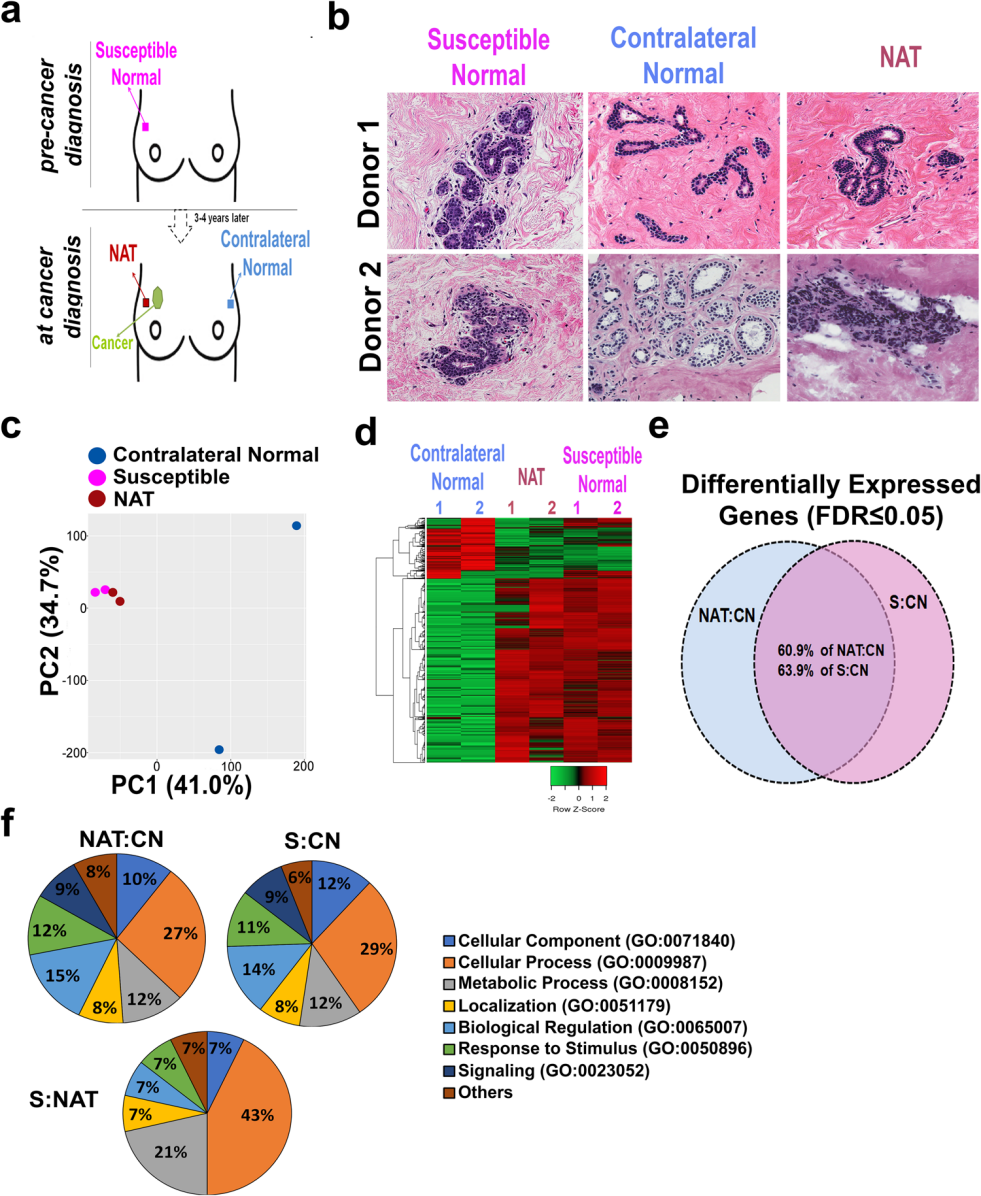
Transcriptomic analysis of paired contralateral normal, normal adjacent to the tumor (NAT), and susceptible microdissected breast epithelial samples. **a** Descriptive design of the collection of susceptible, NAT, and contralateral breast tissue cores. Breast tissue cores were collected from the upper-outer quadrant. **b** Hematoxylin and eosin staining of tissue sections from contralateral normal, susceptible, and NAT breast specimens. Images at ×40 magnification are shown. **c** Principal component analysis of the transcriptomic profiles from contralateral normal (blue), adjacent normal (purple), and susceptible (pink) breast epithelium showed a clear separation of the NAT and susceptible from the contralateral samples. **d** Hierarchical clustering heatmap of the differentiated transcripts between susceptible, adjacent normal, and contralateral breast tissue showed similarity between NAT and susceptible with both being highly distinct from the contralateral breast. **e** Venn diagram of differentially expressed genes (DEGs) in NAT vs. contralateral normal (CN) (NAT:CN) and susceptible (S) vs. CN (S:CN) showed that the two comparisons share 60.9% of DEGs in NAT:CN and 63.9% of the DEGs between S and CN. **f** Gene ontology enrichment analysis of differentially expressed genes between NAT and CN, S and CN, and S and contralateral normal, and susceptible and NAT breast epithelium was performed using PANTHER. Pie charts showing the percentage of the significantly enriched biological process categories against the total.

To investigate the molecular changes of breast tissue prior to and post-cancer diagnosis, we compared the transcriptomic profiles of the microdissected epithelium of the breast tissue cores from two women who donated tissue biopsies from both the affected breast (prior and post diagnosis of cancer, here labeled susceptible and NAT, respectively) and the contralateral healthy breast (Fig. 1a and Table 1). In terms of clinicopathological features of the tumors, one is estrogen receptor (ER) positive with both ductal carcinoma in situ (DCIS) and invasive ductal carcinoma (IBC); the other is ER-positive DCIS. Histological analysis of the hematoxylin and eosin-stained sections revealed a normal phenotype for both the susceptible and contralateral breast tissues, while the NAT sections displayed minor hyperplasia (Fig. 1b). Upon transcriptome analysis of the microdissected breast epithelium, we detected 262 transcripts differentially expressed between NAT and contralateral normal samples, and, among those, 156 genes (60.9%) were differentially expressed also between susceptible normal and contralateral normal epithelium (FDR < 0.05; Fig. 1c–e). NAT and susceptible samples showed high similarity in their transcriptome profiles, with only 16 transcripts differentially expressed (*P* < 0.01) (Supplementary Table 1). Gene Ontology (GO) analysis showed enrichment in genes involved in cellular processes, biological regulation, and metabolic processes in the NAT (27, 15, and 12%, respectively) and in the susceptible normal (29, 14, and 12%, respectively) breast epithelial compartments as compared with the paired contralateral breast (Fig. 1f). Furthermore, the genes differentially expressed between NAT and susceptible were also linked with cellular processes (43%), including cell cycle and cellular metabolism, and with the metabolic processes (21%).

**Table 1:** Demographics of the women who donated breast tissue cores prior to and post-cancer diagnosis.

	Prior to cancer diagnosis			At cancer diagnosis			Tumor characteristics	
	Age	Menopausal status	BMI	Age	Menopausal status	BMI	Type	ER/PR/Her2^a^
Donor 1	49	Pre	24.2	52	Pre	23.6	DCIS	+/−/−
Donor 2	52	Pre	24.4	55	Pre	24.2	DCIS, IBC	+/+/−

*BMI* body mass index, *DCIS* ductal carcinoma in situ, *IBC* invasive breast carcinoma.

^a^Positivity to estrogen receptor/progesterone receptor/Her2 amplification.

### Transcriptome profiling of the susceptible and healthy breast

Both susceptible breast epithelium and NAT displayed molecular differences as compared with the paired contralateral normal, suggesting an early activation of specific pathways (involved in cellular and metabolic processes) even prior to cancer diagnosis.

**Fig. 2:**
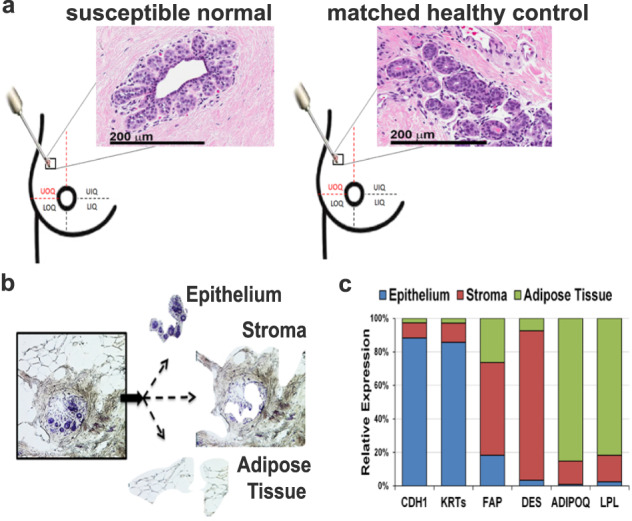
Microdissection of breast tissue compartments. **a** The breast tissue biopsies from women either susceptible to cancer (Susc) or matched healthy controls (HC) were collected from the upper-outer quadrant of the breast (either right or left). Hematoxylin and eosin staining of susceptible and healthy breast tissues showed the lack of any detectable histological breast abnormality at the time of donation. Images at ×40 magnification are shown. Scale bar: 200 µm. **b** Laser microdissection microscopy was used to isolate the epithelial, stroma, and adipose tissue compartments separately from each of the 23 fresh-frozen biopsies. **c** Expression of markers specific for the epithelium (E-Cadherin, CDH1, and pan-Keratins (KRTs)), stroma (fibroblast activation protein, FAP, and desmin, DES), adipose tissue (adiponectin, ADIPOQ, and lipoprotein lipase, LPL) was obtained from the transcriptome profiling. Each marker was highly expressed in the specific microdissected tissue compartment as compared with the other areas of the breast (*P* < 0.0001), thus indicating sample purity.

To further investigate the molecular features of the early phase of breast cancer development as represented in the breast tissue prior to cancer diagnosis, we employed transcriptome sequencing and defined the genomic differences between histologically normal breast tissue cores donated by susceptible and those from matched healthy women (Fig. 2a). Because the subjects included in this study reported different types of breast cancer (DCIS, IDC, and invasive lobular carcinoma), the limited sample size only allowed us to investigate pan-breast pre-cancer distinctions from the normal breast. Nevertheless, to comprehensively capture the transcriptomic alterations of the breast, we microdissected and profiled separately the three breast tissue compartments (epithelium, stroma, and adipose tissue) from 7 susceptible women and 16 matched healthy controls (Fig. 2b, c, Table 2, and Supplementary Fig. 1). The two cohorts included Caucasian premenopausal women with mean age of 45.7 years and mean body mass index of 27.

**Table 2:** Demographics of the study cohorts: susceptible or matched healthy controls (HC).

	Age at Donation	BMI	Breast cancer history	Collection year	Year of diagnosis	Breast cancer type	ER	PR	HER2
Susceptible 1	48	22	No	2010	2011	ILC	+	+	−
Susceptible 2	46	22	No	2007	2008	ILC	−	−	−
Susceptible 3	49	25	No	2011	2015	IDC	+	−	−
Susceptible 4	43	33	No	2011	2012	DCIS	+	−	−
Susceptible 5	50	35	No	2012	2014	DCIS	+	+	−
Susceptible 6	52	24	No	2012	2015	IDC	+	+	−
Susceptible 7	37	34	No	2009	2013	DCIS	N/A	N/A	N/A
HC1a	46	24	No	2010					
HC1b	49	23	No	2012					
HC2a	48	20	No	2009					
HC2b	47	26	No	2012					
HC2c	48	26	No	2014					
HC3a	46	26	No	2011					
HC3b	45	22	No	2012					
HC3c	47	24	No	2011					
HC4a	42	39	No	2013					
HC4b	45	28	No	2012					
HC5a	48	31	No	2008					
HC5b	48	32	No	2013					
HC6a	49	29	No	2013					
HC6b	50	24	No	2013					
HC7a	35	28	No	2011					
HC7b	34	27	No	2012					

*HC* healthy control, *BMI* body mass index, *N/A* not available, *DCIS* ductal carcinoma in situ, *IDC* invasive ductal carcinoma, *ILC* infiltrating lobular carcinoma, *ER* estrogen receptor, *PR* progesterone receptor.

Breast tissues donated by premenopausal, Caucasian women were analyzed through laser microdissection and transcriptomic profiling.

Unsupervised principal components analysis of the transcriptome profiles showed that the menstrual phase of the subjects (follicular and luteal) contributes to a large variation in our dataset (Supplementary Fig. 2). The breast tissue is an organ physiologically affected by cyclic variations of circulating hormones, which may influence cellular processes and gene expression not only in the epithelium compartment but also in stroma^13^ and adipose tissue^14^. To eliminate this confounding factor, we removed from each breast compartment dataset the transcripts whose expression significantly (*P* < 0.05) varies between follicular and luteal phase within the healthy breast cohort (Supplementary Table 3). Furthermore, previously reported menstrual phase-dependent gene expression variations were also excluded from the epithelium dataset^10^.

**Fig. 3:**
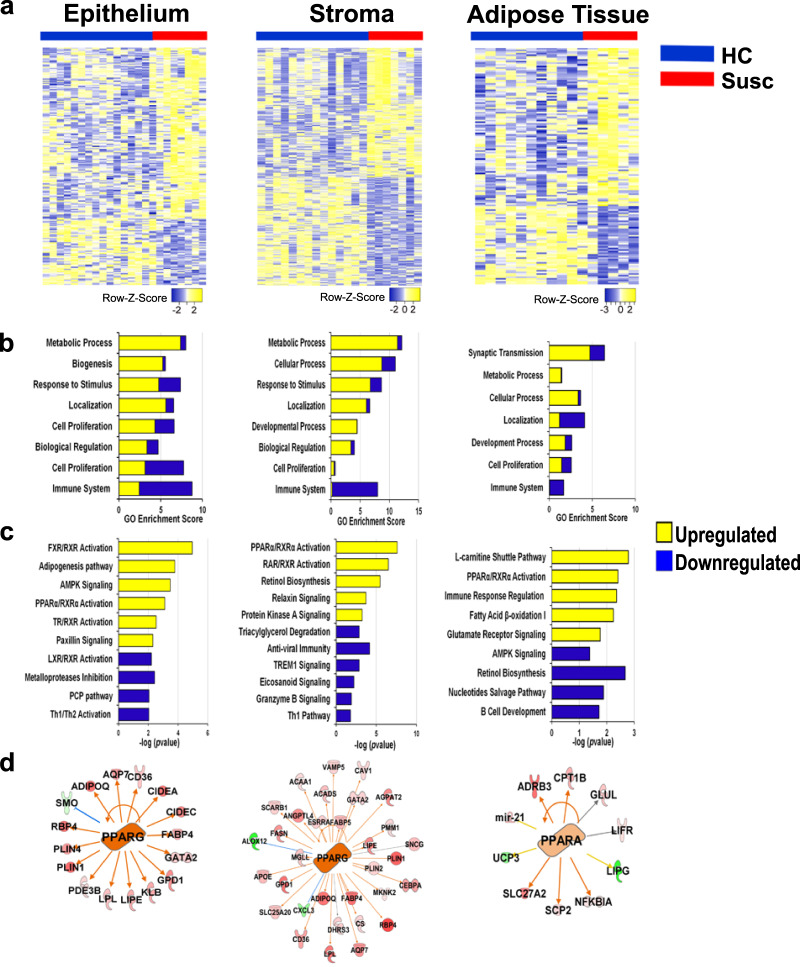
Transcriptomic profiling of the microdissected breast compartments. **a** Hierarchical clustering heatmaps of the differentiated transcripts in the microdissected breast epithelium, stroma, and adipose tissue between susceptible (red line) and healthy control (blue line). The epithelium and adipose tissue show a higher number of upregulated genes as compared with the stromal compartment. **b** Gene ontology enrichment (PANTHER_GO) and **c** pathway analyses (IPA v01-06) of upregulated and downregulated genes. **d** Master regulator analysis (IPA v01-06) for the differentially expressed genes between susceptible and healthy control breasts in the three breast tissue compartments. Upregulated molecules in the susceptible as compared with the healthy controls are in red, and the downregulated molecules are in green. Arrows indicate the intermolecular relationship: activation (orange), inhibition (blue), effect not predicted (gray), inconsistency with the state of the downstream molecule (yellow).

We compared the transcriptome profiling of each breast tissue compartment between the susceptible normal and matched healthy breasts. We detected 222 transcripts differentially expressed between the two cohorts in the microdissected breast epithelium, 484 in the microdissected breast stroma, and 148 in the microdissected breast adipose tissue (*P* < 0.05) (Fig. 3a and Supplementary Tables 4–6). The ten most upregulated and downregulated transcripts in each compartment in the susceptible breast as compared with healthy breast are shown in Table 3.

**Table 3:** Top 10 upregulated and 10 downregulated genes in susceptible versus healthy control breast.

Gene Symbol	Gene Name	FC^a^,^b^	*p* value^b^	Gene Symbol	Gene Name	FC	*p* value
	**Epithelium_Upregulated**				**Epithelium_Downregulated**		
AQP7	aquaporin 7	2.5	1.2E-05	ZFP57	ZFP57 zinc finger protein	−29.0	3.8E-04
AKR1C1	aldo-keto reductase family 1 C1	4.3	1.2E-05	SNORA13	small nucleolar RNA, H/ACA box 13	−4.3	6.1E-03
XIRP2	xin actin binding repeat containing 2	7.4	2.4E-05	SAA4	serum amyloid A4, constitutive	−2.5	6.9E-03
SIM1	single-minded transcription factor 1	5.4	6.2E-05	MMP27	matrix metallopeptidase 27	−2.9	8.1E-03
RBP4	retinol binding protein 4	3.7	2.8E-04	FN3KRP	fructosamine 3 kinase related protein	−1.4	8.3E-03
PLIN1	perilipin 1	3.9	3.1E-04	TMEM229B	transmembrane protein 229B	−1.7	8.4E-03
SORCS1	sortilin related VPS10 receptor 1	3.0	3.7E-04	TSPAN11	tetraspanin 11	−1.7	8.6E-03
LIPE	lipase E, hormone sensitive type	2.2	4.4E-04	EMX2	empty spiracles homeobox 2	−2.1	1.0E-02
ADIPOQ	adiponectin	3.8	1.9E-03	CXCR6	C-X-C motif chemokine receptor 6	−1.7	1.1E-02
CD36	CD36 molecule	1.7	4.3E-03	LILRA1	leukocyte Ig like receptorA1	−1.9	1.1E-02
	**Stroma_Upregulated**				**Stroma_Downregulated**		
MAPK10	mitogen-activated protein kinase 10	1.6	4.1E-04	ANKRD36BP2	ankyrin repeat domain 36B pseudogene2	−2.1	2.1E-05
DDIT4	DNA damage inducible transcript 4	1.7	7.8E-04	LRGUK	leucine repeats and guanylate kinase	−2.3	1.2E-04
TCAP	titin-cap	2.6	8.6E-04	GAS2	growth arrest specific 2	−2.4	1.6E-04
ALDH1L1	aldehyde dehydrogenase 1 L1	2.7	1.0E-03	WNT10A	Wnt family member 10A	−2.1	1.7E-04
TNNC2	troponin C2, fast skeletal type	2.9	1.1E-03	DERL3	derlin 3	−2.1	2.9E-04
SLC7A10	solute carrier family 7 member 10	3.3	1.2E-03	PIM2	Pim-2 proto-oncogene	−2.0	3.2E-04
FAH	fumarylacetoacetate hydrolase	1.8	1.3E-03	GPR15	G protein-coupled receptor 15	−6.6	3.4E-04
TNNT1	troponin T1, slow skeletal type	4.8	2.0E-03	CD38	CD38 molecule	−1.8	4.8E-04
HK2	hexokinase 2	2.0	2.7E-03	TNFRSF18	TNF receptor superfamily member 18	−3.4	1.2E-03
NAT8L	N-acetyltransferase 8 like	2.5	5.4E-03	NLRP11	NLR family pyrin domain containing 11	−6.9	1.2E-03
	**Adipose Tissue_Upregulated**				**Adipose Tissue_Downregulated**		
IDH2	isocitrate dehydrogenase 2	1.7	8.3E-04	CPA4	carboxypeptidase A4	−6.2	5.8E-04
RIMS2	regulating synaptic exocytosis2	9.5	8.4E-04	FCHO1	FCH domain only 1	−2.7	5.8E-04
SPTB	spectrin beta, erythrocytic	2.5	8.8E-04	CES4A	carboxylesterase 4A	−2.1	8.3E-04
NTS	neurotensin	7.3	1.6E-03	CAMK2B	calcium/calmodulin dependent kinase II β	−4.9	1.0E-03
BRIP1	BRCA1 interacting protein 1	2.1	2.1E-03	ASGR1	asialoglycoprotein receptor 1	−4.2	2.7E-03
ETV2	ETS variant 2	3.6	2.1E-03	ZDHHC11	zinc finger DHHC-type containing 11	−2.1	2.8E-03
SLC6A13	solute carrier family 6 member 13	3.7	3.6E-03	APOBEC3G	apolipoprotein B mRNA editing 3G	−1.7	3.5E-03
FKBP5	FK506 binding protein 5	1.9	4.4E-03	UCP3	uncoupling protein 3	−2.5	3.9E-03
RTN1	reticulon 1	1.7	5.0E-03	DPYSL4	dihydropyrimidinase like 4	−2.8	4.1E-03
FABP4	fatty acid binding protein 4	2.3	4.7E-03	BLNK	B-cell linker	−1.8	7.9E-03

^a^Fold Change.

^b^obtained from EdgeR using negative binomial model-based method.

GO enrichment, pathway, and network analyses of the differentially expressed genes in the susceptible breast epithelium as compared with the matched healthy controls clustered the majority of the upregulated genes in the metabolic processes including adipogenesis (*P* = 1.62E-04), lipid metabolism with FXR/RXR activation (*P* = 1.13E-05) and PPARα/RXRα activation (*P* = 7.8E-04), biogenesis, and AMPK signaling activation (*P* = 3.4E-04) (Fig. 3b, c and Supplementary Tables 7 and 8). However, downregulated genes were mostly linked with immune system signaling. Similarly, genes upregulated in the susceptible stroma were involved mostly in metabolic pathways (*P* = 2.5E-08 – *P* = 4.6E-06; i.e., RAR/RXR activation, adipogenesis, PPARα/RXRα activation, retinol biosynthesis, triacylglycerol degradation) while the downregulated genes were linked with immune system signaling (*P* = 1.4E-03 – *P* = 1.6E-02; i.e., antiviral immunity, TREM1 signaling, and Th1 pathway) (Fig. 3b, c and Supplementary Tables 9 and 10). In both tissue compartments, the cell proliferation pathways included equally upregulated and downregulated genes. Pathway analysis of the genes upregulated in susceptible breast adipose tissue showed the involvement of synaptic transmission, or transmembrane transport, and metabolic processes, including PPARα/RARα activation (*P* = 1.6E-03) and fatty acid oxidation (*P* = 5.7E-03) (Fig. 3b, c and Supplementary Tables 11 and 12).

Upon upstream regulator analysis, PPARγ, a lipid-activated transcription factor regulating lipid uptake, accumulation, and storage^15^, appeared upstream of 16 differentially expressed genes in the epithelium compartment dataset (*P* = 2.3E-08, activation *z* score: 3.8) (Fig. 3d). Both PPARɑ and PPARγ are key upstream regulators of gene expression changes in the susceptible breast stroma (*P* = 1.4E-9, activation *z* score: 4.3 and *P* = 8.3E-11, activation *z* score: 4.7, respectively) (Fig. 3d and Supplementary Fig. 3). PPARɑ is also found upstream of the gene expression changes observed in the susceptible breast adipose tissue (*P* = 1.6E-04, activation *z* score: 0.6) (Fig. 3d).

### Lipid metabolism genes are upregulated in the susceptible breast epithelium

As shown in Table 4, several genes upregulated in the susceptible epithelium are involved in the regulation of lipid metabolism. Among those, we identified genes regulating lipid catabolism. This process can be mediated by either neutral lipolysis or lipophagy^16^. To investigate this biological process, immunohistochemistry experiments were performed on breast tissue sections from an additional 62 pre- and postmenopausal women (mean age: 54; mean body mass index: 29). The lack of difference in the immunohistochemical (IHC) staining of both the autophagy-related proteins, LC3B^17^ and the mitochondrial marker, TOMM20 (to assess specifically mitophagy^18^) between susceptible and healthy breasts suggests activation of neutral lipolysis in the breast epithelium prior to cancer diagnosis (Supplementary Fig. 4).

**Table 4:** List of lipid metabolism-related genes upregulated in the microdissected susceptible breast epithelium.

Gene symbol	Gene name	FC	*P* value
*Lipolysis*			
PLIN1	Perilipin 1	3.9	3.1E-04
LIPE	Lipase E, hormone-sensitive type	2.2	4.4E-04
PLIN4	Perilipin 4	2.5	2.7E-03
LPL	Lipoprotein lipase	1.8	1.6E-02
*Transport/uptake*			
AQP7	Aquaporin 7	2.5	1.2E-05
RBP4	Retinol-binding protein 4	3.7	3.0E-04
CD36	CD36 molecule	1.7	4.3E-03
FABP4	Fatty acid-binding protein 4	2.2	5.5E-03
APOB	Apolipoprotein B	3.0	9.7E-03
*Synthesis*			
AKR1C1	Aldo–keto reductase 1 C1	4.3	1.2E-05
ACSM1	Acyl-CoA synthetase medium-chain 1	13.6	1.7E-05
GPD1	Glycerol-3-phosphate dehydrogenase 1	2.9	3.2E-03
SDS	Serine dehydratase	2.4	5.8E-03
PCCA	Propionyl-CoA carboxylase alpha subunit	1.3	9.0E-03
AKR1C3	Aldo–keto reductase 1 C3	1.7	1.3E-02
ELOVL7	ELOVL fatty acid elongase 7	2.6	1.3E-02
DHRS12	Dehydrogenase/reductase 12	1.3	2.2E-02
ELOVL5	ELOVL fatty acid elongase 5	1.8	2.5E-02
ACSS3	Acyl-CoA synthetase short-chain 3	1.5	3.2E-02
ACACB	Acetyl-CoA carboxylase beta	1.3	3.7E-02
PPARG	Peroxisome proliferator-activated receptor γ	1.4	4.9E-02
FASN	Fatty acid synthase	2.0	5.0E-02
SCD	Stearoyl-CoA desaturase	2.0	7.2E-02
*Lipid metabolism regulation*			
ADIPOQ	Adiponectin, C1Q, and collagen domain containing	3.8	1.9E-03
LEP	Leptin	3.8	1.3E-02

*FC* fold change.

**Fig. 4:**
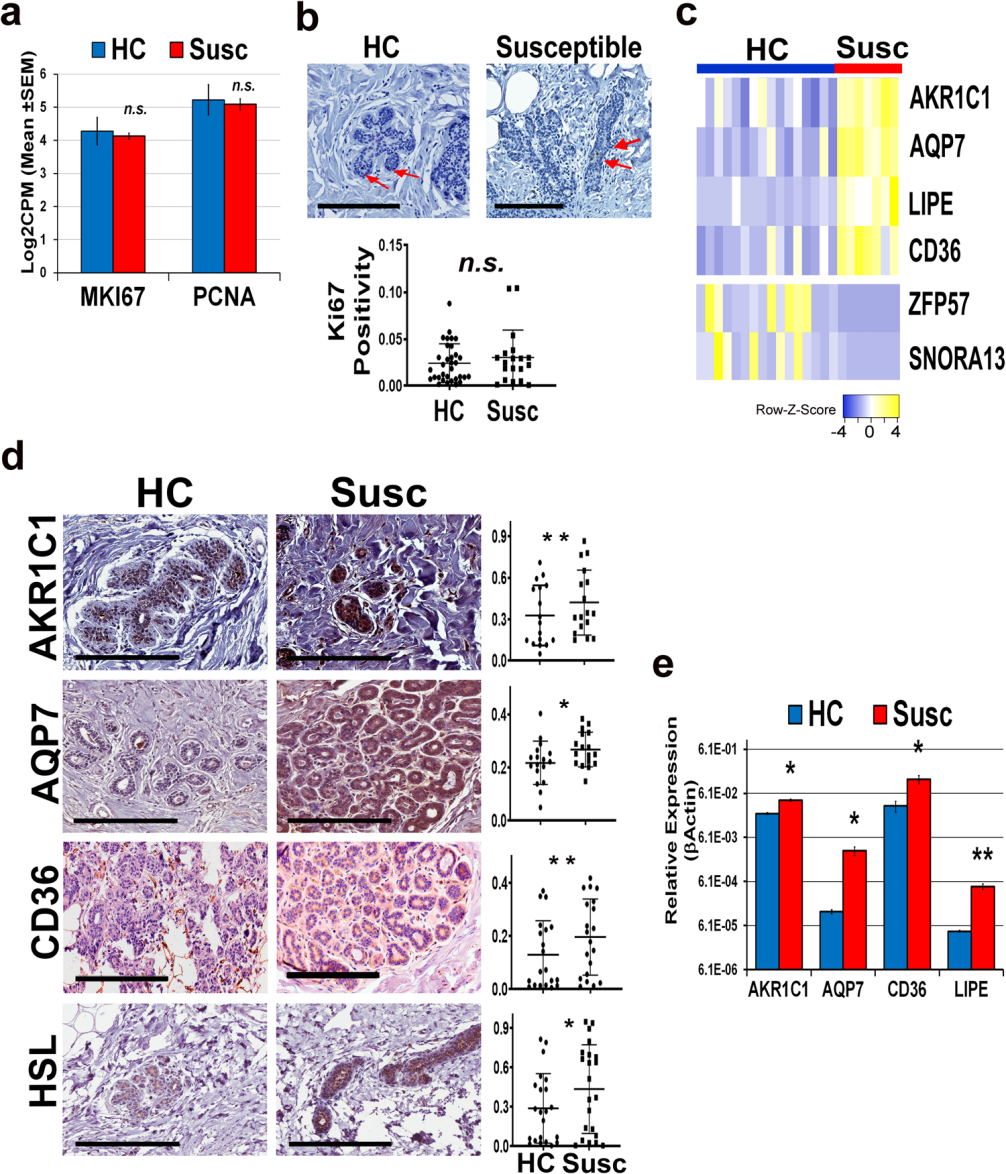
Upregulation of lipid metabolism-related genes in susceptible breast epithelium. **a** mRNA expression (log2CPM) of proliferation markers, MKI67 and PCNA, in susceptible (Susc) and matched healthy (HC) breast epithelium. **b** Immunohistochemical (IHC) analysis of Ki67 in susceptible and healthy control breast tissue. Representative images at ×40 magnification (scale bar: 200 µm) on top. Quantification of the staining (on the bottom) was performed using Aperio Image Scope v12.3.2 and is expressed as positivity or number of positive (brown) versus the total number of cells (hematoxylin stained). Each subject is represented by a dot. **c** Heatmap of six genes highly differentially expressed between susceptible and healthy control samples. **d** IHC staining of susceptible and matched healthy controls (HC) breast tissue sections with antibodies specific for human AKR1C1, AQP7, CD36, and HSL. Images at ×40 magnification are shown. Scale bar: 200 µm. The quantification of each staining is shown on the right. Wilcoxon nonparametric test is used to calculate *P* value. **e** qPCR analysis of lipid metabolism-related genes AKR1C1, AQP7, CD36, and LIPE in primary epithelial cells isolated from the susceptible breast (Susc, red bars) and matched healthy controls (HC, blue bars). Two-tailed *t* test is used to calculate *P* value. n.s. no significant *P* value; *P < 0.05; **P < 0.005.

The proliferation rate of the epithelial compartment in both susceptible and matched healthy breasts was determined by evaluating the expression level of *MKI67* and *PCNA* in the RNA-seq dataset and by performing immunostaining with Ki67 (Fig. 4a, b). No difference in cell proliferation was detected between the two cohorts.

Genes involved in lipid metabolism processes (Tables 3 and 4) and highly differentially expressed between the two experimental groups (*P* < 0.001) were selected for further validation. These targets included AKR1C1, AQP7, CD36, LIPE, among the upregulated genes, and ZNP57 and SNORA13, among the downregulated genes (Fig. 4c).

Both SNORA13 and ZNP57, even though highly downregulated in the RNA-seq data (fold change −4.3; *P* = 0.006 and fold change −29.9; *P* = 0.0004, respectively) showed similar expression levels in the two experimental groups in either qPCR or immunohistochemistry (IHC) assays (Supplementary Fig. 5). In contrast, qPCR analysis validated the upregulation of AKR1C1 in susceptible breast epithelium as compared with the microdissected epithelium from the healthy breasts (*P* = 0.03, Supplementary Fig. 5).

IHC analysis confirmed the upregulation of AKR1C1 (*P* < 0.0001), AQP7 (*P* = 0.012), CD36 (*P* = 0.002), and HSL (alias LIPE, *P* = 0.015) in the susceptible breast epithelium (Fig. 4d). Overexpression of AKR1C1 (*P* = 0.045), AQP7 (*P* = 0.014), CD36 (*P* = 0.014), and LIPE (*P* = 0.0007) was also detected by qPCR in primary epithelial cells isolated from the susceptible normal breast as compared with cells isolated from healthy breast tissue cores (Fig. 4e and Supplementary Fig. 5). Analysis of The Cancer Genome Atlas *(*TCGA) showed that AKR1C1, AQP7, CD36, and LIPE display low genomic alteration frequency in breast cancer, and only CD36 expression has prognostic value (*P* = 0.005) (Supplementary Fig. 6a–c). However, when evaluating the effect of the combination of each gene with CD36, LIPE-CD36 shows an inverse relationship, while AKR1C1-CD36 shows a direct relationship with disease-free survival (Supplementary Fig. 6d). Furthermore, these genes exhibit patterns of co-occurring genetic alterations (i.e., copy number) across multiple breast cancer patients, suggesting a functional interaction (Supplementary Fig. 6e)^19^.

### Immune phenotype alterations in the susceptible breast stroma

**Fig. 5:**
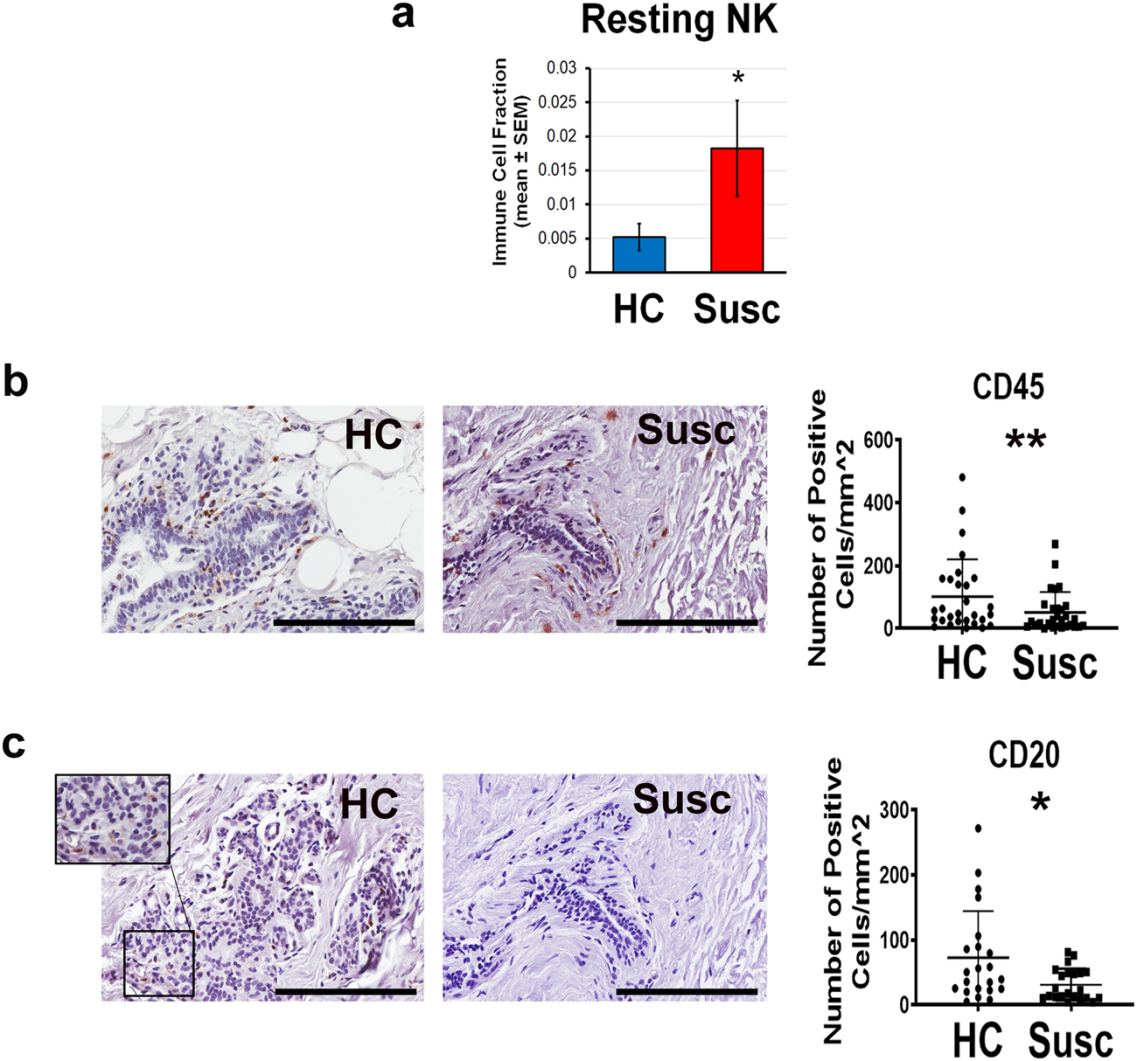
Immune cell profiling of the microdissected breast stroma. **a** CIBERSORT immune cell fractions were determined for each subject. The mean value for NK cell fraction was calculated for each group and compared using two-sided unpaired *t* test. **b** Immunohistochemical (IHC) representative images (×40 magnification; scale bar: 200 µm) and quantification of CD45 and **c** CD20 staining in susceptible and matched healthy (HC) breast tissue sections; each dot represents one subject. Quantification of the staining was performed using QuPath 0.2.0 and expressed as the number of positive cells per mm^2^. Error bars show the standard error of the mean. *P < 0.05; **P < 0.005.

Interestingly, pathway analysis showed that genes downregulated in both epithelium and stroma of the susceptible breast are involved in the immune response pathway. To better elucidate the immune phenotype occurring in the susceptible breast stroma as compared with the healthy controls, two approaches were employed: (1) immune cell profiling by deconvolution of the transcriptomic data revealed an increase in inactive or resting natural killer (NK) cells in the susceptible breast tissue as compared with the healthy breast (*P* = 0.004, Fig. 5a and Supplementary Table 13). (2) IHC staining of the breast tissue sections with generic immune cell markers showed a significant reduction of CD45+ cells in the susceptible as compared with healthy breasts (*P* = 0.003; Fig. 5b). Furthermore, while no change in CD68 (macrophage marker), CD4 and CD8 (T cell markers) staining was detected, the susceptible breast stroma showed a significant decrease in CD20+ B cells as compared with the controls (*P* = 0.002, Fig. 5c and Supplementary Fig. 7).

### Increased epithelium-adipose tissue crosstalk in susceptible breast

Finally, we applied the co-expression network approach to assess the crosstalk between the epithelial, stromal, and adipose tissue compartments in histologically normal breasts of either susceptible or healthy women^3^.

**Fig. 6:**
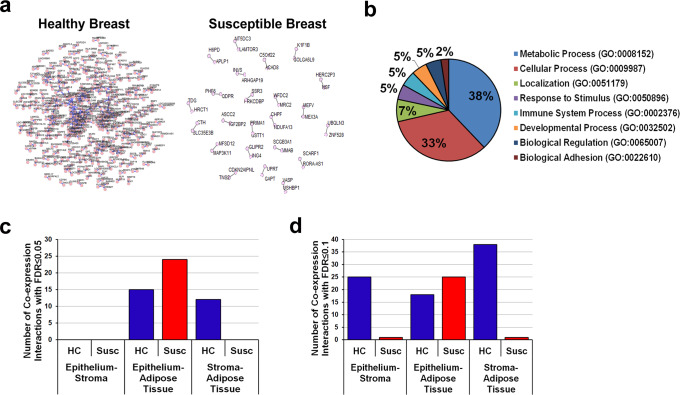
Epithelial–stromal-adipose tissue co-expression network analysis. **a** Connectivity networks including pairwise associations with a *P* value ≤1E-04 in susceptible and matched healthy controls. Each node in the network is a gene, and each edge represents a co-expression relationship. **b** Gene ontology of the genes included in the connectivity network in the susceptible breasts. **c** Pairwise co-expression interactions between epithelial, stromal, and adipose tissue mRNA levels in susceptible (Susc) and matched healthy controls (HC) with FDR < 0.05 and **d** FDR < 0.1).

Genes in at least two of the three pairwise associations (with a *P* value ≤1E-04) were used to construct the connectivity networks (Fig. 6a), where the nodes represent genes and the edges reflect highly similar profiles. The number of nodes in the susceptible breast is smaller than that observed in the normal breast (Fig. 6b). Analysis of the networks in the susceptible breast uncovers a set of genes involved in nucleic acid (i.e., KIF1B, NSF, UPRT) and protein metabolism (i.e., UBQLN3, INVS, CTH, GSTT1), and cell–cell interaction (i.e., ARHGAP19, TNS2, SSR3, IGF2BP2) (Fig. 6c).

We compared the number of pairwise associations between the transcriptome profiles of the three tissue compartments with FDR ≤ 0.05 and a less stringent FDR ≤ 0.1 in both the susceptible group and healthy controls. Overall, when examining epithelium–stroma and stroma–adipose tissue associations, we identified a higher number of connections in the healthy samples as compared with the susceptible group. However, the susceptible cohort showed an increase in epithelium-adipose tissue connectivity (*n* = 25) as compared with the healthy control group (*n* = 18) (Fig. 6d and Supplementary Table 14). In the normal breast, the set of most highly connected genes between epithelium and stroma included genes involved in the negative regulation of the immune system (BATF3, FOXF1, DCST1) and amino acid metabolism (COL3A1, COL5A2), while the gene networks in epithelium-adipose tissue are linked with carbohydrate metabolism (UGT2B11, UGT2B12) and endoplasmic reticulum-associated degradation pathway (DERL2, YOD1). In the susceptible breast, COLGALT2 was the most connected gene in the epithelial–stromal co-expression network and contributed to the network primarily through its stromal expression, while between epithelium and adipose tissue we found genes involved in metabolism (CA6, CYP24A1) and intracellular signaling (CR2, IL22RA1).

Taken together, these results support that the rise in epithelial-adipose tissue self-loops represents an important property of the inter-compartmental interactions that occur in the early phase of carcinogenesis.

## Discussion

NAT tissues are frequently designated as healthy control samples for cancer studies under the assumption that histological normalcy implies biological normalcy. However, these tissues have many morphologic and phenotypic distinctions from non-tumor-bearing healthy tissue, including pH levels, allelic imbalance and telomere length, stromal behavior, and transcriptomic and epigenetic aberrations^7^. Little is known about the transcriptomic profile of these regions before the clinical manifestation of the tumor. Here, we investigated the molecular features of the breast prior to cancer diagnosis (labeled susceptible normal). We show that the microdissected epithelium from the susceptible normal breast has a transcriptome profiling significantly different from the paired contralateral breast tissue, but similar to that of the NAT. The differences between susceptible and NAT are limited to genes involved in metabolic processes. We further show that the breast tissue susceptible to cancer development has an enrichment in lipid metabolism-related genes not only in the epithelial compartment but also in the stroma and adipose tissue, when compared to the breast tissue of healthy women.

Tumor cells are exposed to a myriad of altered forces and signals from the surrounding microenvironment that dramatically modify their behavior and therefore tumor development^5^. Ma et al. showed that both breast epithelial and stromal compartments undergo extensive gene expression alterations at the pre-invasive stage of DCIS as compared with normal breast tissue. The authors propose a key role of cell–cell communication between these two compartments during tumor progression^20,21^. Our study constitutes a more comprehensive analysis of earlier phase of breast cancer development (prior to the clinical manifestation of the tumor) through the separation and profiling of the three functional compartments of the breast tissue: epithelium, stroma, and adipose tissue. Moreover, while previous studies^7,8,22^ utilized breast tissues from either reduction mammoplasty or adjacent to the tumor lesion as healthy controls, we included a proper cancer-free breast tissue, from age-, BMI-, menopausal status-matched healthy volunteers as comparative controls for the susceptible breast.

Our comprehensive transcriptomic analysis of susceptible histologically normal breast provides direct evidence of a breast cancer-associated metabolic rewiring of breast tissue affecting not only the epithelial cells but also the stroma and adipose tissue of the breast.

The reprogramming of lipid metabolism is a hallmark of many cancers, including breast cancer. While healthy cells preferentially uptake exogenous fatty acids, cancer cells also synthesize fatty acids de novo by increasing the expression of fatty acid biosynthetic enzymes^23^. In our study, we detected an upregulation of fatty acid metabolism-related genes in the susceptible breast epithelial compartment (i.e., LIPE, AQP7, CD36, PLIN1, ADIPOQ) as well as in the stromal compartment (i.e., ACAA1, FASN, GPD1, LPL, and LIPE), and of genes involved in fatty acid and amino acids transport (i.e., RIMS2, FABP4, and SLC6A13) in the adipose tissue. This expression profile is indicative of the induction of lipogenesis and fatty acid transport and, as shown in the upstream regulator analysis, is connected with the peroxisome proliferator-activated receptor (PPAR) α/γ signaling pathway.

PPARγ, which we found slightly upregulated in both susceptible epithelium (fold change:1.3, *P* = 0.049) and stroma (fold change: 1.4, *P* = 0.010), is overexpressed in several tumors including breast^24^. Its role in tumorigenesis is controversial^25^. Our data, corroborated by findings from Apostoli et al.^25^ and Nakles et al.^26^, suggest the biphasic action of PPARγ in breast cancer development, with a pro-tumorigenic role in the early phase of malignant transformation.

In this study, the breast tissue susceptible to cancer development shows an increase in genes linked to adaptation to oxidative stress (i.e., AKR1C1) and accumulation of fatty acids (i.e., LIPE, CD36, AQP7), which are used by the cells for biomass synthesis, cell proliferation, migration, and invasion. CD36 and AQP7, which mediate the transport of fatty acids and water/glycerol, respectively, are expressed in tumors including breast cancer and are involved in cell migration and tumor metastasis^27,28^. CD36 knock-out or treatment with an anti-CD36 monoclonal antibody reduced tumor burden in ovarian cancer cells mouse xenografts^29^. Interestingly, Ladanyi et al. showed that CD36 expression was induced in ovarian cancer cells upon co-culture with primary human omental adipocytes, facilitating exogenous fatty acids uptake^29^.

AKR1C1 and HSL are involved in steroid metabolism^30,31^. AKR1C1, with its ability to metabolize reactive aldehydes, has a key role in protecting transformed cells from the reactive oxygen species generated from lipid peroxidation secondary to increased lipid metabolism^32^. AKR1C1’s inhibition, through mefenamic acid treatment, restored cancer cells’ sensitivity to cisplatin and 5-fluoruracil^33^. HSL, through the cleavage of stored cholesteryl esters into cholesterol and free fatty acids, may generate endogenous oxysterols or oncometabolites with an important role in cancer promotion and progression^34^.

An increase in genes associated with lipid metabolism was also found in the contralateral unaffected breasts of women with unilateral ER-negative breast cancer^35^. Moreover, a recent report by Madak–Erdogan identified 25 plasma metabolites that discriminated between healthy and susceptible postmenopausal women and therefore may represent new circulating biomarkers of breast cancer risk^36^. The authors showed that, prior to diagnosis of breast cancer, postmenopausal women had significantly higher levels of lipolysis byproducts, free fatty acids (palmitic, arachidonic, stearic, linoleic, oleic acids), and glycerol in their plasma as compared with healthy controls. Free fatty acids also stimulated the proliferation and growth of estrogen receptor (ER)+ breast cancer cells.

In the susceptible breast stroma, we observed a downregulation of genes involved in the immune response, an increase in resting NK-related gene signature and a reduction in the IHC staining of CD45 (generic immune marker) and CD20 (B-cell marker) cells. The number and function of the local immune cells can be affected by metabolites released in the surrounding microenvironment^37–39^. Recent reports indicate that breast cancer cells may escape immune surveillance by releasing free fatty acids, which inhibit cytotoxic T cells^40^. Moreover, animal studies and human trials of PPARγ-activating drugs, normally used to treat diabetes, have shown these compounds to have great potential as anti-inflammatory drugs^41^. It has been suggested that some of the protective anti-inflammatory effects of dietary n-3 PUFAs is mediated through PPARγ activation^42^. The altered metabolism in the tumor cells, as well as in surrounding cells, may create an immunosuppressive environment that prevents host immune cells from detecting and eliminating cancer cells.

The adipose tissue, in addition to its ability to store energy reserves as triglycerides, is now recognized as an actual organ with both metabolic and endocrine functions (reviewed in ref. ^43^). It is a heterogeneous tissue, including mature adipocytes (about 50%), stem cells, pre-adipocytes, fibroblasts, endothelial cells, nerve cells, and macrophages. The adipose tissue in the susceptible normal breast showed the upregulation of molecules involved in the transport of fatty acids and amino acids, including RIMS2 and FABP4, as compared with the matched healthy breast. RIMS2 (aka RIM2, regulating synaptic membrane exocytosis 2), is a key molecule of the cAMP-dependent exocytotic machinery, and regulates the secretion of adiponectin^44^. Its overexpression in pancreas cancer was previously reported^45^. FABP4 is predominantly expressed in macrophages and adipose tissue, where it regulates adipocyte differentiation, fatty acids storage, and lipolysis, and is an important mediator of inflammation^46^. FABP4 is also expressed at a higher level in cancer cells, and its upregulation promotes tumor growth^47,48^.

Our epithelial–stromal–adipose tissue co-expression analysis shows epithelial-adipose tissue co-expression network self-loops to be highly enriched among the most significant interactions in susceptible breasts as compared with the healthy controls. These data suggest an increase in the crosstalk between epithelial and adipose tissue compartments, and a key role of the latter, in the early phase of breast cancer development. The networks connecting the susceptible epithelium and adipose tissue include genes involved in metabolism. Although the data show a limited role of the stroma in breast cancer susceptibility, whether the epithelium and the adipose tissue in the susceptible breast are in direct or indirect contact is still unclear and requires further investigation. Our finding supports the recent observations promoting a model where adipocytes respond to cancer cell-derived endocrine and paracrine signaling to provide metabolic substrates, which in turn drive enhanced cancer cell proliferation, invasion, and treatment resistance^49^.

Overall, our data suggest a metabolic activation occurring in the susceptible normal breast, centered on lipolysis and local immunosuppression, which may promote the survival and proliferation of a transformed cell. However, several limitations of this study warrant discussion. First, our sample size is modest due to the extended time required for developing the resource, and additional studies with larger sample sizes and multiple centers are needed to clarify our results. Either a more precise separation of the interlobular and intralobular stroma, two sub-compartments structurally and functionally different, or a single-cell analysis of the breasts may unveil interesting transcriptional clues of breast cancer susceptibility. We are currently expanding our investigation to postmenopausal susceptible women and donors from different racial and ethnic backgrounds. Merging transcriptomic data with metabolomics and investigating the microbial dysbiosis in the breast tissue of women at high risk of developing breast cancer are other interesting lines of research that we intend to pursue^50^. By overcoming significant challenges and continuing to collect breast tissue cores from healthy women, we will improve this unique resource, allowing the generation of additional statistically meaningful data.

In summary, our data demonstrate that upregulation of genes involved in lipid metabolism and adipogenesis occurs in the breast prior to the clinical manifestation of the malignancy and that the adipose tissue in the breast plays a critical role in tumorigenesis through an active crosstalk with the epithelial compartment. Because of the characteristics of the study cohort, these findings are relevant to premenopausal women without distinction made in terms of the clinicopathological features of the tumor later detected. Defining the mechanisms that are the underpinning of the dynamic interaction between stromal adipocytes and breast cancer cells, especially in the context of obesity, may identify novel therapeutic targets and approaches.

## Methods

### Participants and samples

This was a case-controlled study of a total of 87 women. The study cohort consisted of 2 women who donated breast tissue cores prior to and post-cancer diagnosis (paired specimens include the susceptible normal breast tissue, NAT, and contralateral normal breast tissue cores), 38 women donating susceptible normal (prior to tumor detection), and 47 matched women with no history of breast cancer who donated healthy breast tissues that were used in this study as matched healthy controls. Table 1 and Supplementary Table 1 list the characteristics of the subjects. Specimens were obtained from the Susan G. Komen Tissue Bank at the IU Simon Cancer Center (KTB). All the samples were collected from voluntary donors upon written informed consent. Subjects were recruited under a protocol approved by the Indiana University Institutional Review Board (IRB protocol number 1011003097 and 1607623663) and according to The Code of Ethics of the World Medical Association (Declaration of Helsinki).

Susceptible donors were identified through an annual medical follow-up as individuals who had a breast cancer diagnosis post-tissue donation and lacking of any mutation in known breast cancer predisposition genes upon genetic testing. The subjects in the susceptible normal and healthy controls cohorts were matched (at a ratio of either 1:2 or 1:3) according to age, racial background, body mass index, and follow-up time, the latter defined as the interval from the date of tissue donation to last medical follow-up. Hematoxylin and eosin-stained sections of either formalin-fixed or PAXgene-fixed paraffin-embedded breast tissue cores were reviewed by a pathologist to confirm the absence of histological abnormalities.

### Breast tissue microdissection and RNA extraction

The transcriptome profiling was performed on breast tissue cores from 25 premenopausal (mean age 45 years) KTB donors, including two women who donated breast tissue biopsies prior to and post diagnosis, 7 susceptible, and 16 matched healthy women. Tables 1 and 2 include the clinical characteristics of these subjects. Breast tissues from premenopausal women were chosen over those from postmenopausal women for their higher epithelial cellularity. Moreover, in terms of clinical impact, in recent decades, incidence rates of advanced breast cancer have increased among premenopausal women, whereas they have consistently decreased among older women^51^. In order to examine each breast tissue compartment and the tissue inter-compartmental crosstalk, microdissection of the breast tissue sections was performed. Fresh-frozen breast tissue cores (≈80 mg) were first embedded in OCT, sectioned (8 µM thickness) on PEN Membrane frame slides (Leica Microsystems, Buffalo Grove, IL). Slides were stored at −80 °C prior to dissection. Three slides were removed from the freezer at a time and were stained using the HistoGene LCM Frozen Section Staining Kit (Arcturus, Life Technologies, Carlsbad, CA, USA). The three tissue compartments were microdissected using a laser microdissection microscope (LMD 6500, Leica Microsystems). Previous study reports a lack of transcriptional differences between the interlobular and intralobular stroma, being limited to the level of specific proteins^52^; here these locations were not specifically differentiated by the microdissection. All dissections were completed within an hour of thawing to minimize RNA degradation. Supplementary Fig. 1 shows the measurement of the microdissected area of each tissue compartment. Out of the 23 premenopausal breast tissues, only 16 (5 susceptible and 11 healthy controls) included sufficient microdissected adipose tissue for the RNA-sequencing analysis. In all susceptible samples, the adipose tissue represented only 12.4% (range: 0–26%) of the entire tissue and therefore its influence on the global breast tissue transcriptome is limited. Moreover, in order to reduce any confounding factors, the subjects were matched also for BMI. The total RNA from the microdissected epithelial compartment was extracted using the Allprep DNA/RNA/miRNA Universal Kit (QIAGEN, Germantown, MD), while total RNA from the microdissected stroma and adipose tissue was extracted using RNeasy Mini kit (QIAGEN).

### Whole-transcriptome sequencing

The RNA-sequencing (RNA-seq) was conducted at the Center for Medical Genomics at Indiana University. The concentration and quality of the total RNA was assessed using Agilent 2100 Bioanalyzer. A RIN (RNA Integrity Number) ≥5 was required to pass the quality control. Then 10 ng of RNA per sample were used to prepare a dual-indexed strand-specific cDNA library using Clontech SMARTer RNA Pico Kit v2. The resulting libraries were assessed for their quantity and size distribution using Qubit and Agilent 2100 Bioanalyzer. Two hundred picomolar pooled libraries were utilized per flowcell for clustering amplification on cBot using HiSeq 3000/4000 PE Cluster Kit and sequenced with 2 × 75 bp paired-end configuration on HiSeq4000 (Illumina, San Diego, CA) using HiSeq 3000/4000 PE SBS Kit. A Phred quality score (Q score) was used to measure the quality of sequencing. More than 90% of the sequencing reads reached Q30 (99.9% base call accuracy). Samples were sequenced as a single batch to avoid systematic differences linked to the batched effect. The sequencing data were first assessed using FastQC (Babraham Bioinformatics, Cambridge, UK) for quality control. Transcriptome sequencing recovered ~36–40 million raw reads from each of the breast specimens. After normalization, ~23–30 million reads were uniquely mapped using STAR v2.5 and UCSC hg19 as reference genome^53^ and ~9–14.6 million reads were assigned to annotated genes. Quality control of sequencing and mapping results was summarized using MultiQC^54^. The datasets generated and analyzed during the current study are available in the Gene Expression Omnibus (GEO) repository (accession number GSE141828)^55–57^.

### Data analysis

#### Differential expression

Genes with read count per million (CPM) > 0.5 in more than 2 of the samples were kept. The data were normalized using TMM (trimmed mean of M values) method. Differential expression analysis was performed using EdgeR^58^. PCA (principal component analysis) plots were made to identify and remove any potential outliers. False discovery rate (FDR) was computed from *P* values using the Benjamin–Hochberg procedure. Gene ontology and pathway analysis: PANTHER GO (http://www.pantherdb.org/) tool was used to perform gene ontology enrichment analysis. Ingenuity Pathways Analysis (IPA, Qiagen, Redwood City, CA) was used for the canonical pathway, upstream regulator, and gene network analyses^59^. Analysis of TCGA was performed by interrogating both cBioPortal (https://www.cbioportal.org/) and UALCAN (http://ualcan.path.uab.edu/) databases^60^. Data deconvolution: CIBERSORT algorithm (https://cibersort.stanford.edu^61^) was employed for the deconvolution of RNAseq global gene expression data in order to analyze the immune landscape of the breast microenvironment. The CIBERSORT values generated were defined as fractions of total leukocyte content per sample. Co-expression networks: For each tissue category (susceptible and healthy control), we used a linear regression to build univariate modes linking transcriptome profiling in one breast tissue compartment with that in another as previously described^3^. To construct co-expression networks, we computed all pairwise co-expression interactions between epithelial, stromal and adipose tissue mRNA levels, generating epithelial–stromal, epithelial-adipose tissue, and stroma–adipose tissue co-expression networks, where each node in the network is a gene, and each edge represents a co-expression relationship.

### Quantitative real-time polymerase chain reaction (qPCR)

Because of the lower yields associated with the tissue microdissection, only the RNA extracted from the microdissected epithelium was sufficient for the post-RNA-sequencing qPCR validation of two targets: AKR1C1 and SNORA13. qPCR was also used to detect the expression of selected targets in primary epithelial cells. Reverse transcription was performed using SuperScript™ IV VILO™ Master Mix (Invitrogen cat#: 11756050) according to the manufacturer’s instructions. qPCR was performed using the TaqMan™ Universal PCR Master Mix (Applied Biosystems, cat# 4304437) and the following TaqMan Gene Expression Assays (Applied Biosystems/ThermoFisher Scientific, Grand Island, NY): ACTB (Hs99999903_m1), AKR1C1 (Hs04230636_sH), SNORA13 (Hs03309450_s1), AQP7 (Hs00357359_m1), LIPE (Hs00943410_m1), CD36 (Hs00354519_m1), PCNA (Hs00427214_g1), MKI67 (Hs04260396_g1). qPCR reactions were run on a StepOne Plus Real-Time PCR System (Applied Biosystems/ThermoFisher Scientific) and data analyzed using the StepOne Software v2.3 (Applied Biosystems). Relative quantification was calculated with reference to either ACTB and analyzed using the comparative C_T_ method^62^. qPCR experiments were performed in triplicate.

### Immunohistochemistry (IHC)

The IHC validation cohort included 38 susceptible and 47 age-matched healthy controls Additional file 7: Supplementary Table S1 lists the clinical characteristics of the study cohort. IHC staining of 5-µm-thick formalin-fixed, paraffin-embedded tissue sections was performed using the MACTH4 universal HRP-Polymer Kit following the manufacturer’s instructions (Biocare Medical, M4U534L). Briefly, 5-μm-thick formalin-fixed, paraffin-embedded tissue sections were deparaffinized with three successive passages through xylene, and rehydrated through decreasing concentrations (100, 95, 80, 70, and 50%) of ethanol. After antigen retrieval, peroxidase, and protein blocking steps, tissue sections were incubated for 1 h at room temperature with primary antibodies, 30 min with the post primary block reagent, and 30 min with the horseradish peroxidase-coupled polymer secondary antibodies. Upon two additional washes, secondary antibodies were revealed with the liquid DAB Substrate Chromogen System (10-min incubation). Finally, slides were washed in distilled water, and counterstained with hematoxylin. Antibodies specific for AKR1C1 (GeneTex, GTX105620, 1:100), HSL (ThermoFisher Scientific, PA5-2638, 1:250), CD36 (Sigma, HPA002018, 1:500), AQP7 (Novus, NBP1-30862, 1:1000), CD45 (Dako, M0701, 1:100), CD68 (Dako M0876, 1:50), ki67 (Dako M7240, 1:50), CD4 (Leica Biosystems, NCL-L-CD4-368, 1:50), CD8 (Dako, M7103, 1:200), CD20 (Dako, M0755, 1:200), LC3B (GeneTex, GTX116080, 1:500), TOMM20 (Abcam, ab56783, 1:150) were used. Whole slide digital imaging was performed using the Aperio ScanScope CS system (Aperio, Vista, CA). Aperio Image Scope v12.3.2 software was used to quantify the signal of the epithelial staining using a positive pixel count algorithm, while QuPath v0.2.0 software was used to obtain an automatic count of the cells positive to CD45, CD68, CD8, CD4, and CD20^55,63^.

### Primary breast epithelial cells: cultures and immunofluorescence

Cryopreserved breast tissue cores were obtained from the KTB. Primary epithelial cells were isolated and cultured as previously described^64^. Human fibroblasts were obtained by incubating 1 ml of cell suspension after tissue dissociation in DMEM (Gibco, ThermoFisher Scientific) with 10% fetal bovine serum (Gibco). After 1 week of culture, 5000 cells were plated into each well of an eight-well-chamber slide (BD Biosciences, San Jose, CA). After overnight incubation at 37 °C, the cells were washed with PBS and fixed with acetone: methanol (1:1) at −20 °C for 10 min. After three washes with PBS, cells were incubated with blocking buffer (PBS1×, 5% normal goat serum, 0.1%TritonX-100) for 1 h at room temperature, followed by incubation with primary antibodies: rabbit anti-vimentin (Cell Signaling, D21H3, 1:100) or mouse anti-E-cadherin (Cell Signaling, 14472, 1:50) overnight. Upon three washes with PBS, cells were incubated with secondary antibodies (goat anti-mouse Alexa Fluor 568 or goat anti-rabbit Alexa Fluor 488; ThermoFisher Scientific, 1:500) for 1 h at room temperature. After three washes with PBS, the coverslide was mounted using DAKO fluorescent mounting medium (S3023 Agilent, Santa Clara, CA), and the staining was visualized using a fluorescent microscope (Eclipse TS100, Nikon Instruments inc, Melville, NY).

### Statistical analysis

Data were expressed as means ± standard error of the mean (SEM). Matched susceptible and healthy samples were compared using the paired Wilcoxon signed-ranks test, two-tailed, paired *t* test or one-way ANOVA (GraphPad Prism, version 7.01, La Jolla, CA). Significant differences were denoted as follows: *P* < 0.05 (*), and <0.005 (**).

### Reporting summary

Further information on research design is available in the Nature Research Reporting Summary linked to this article.

### Supplementary information

Supplementary Information

Reporting Summary

## Data Availability

The processed RNA-sequencing data generated during this study are publicly available in Gene Expression Omnibus under the accession https://identifiers.org/geo:GSE141828^57^. The raw fastq files are publicly available in Sequence Read Archive under the accession https://identifiers.org/ncbi/insdc.sra:SRP236605^56^. Immunohistochemical quantification staining data and Supplementary Tables 1–14 are publicly available in the figshare repository: 10.6084/m9.figshare.12793700^55^. Data supporting Table 1, and Supplementary Tables 7–12, will be made available on reasonable request from the corresponding author. Ingenuity pathways analysis (IPA) datasets, supporting Supplementary Tables 7–12 are only available on the PI’s personal account on IPA. UALCAN and cBioPortal data analyzed during the study are publicly available on the cBioPortal and UALCAN databases as described in the figshare data record above.
