# Author Correction: Upregulation of lipid metabolism genes in the breast prior to cancer diagnosis

**DOI:** 10.1038/s41523-024-00649-z

**Published:** 2024-06-17

**Authors:** Natascia Marino, Rana German, Xi Rao, Ed Simpson, Sheng Liu, Jun Wan, Yunlong Liu, George Sandusky, Max Jacobsen, Miranda Stovall, Sha Cao, Anna Maria V. Storniolo

**Affiliations:** 1 Susan G. Komen Tissue Bank at the IU Simon Cancer Center, Indianapolis, IN 46202 USA; 2 Department of Medicine, Indiana University School of Medicine, Indianapolis, IN 46202 USA; 3 Department of Medical and Molecular Genetics, Indiana University School of Medicine, Indianapolis, IN 46202 USA; 4 Pathology and Laboratory Medicine, Indiana University School of Medicine, Indianapolis, IN 46202 USA; 5 Department of Biostatistics, Indiana University School of Medicine, Indianapolis, IN 46202 USA

**Keywords:** Breast cancer, Cancer epidemiology, Risk factors, Oncogenesis

Correction to: *npj Breast Cancer* 10.1038/s41523-020-00191-8, published online 06 October 2020

In this article, the author name Miranda Stovall was incorrectly written as Miranda Stoval. The original article has been corrected.

