# Supplementary material for: Upregulation of lipid metabolism genes in the breast prior to cancer diagnosis
Source: NPJ Breast Cancer. 2020 Oct 6;6:50. doi: 10.1038/s41523-020-00191-8 (PMC7538898; doi:10.1038/s41523-020-00191-8)
Supplement: Supplementary file 1 — Supplementary Information [file 41523_2020_191_MOESM1_ESM.pdf]

a

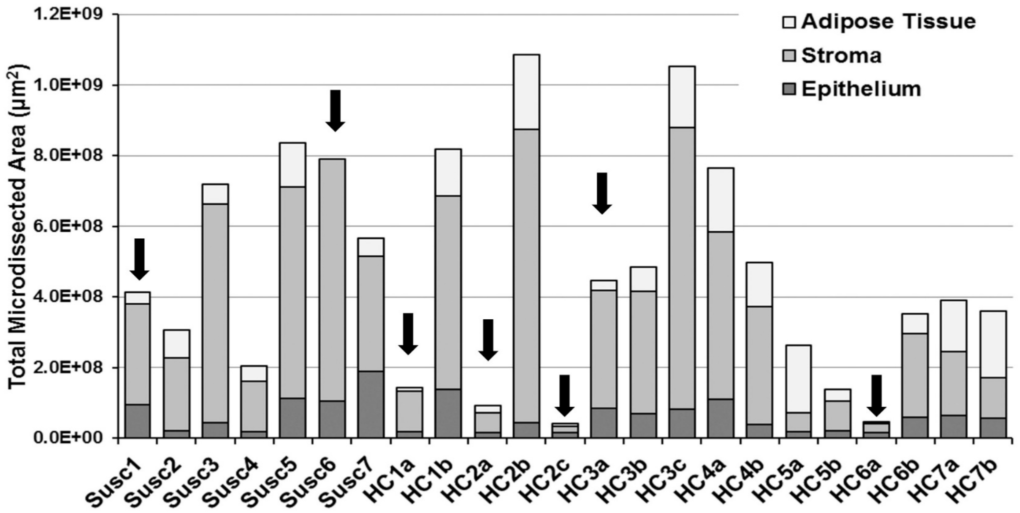

b

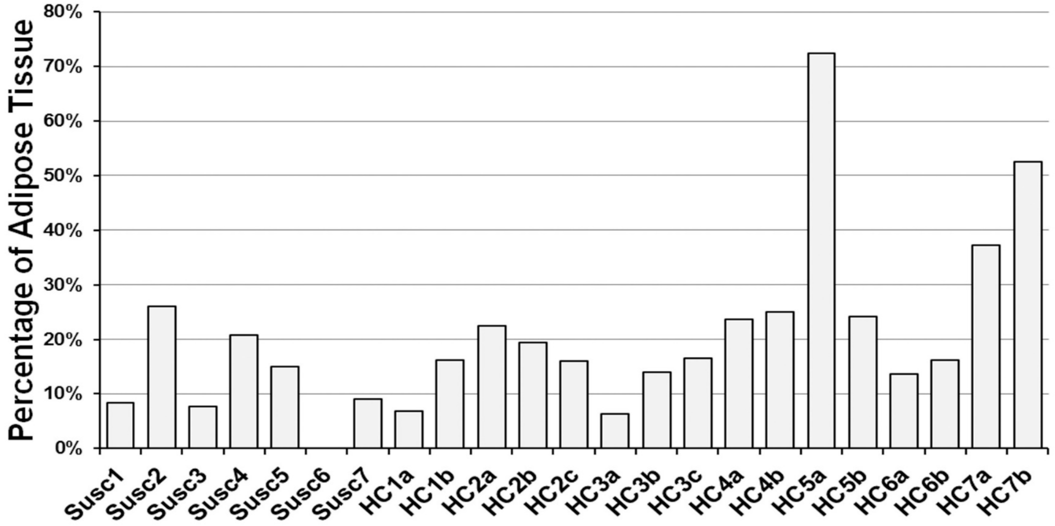

Supplementary Figure 1: Breast tissue microdissection. a) Epithelial, stromal and adipose tissue compartments were microdissected from 7 susceptible (Susc) and 16 healthy control (HC) breast tissue samples. Adipose tissue recovered from 6 breast tissue samples (black arrows) was insufficient for the transcriptomic analysis. **b**) Percentage of adipose tissue area in respect of the other tissue compartments in each microdissected sample. In all susceptible samples, the adipose tissue represented only 12.4% (range: 0-26%) of the entire tissue and therefore its influence on the global breast tissue transcriptome is limited.

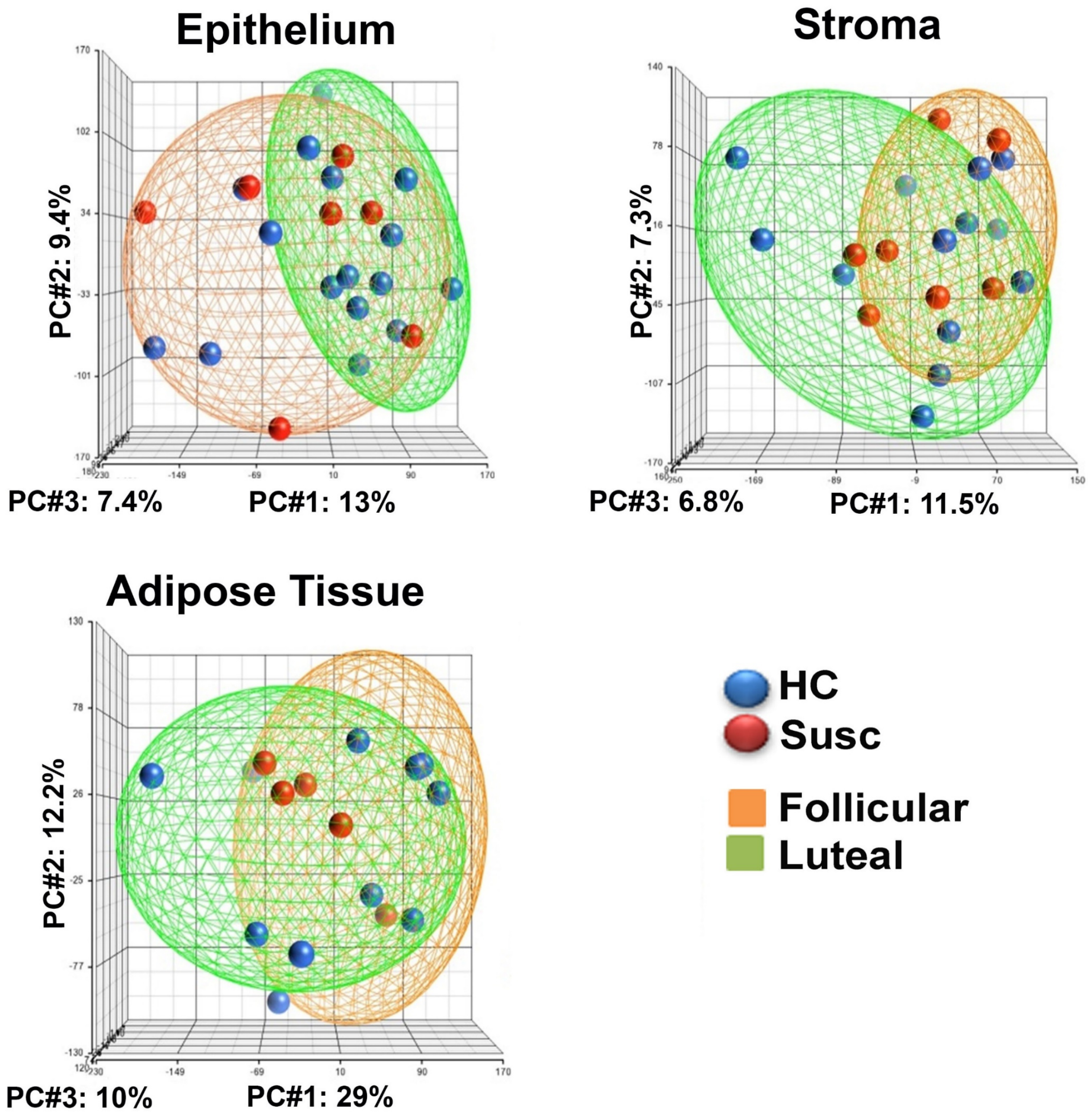

Supplementary Figure 2: Transcriptome profiling of the microdissected breast compartments. Principal component analysis (PCA) of the transcriptome profile of the microdissected breast epithelium, stroma and adipose tissue from breast tissue cores of susceptible (Susc, red circles) or healthy (HC, blue circles) women at either follicular (orange ellipsoid) or luteal (green ellipsoid) phase of the menstrual cycle.

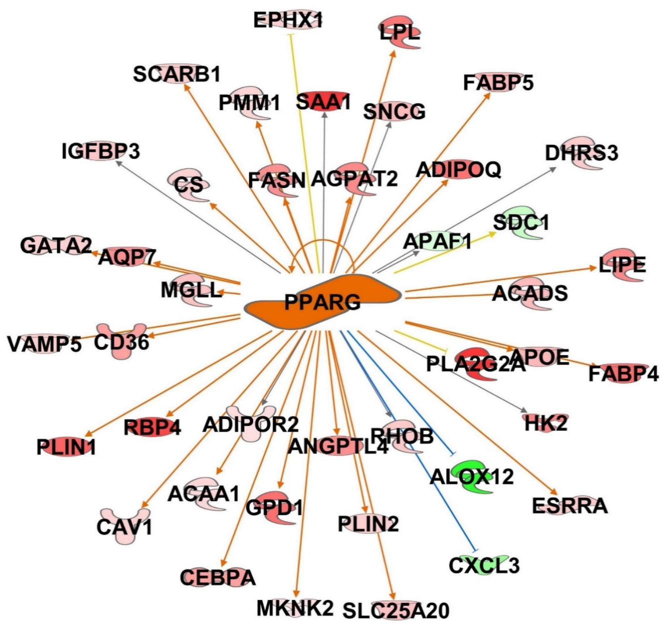

Supplementary Figure 3: Upstream regulator analysis of the breast stroma-related genes. Master regulator analysis (IPA v01-06) for the differentially expressed genes between susceptible and healthy control breasts in the stromal breast tissue compartment. Upregulated molecules in the susceptible stroma as compared with the healthy controls are in red, and the downregulated molecules are in green. Arrows indicate the intermolecular relationship: activation (orange), inhibition (blue), effect not predicted (gray), inconsistency with the state of downstream molecule (yellow).

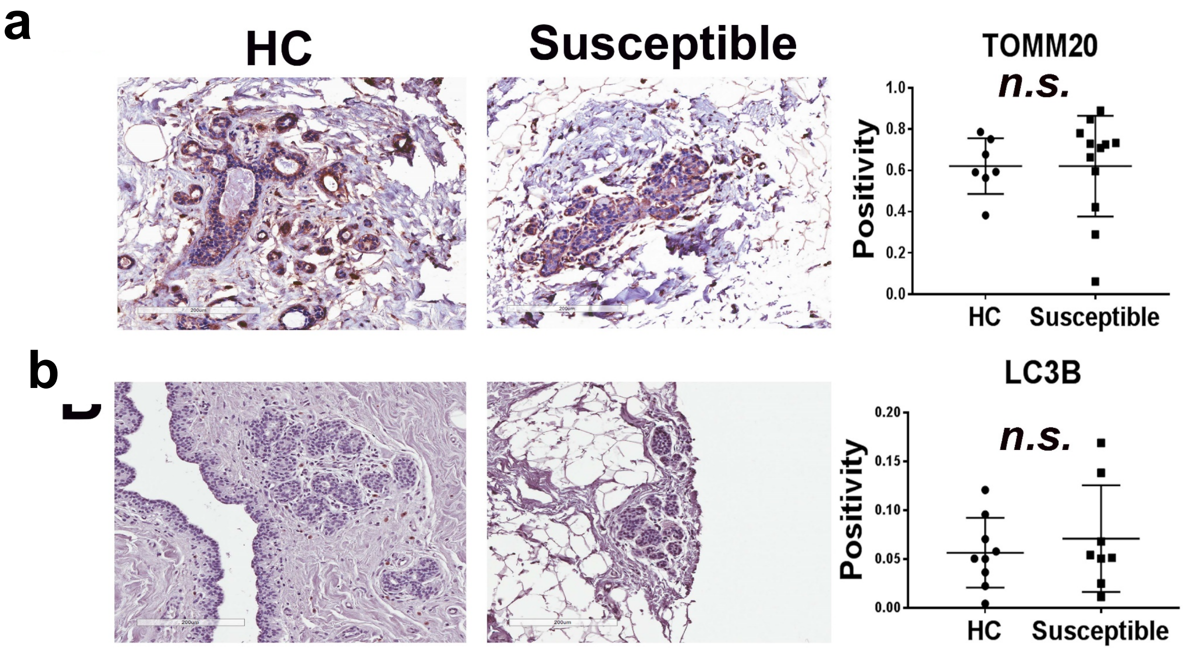

Supplementary Figure 4: Immunohistochemical evaluation of lipophagy markers. Representative images of the IHC staining with TOMM20 (**a**) and LC3B (**b**) antibodies on the paraffin-embedded breast tissue sections (µm) of healthy (HC) and susceptible women are shown on the left. Quantification of the staining, expressed as positivity (ratio of brown positive cells versus total hematoxylin stained nuclei), is shown on the right. Two-tailed t test is used to calculate *p*value *n.s.*: no significant *p*value.

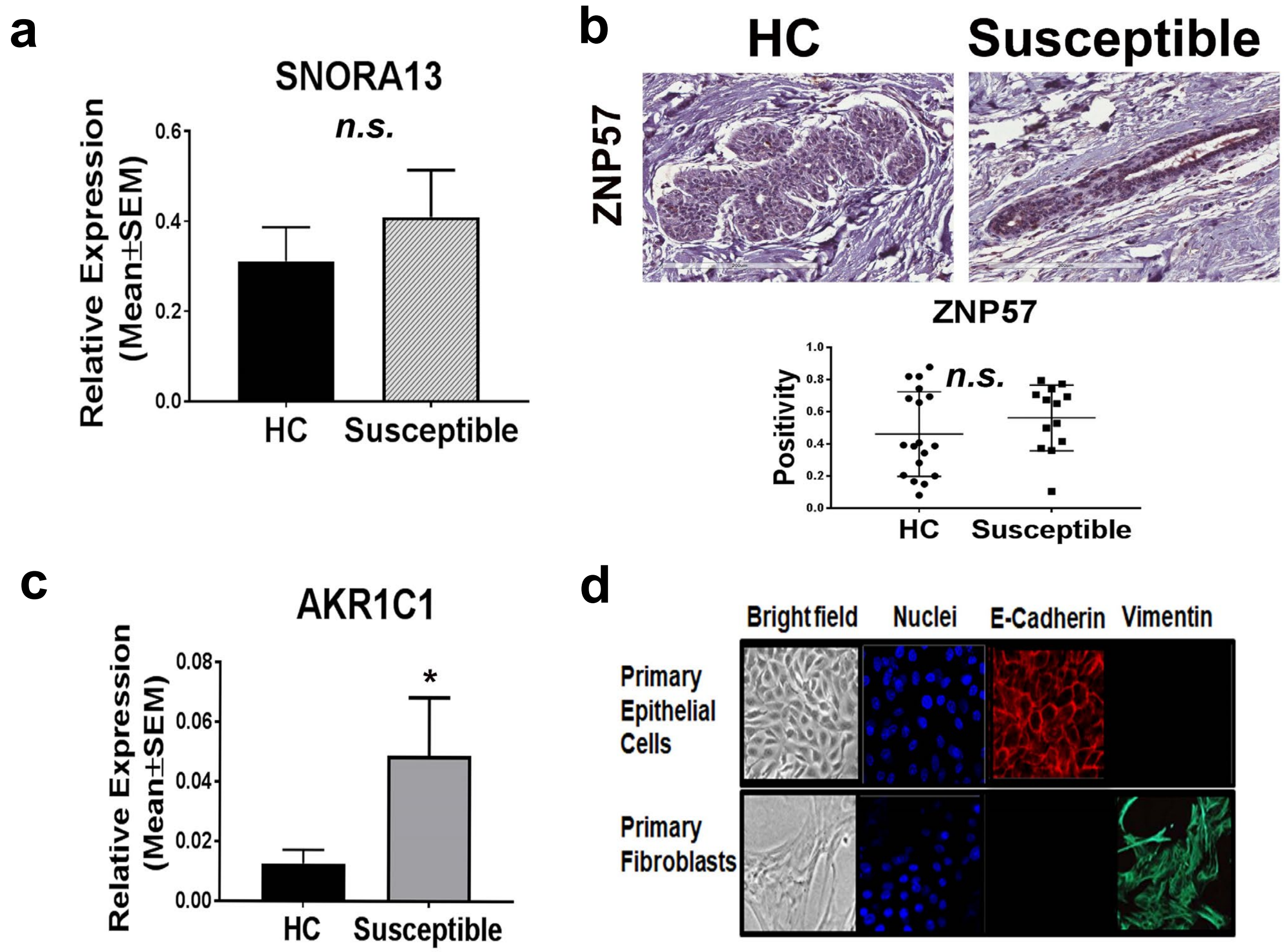

Supplementary Figure 5: Validation of the dysregulation of the selected targets. a) qPCR was used to detect the expression level of SNORA13 in microdissected epithelium from susceptible and healthy control (HC) breast samples. Two-tailed t test is used to calculate *p*value. **b**) IHC staining of ZNP57 in breast tissue sections of susceptible and healthy (HC) women. Matched susceptible and healthy samples were compared using the Wilcoxon matched-pairs signed ranks test. (**c**) Expression of AKR1C1 in the microdissected epithelium was evaluated using qPCR. **d**) Representative images of human primary epithelial cells (left) stained with antibodies for epithelial (E-cadherin, red), mesenchymal (Vimentin, green) lineage and nuclear dye (DAPI, blue). Human primary fibroblast (right) were used as staining control *n.s.*: no significant *p*value; *$p<0.05$.

a

Genomic alteration in AKR1C1, CD36, LIPE and AQP7 genes in Breast Cancer (N=9131, 14 Studies)			
	Cancer Type		
	Invasive Breast Cancer	Breast Cancer	Breast Sarcoma
AKR1C1	2.82%/0.86%*	2.27%/0.16%	
CD36	0.46%/0.46%	0.83%/0.21%	0%/3.7%
LIPE	0.73%/0.36%	0.35%/0.14%	
AQP7	0.91%/0.09%	0.48%/0.1%	

* Amplification frequency/ mutation frequency

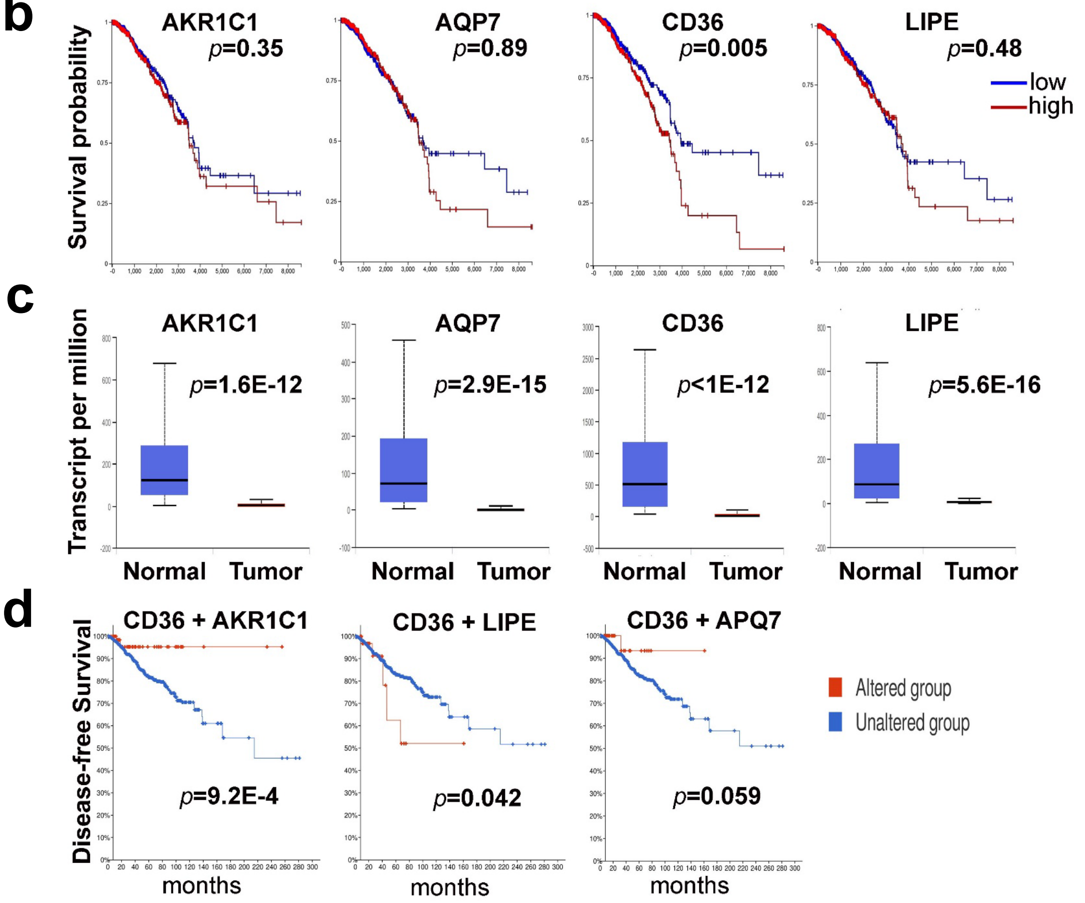

e

Mutual Exclusivity analysis of CD36,LIPE, AKR1C1 and AQP7 in Breast Cancer (N=9131, 14 studies)									
A	B	Neither A	Not B	B Not A	Both	Log2 Odds Ratio	p-Value	q-Value	Tendency
AKR1C1	CD36	8812	219	89	11	2.314	<0.001	<0.001	Co-occurrence
CD36	LIPE	8970	94	61	6	>3	<0.001	<0.001	Co-occurrence
LIPE	AQP7	9009	63	55	4	>3	<0.001	0.002	Co-occurrence
AKR1C1	AQP7	8847	225	54	5	1.864	0.016	0.024	Co-occurrence
AKR1C1	LIPE	8839	225	62	5	1.664	0.026	0.032	Co-occurrence
CD36	AQP7	8974	98	57	2	1.684	0.136	0.136	Co-occurrence

Supplementary Figure 6: TCGA analysis of AKR1C1, LIPE, CD36 and AQP7 in breast cancer. Genomic alterations (**a**), Kaplan Maier curves (**b**), relation of expression and breast cancer survival rate (**c**), Kaplan Meier using gene combination (**d**), and exclusivity analysis (**e**) were performed by using data from either UALCAN and cBioportal databases.

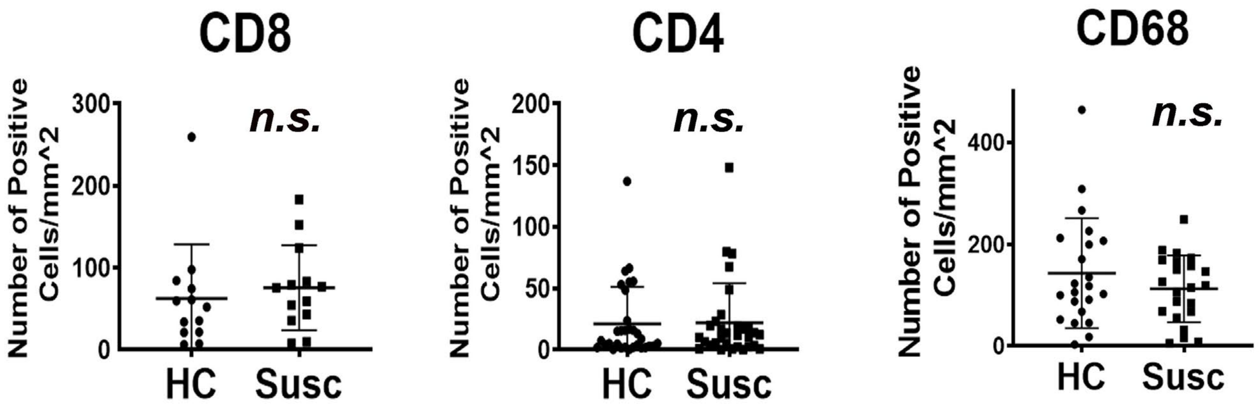

Supplementary Figure 7: Characterization of the breast stroma and adipose tissue. IHC staining quantification of CD4, CD8, and CD68 using quPath software. Each dot represents one subject. Comparison between the susceptible and healthy samples was evaluated using a two-sided paired *t*-test.

Supplementary Table 1: Genes differentially expressed between paired susceptible, normal adjacent to tumor (NAT) and contralateral breasts

		NAT vs Contralateral Normal		Susceptible Normal vs Contralateral Normal		Susceptible Normal vs NAT		log2RPKM					
GeneSymbol	GeneName	PValue	FDR	PValue	FDR	PValue	FDR	Adjacent Norm 1	Adjacent Norm 2	Contralateral Norm 2	Contralateral Norm 1	Susceptible Norm 1	Susceptible
PPIP5K1	diphosphoinositol pentakisphosphate kinase 1	1.9E-05	5.5E-03	1.4E-05	6.7E-04	9.5E-02	3.7E-01	1.38	1.01	-8.36	-8.36	0.86	0.77
TIMM8A	translocase of inner mitochondrial membrane 8A	5.2E-05	1.2E-02	1.4E-05	6.7E-04	6.8E-02	3.3E-01	3.21	2.86	-6.30	-6.30	2.64	2.55
FOXI1	forkhead box I1	6.8E-05	1.3E-02	9.7E-06	6.7E-04	2.0E-01	4.2E-01	2.99	1.09	-6.65	-6.65	3.09	3.00
CCDC167	coiled-coil domain containing 167	9.6E-05	1.7E-02	1.8E-05	6.7E-04	1.8E-01	4.1E-01	5.27	3.61	-4.76	-4.76	3.40	3.50
ZBTB42	zinc finger and BTB domain containing 42	3.1E-04	3.3E-02	9.4E-06	6.7E-04	1.9E-01	4.1E-01	1.61	1.03	-7.27	-7.27	1.68	1.60
NPL	N-acetylneuraminate pyruvate lyase	4.9E-04	4.0E-02	3.5E-06	6.7E-04	1.8E-01	4.1E-01	2.17	0.83	-7.11	-7.11	0.71	0.67
HAUS8	HAUS augmin like complex subunit 8	5.0E-04	4.1E-02	1.7E-05	6.7E-04	4.7E-01	4.9E-01	3.50	1.23	-5.99	-5.99	2.24	2.33
MRPL33	mitochondrial ribosomal protein L33	1.8E-06	9.5E-04	2.3E-05	6.7E-04	3.1E-01	4.6E-01	5.63	5.13	-4.50	-4.50	5.30	5.16
SPTBN4	spectrin beta, non-erythrocytic 4	4.0E-04	3.6E-02	2.4E-05	6.7E-04	3.1E-02	2.5E-01	0.45	-0.14	-8.64	-8.64	-1.04	-0.93
GATB	glutamyl-tRNA amidotransferase subunit B	5.5E-04	4.2E-02	2.6E-05	6.7E-04	2.5E-01	4.4E-01	0.20	2.63	-6.61	-6.61	2.48	2.35
ZNF669	zinc finger protein 669	6.4E-05	1.3E-02	3.1E-05	7.5E-04	2.0E-01	4.1E-01	3.13	2.50	-6.44	-6.44	2.53	2.39
PTRH1	peptidyl-tRNA hydrolase 1 homolog	4.4E-04	3.8E-02	4.2E-05	8.4E-04	2.8E-02	2.5E-01	3.64	2.78	-5.61	-5.61	1.51	1.38
AP3B2	adaptor related protein complex 3 beta 2 subunit	4.5E-04	3.8E-02	4.0E-05	8.4E-04	1.9E-01	4.1E-01	0.51	-0.06	-8.38	-8.38	-0.03	-0.18
ERICH3	glutamate rich 3	1.6E-08	3.4E-05	5.6E-05	8.6E-04	3.6E-01	4.7E-01	3.60	0.10	-8.29	-8.29	1.02	1.22
CRTAC1	cartilage acidic protein 1	2.5E-04	2.9E-02	5.5E-05	8.6E-04	8.4E-02	3.6E-01	2.42	1.39	-7.07	-7.07	0.88	0.72
ANKEF1	ankyrin repeat and EF-hand domain containing 1	5.2E-04	4.1E-02	5.3E-05	8.6E-04	3.0E-02	2.5E-01	1.90	1.77	-4.32	-4.33	2.26	2.13
AK7	adenylate kinase 7	5.8E-04	4.3E-02	5.0E-05	8.6E-04	1.8E-03	9.6E-02	1.41	1.32	-7.33	-7.33	-0.14	0.00
PDE6B	phosphodiesterase 6B	1.3E-04	2.1E-02	6.8E-05	9.3E-04	8.1E-02	3.5E-01	2.15	1.56	-7.28	-7.28	1.08	1.28
LRRC4C	leucine rich repeat containing 4C	4.6E-04	3.9E-02	6.7E-05	9.3E-04	1.2E-02	1.7E-01	1.26	1.42	-7.35	-7.35	0.48	0.66
PARP16	poly(ADP-ribose) polymerase family member 16	1.7E-04	2.3E-02	8.4E-05	1.0E-03	5.9E-02	3.2E-01	1.80	1.97	-6.88	-6.88	2.39	2.15
SLITRK5	SLIT and NTRK like family member 5	2.3E-04	2.7E-02	8.0E-05	1.0E-03	2.9E-02	2.5E-01	1.58	1.22	-7.60	-7.60	0.67	0.47
LOC101927895	uncharacterized LOC101927895	6.3E-04	4.5E-02	8.3E-05	1.0E-03	2.8E-03	1.2E-01	3.24	3.34	-5.36	-5.36	1.78	1.97
ZNF385B	zinc finger protein 385B	1.7E-04	2.3E-02	9.6E-05	1.0E-03	3.7E-01	4.7E-01	1.37	0.28	-7.97	-7.97	1.17	0.92
NSG1	neuronal vesicle trafficking associated 1	4.2E-04	3.7E-02	9.3E-05	1.0E-03	2.0E-02	2.2E-01	1.67	1.88	-6.94	-6.94	1.16	0.94
ACOT8	acyl-CoA thioesterase 8	2.0E-04	2.6E-02	1.3E-04	1.2E-03	3.9E-02	2.7E-01	3.68	3.13	-5.68	-5.68	2.50	2.25
LRRC37A3	leucine rich repeat containing 37 member A3	5.7E-04	4.3E-02	1.2E-04	1.2E-03	3.3E-01	4.6E-01	-0.36	1.17	-8.05	-8.05	-0.11	0.14
LIFR-AS1	LIFR antisense RNA 1	6.6E-04	4.6E-02	1.2E-04	1.2E-03	3.5E-01	4.6E-01	-0.81	1.66	-7.67	-7.67	0.85	1.13
FANCC	Fanconi anemia complementation group C	3.0E-04	3.2E-02	1.6E-04	1.4E-03	3.2E-01	4.6E-01	-0.63	1.45	-8.00	-8.00	1.13	0.81
NMNAT3	nicotinamide nucleotide adenylyltransferase 3	3.4E-04	3.4E-02	1.6E-04	1.4E-03	3.1E-01	4.6E-01	0.70	2.76	-6.94	-6.94	0.98	1.27
CADM2	cell adhesion molecule 2	3.7E-04	3.5E-02	1.6E-04	1.4E-03	4.7E-01	4.9E-01	-1.11	0.66	-8.76	-8.76	-0.31	0.00
UBXN8	UBX domain protein 8	4.4E-04	3.8E-02	1.6E-04	1.4E-03	3.4E-01	4.6E-01	3.60	1.48	-5.98	-5.98	2.18	1.89
RFTN1	raftlin, lipid raft linker 1	1.9E-04	2.5E-02	2.1E-04	1.7E-03	3.3E-01	4.6E-01	2.81	1.55	5.12	5.24	1.89	1.82
LINC00467	long intergenic non-protein coding RNA 467	5.1E-04	4.1E-02	2.5E-04	2.0E-03	1.7E-01	4.1E-01	2.97	3.65	-5.05	-5.05	3.59	3.99
ADPRM	ADP-ribose/CDP-alcohol diphosphatase, manganes	1.3E-04	2.1E-02	3.2E-04	2.4E-03	2.3E-01	4.4E-01	3.23	2.75	-6.05	-6.05	2.48	2.92
HSH2D	hematopoietic SH2 domain containing	2.9E-04	3.2E-02	3.3E-04	2.4E-03	5.9E-02	3.2E-01	2.33	1.31	-7.13	-7.13	0.17	0.56
TPRG1	tumor protein p63 regulated 1	1.7E-06	9.5E-04	3.6E-04	2.6E-03	1.1E-01	3.9E-01	2.91	2.50	-7.32	-7.32	1.86	2.37
NDUFAF4	NADH:ubiquinone oxidoreductase complex assemb	6.6E-05	1.3E-02	4.1E-04	2.8E-03	2.6E-01	4.6E-01	2.41	2.56	-6.72	-6.72	2.54	2.02
SGPP2	sphingosine-1-phosphate phosphatase 2	7.9E-05	1.5E-02	4.1E-04	2.8E-03	9.1E-02	3.7E-01	1.33	-0.41	-7.83	-7.83	2.60	2.02
RNF165	ring finger protein 165	9.4E-05	1.7E-02	4.3E-04	2.9E-03	2.4E-01	4.4E-01	1.38	0.39	-8.27	-8.27	0.15	0.65
CENPW	centromere protein W	3.8E-04	3.5E-02	4.4E-04	2.9E-03	2.4E-01	4.4E-01	1.78	0.72	-7.45	-7.45	0.98	0.50
MUC15	mucin 15, cell surface associated	1.6E-05	5.1E-03	4.5E-04	2.9E-03	5.0E-01	5.0E-01	3.68	-0.04	-7.21	-7.21	2.07	1.53
PAK5	p21 (RAC1) activated kinase 5	7.0E-05	1.4E-02	4.8E-04	2.9E-03	1.1E-02	1.7E-01	1.72	1.91	-7.71	-7.71	0.32	-0.16
NXT2	nuclear transport factor 2 like export factor 2	1.6E-04	2.2E-02	4.8E-04	2.9E-03	1.4E-01	4.0E-01	0.67	2.13	-7.02	-7.02	2.89	2.30
B4GALT6	beta-1,4-galactosyltransferase 6	7.0E-04	4.8E-02	4.8E-04	2.9E-03	9.7E-02	3.7E-01	1.24	1.53	-4.94	-4.95	1.12	0.75
EPB41L4B	erythrocyte membrane protein band 4.1 like 4B	4.0E-04	3.6E-02	5.2E-04	3.0E-03	1.6E-01	4.0E-01	1.40	1.97	-2.67	-2.69	1.96	2.27
APOD	apolipoprotein D	9.4E-06	3.5E-03	5.6E-04	3.2E-03	2.3E-01	4.4E-01	10.61	9.90	7.94	7.83	10.50	10.65
ZDHHC13	zinc finger DHHC-type containing 13	1.5E-05	4.7E-03	5.9E-04	3.3E-03	2.5E-01	4.4E-01	2.39	3.33	-6.73	-6.73	2.08	2.71
ZNF222	zinc finger protein 222	5.5E-04	4.2E-02	6.2E-04	3.4E-03	4.8E-01	4.9E-01	0.87	3.31	-6.23	-6.23	1.72	2.30
ZNF582	zinc finger protein 582	3.5E-04	3.4E-02	6.7E-04	3.6E-03	2.5E-01	4.5E-01	1.28	1.97	-7.10	-7.10	0.95	1.56
BID	BH3 interacting domain death agonist	1.6E-04	2.2E-02	7.0E-04	3.6E-03	1.4E-01	4.0E-01	2.07	1.57	-6.86	-6.86	2.80	2.10
LOC101927045	uncharacterized LOC101927045	7.0E-04	4.8E-02	7.2E-04	3.7E-03	5.0E-01	5.0E-01	1.73	1.86	-6.53	-6.53	1.48	2.11
CASC2	cancer susceptibility 2 (non-protein coding)	4.3E-04	3.7E-02	7.7E-04	3.9E-03	4.4E-01	4.9E-01	0.31	1.15	-7.83	-7.83	0.31	0.97
CYB5R2	cytochrome b5 reductase 2	8.5E-05	1.6E-02	8.0E-04	4.0E-03	4.5E-02	2.9E-01	3.09	2.75	-6.45	-6.45	2.09	1.43
C19orf70	chromosome 19 open reading frame 70	2.2E-05	5.9E-03	8.3E-04	4.0E-03	1.3E-01	4.0E-01	4.38	3.66	-5.59	-5.59	3.60	2.88
ZFYVE28	zinc finger FYVE-type containing 28	2.2E-04	2.7E-02	8.7E-04	4.1E-03	4.2E-01	4.9E-01	0.05	1.35	-8.00	-8.00	0.50	1.24
ZNF599	zinc finger protein 599	6.7E-04	4.7E-02	8.7E-04	4.1E-03	1.7E-01	4.1E-01	1.36	1.35	-7.09	-7.09	0.60	1.27
MKS1	Meckel syndrome, type 1	5.3E-04	4.2E-02	8.9E-04	4.1E-03	3.2E-01	4.6E-01	0.68	2.30	-6.80	-6.80	1.61	2.35
ESAM	endothelial cell adhesion molecule	1.2E-06	7.6E-04	9.6E-04	4.2E-03	4.6E-01	4.9E-01	1.90	1.38	6.05	5.69	1.64	1.70
ARMC6	armadillo repeat containing 6	3.3E-04	3.4E-02	9.6E-04	4.2E-03	2.9E-01	4.6E-01	2.22	1.29	-6.77	-6.77	2.55	1.76
DUOX1	dual oxidase 1	3.5E-04	3.4E-02	9.3E-04	4.2E-03	2.7E-01	4.6E-01	1.00	0.50	-7.96	-7.96	0.06	0.79
ENPEP	glutamyl aminopeptidase	3.9E-04	3.6E-02	1.2E-03	5.2E-03	3.1E-01	4.6E-01	0.76	2.80	4.60	4.40	2.39	2.33
B4GALT7	beta-1,4-galactosyltransferase 7	6.1E-04	4.5E-02	1.2E-03	5.2E-03	4.5E-01	4.9E-01	2.25	2.01	-6.24	-6.24	2.61	1.77
LEKR1	leucine, glutamate and lysine rich 1	6.3E-04	4.6E-02	1.4E-03	5.7E-03	1.9E-01	4.1E-01	0.95	0.52	-7.47	-7.47	0.85	1.77
XDH	xanthine dehydrogenase	1.4E-05	4.5E-03	1.5E-03	5.9E-03	7.4E-02	3.3E-01	1.08	0.80	-7.97	-7.97	2.81	1.70
EPAS1	endothelial PAS domain protein 1	1.1E-04	1.9E-02	1.5E-03	5.9E-03	1.9E-01	4.1E-01	4.63	4.71	6.82	6.66	4.65	4.48
RET	ret proto-oncogene	1.1E-04	1.9E-02	1.5E-03	5.9E-03	1.5E-01	4.0E-01	0.76	0.20	-8.21	-8.21	1.80	0.77
SLC44A3-AS1	SLC44A3 antisense RNA 1	5.9E-04	4.4E-02	1.6E-03	6.1E-03	6.3E-02	3.2E-01	2.74	2.76	-5.86	-5.86	2.10	1.25
TCEAL5	transcription elongation factor A like 5	1.7E-04	2.4E-02	1.6E-03	6.3E-03	1.6E-01	4.0E-01	3.49	3.25	-5.67	-5.67	3.19	2.23
LEO1	LEO1 homolog, Paf1/RNA polymerase II complex c	4.7E-07	3.5E-04	1.9E-03	7.1E-03	4.0E-02	2.7E-01	5.42	5.31	1.50	1.85	5.09	4.89
PLXNA3	plexin A3	2.3E-04	2.7E-02	2.1E-03	7.7E-03	3.4E-01	4.6E-01	0.85	1.69	4.13	3.95	1.34	1.61
TTC4	tetratricopeptide repeat domain 4	6.6E-04	4.6E-02	2.1E-03	7.7E-03	8.5E-02	3.6E-01	1.86	1.81	-6.71	-6.71	1.30	0.33
MOCOS	molybdenum cofactor sulfurase	2.6E-04	2.9E-02	2.1E-03	7.8E-03	5.0E-01	5.0E-01	0.76	2.38	-7.05	-7.05	2.13	1.00
LOC100506207	uncharacterized LOC100506207	1.3E-04	2.1E-02	2.2E-03	7.8E-03	6.9E-02	3.3E-01	2.51	2.08	-6.97	-6.97	0.42	1.47
SMIM22	small integral membrane protein 22	5.9E-04	4.4E-02	2.3E-03	8.0E-03	3.5E-01	4.6E-01	3.62	2.01	-5.61	-5.61	2.92	1.85
SDHAF3	succinate dehydrogenase complex assembly factor	1.3E-04	2.1E-02	2.4E-03	8.3E-03	2.9E-01	4.6E-01	3.29	1.78	-6.49	-6.49	2.50	1.34
RAB11FIP4	RAB11 family interacting protein 4	3.2E-06	1.5E-03	2.6E-03	8.9E-03	4.7E-01	4.9E-01	1.83	1.55	-4.61	-5.54	1.87	1.55
CGREF1	cell growth regulator with EF-hand domain 1	1.6E-04	2.3E-02	2.6E-03	8.9E-03	2.4E-01	4.4E-01	2.11	2.14	-6.88	-6.88	2.20	0.97
MRPL36	mitochondrial ribosomal protein L36	2.0E-04	2.6E-02	2.8E-03	9.2E-03	2.8E-01	4.6E-01	4.75	3.60	-4.75	-4.75	4.21	2.96
LINC01473	long intergenic non-protein coding RNA 1473	3.2E-04	3.4E-02	2.8E-03	9.2E-03	1.8E-01	4.1E-01	4.18	4.00	-4.72	-4.72	2.76	3.96
LINC01481	long intergenic non-protein coding RNA 1481	2.0E-04	2.6E-02	2.9E-03	9.3E-03	3.4E-01	4.6E-01	2.73	3.29	-5.87	-5.87	2.02	3.32
ROPN1B	rhophilin associated tail protein 1B	3.8E-04	3.5E-02	2.9E-03	9.3E-03	1.1E-01	4.0E-01	3.67	3.27	-2.47	-2.48	3.92	4.98
SNAPC2	small nuclear RNA activating complex polypeptide 2	6.0E-04	4.4E-02	3.0E-03	9.6E-03	3.8E-01	4.8E-01	0.69	3.37	-6.21	-6.21	2.11	0.91
MED21	mediator complex subunit 21	5.5E-05	1.2E-02	3.1E-03	9.9E-03	2.9E-01	4.6E-01	3.78	3.79	-0.75	-0.09	3.75	3.94
MANEA-AS1	NA	3.8E-04	3.5E-02	3.2E-03	9.9E-03	2.9E-01	4.6E-01	1.77	2.15	-6.72	-6.72	0.85	2.16
DBNDD1	dysbindin domain containing 1	4.7E-04	3.9E-02	3.3E-03	1.0E-02	3.1E-01	4.6E-01	2.30	0.92	-6.96	-6.96	1.71	0.41
SAXO2	stabilizer of axonemal microtubules 2	7.0E-04	4.8E-02	3.4E-03	1.0E-02	1.9E-01	4.1E-01	0.58	1.55	-7.40	-7.40	-0.43	0.82
FCER1G	Fc fragment of IgE receptor Ig	6.5E-04	4.6E-02	3.6E-03	1.1E-02	5.0E-01	5.0E-01	3.92	3.34	-4.69	-4.69	4.32	2.91
ARHGEF33	Rho guanine nucleotide exchange factor 33	6.6E-04	4.6E-02	3.7E-03	1.1E-02	1.3E-01	4.0E-01	0.50	1.50	-7.56	-7.56	-0.90	0.37
AOC3	amine oxidase, copper containing 3	1.6E-05	5.1E-03	3.8E-03	1.1E-02	1.4E-01	4.0E-01	3.51	1.41	5.63	5.21	1.22	0.54
SYN2	synapsin II	6.7E-04	4.7E-02	3.9E-03	1.1E-02	4.4E-01	4.9E-01	-2.14	1.97	-7.96	-7.96	-0.45	1.01
TMEM26	transmembrane protein 26	1.3E-04	2.1E-02	4.2E-03	1.2E-02	1.4E-01	4.0E-01	2.00	1.81	-2.48	-2.50	2.13	3.08
EEF1A2	eukaryotic translation elongation factor 1 alpha 2	4.0E-04	3.6E-02	4.6E-03	1.3E-02	4.0E-01	4.8E-01	2.12	1.86	-6.46	-6.46	3.08	1.40
CACNA1H	calcium voltage-gated channel subunit alpha1 H	2.0E-06	9.9E-04	4.7E-03	1.3E-02	6.6E-03	1.6E-01	0.07	-0.10	4.42	3.34	-2.38	-1.91
RAB3B	RAB3B, member RAS oncogene family	1.5E-04	2.2E-02	4.7E-03	1.3E-02	1.9E-01	4.1E-01	-2.62	0.18	-9.13	-9.13	-0.24	1.68
PADI2	peptidyl arginine deiminase 2	2.3E-05	6.2E-03	5.0E-03	1.4E-02	8.3E-03	1.7E-01	3.37	3.03	-0.18	0.73	5.29	4.93
CSMD1	CUB and Sushi multiple domains 1	3.9E-05	9.2E-03	5.5E-03	1.5E-02	3.7E-01	4.7E-01	0.31	-1.07	-9.29	-9.29	-0.93	1.05
GFRA1	GDNF family receptor alpha 1	3.3E-04	3.4E-02	5.7E-03	1.5E-02	7.5E-02	3.3E-01	6.12	4.73	3.53	3.53	3.88	3.81
EML5	echinoderm microtubule associated protein like 5	5.2E-04	4.1E-02	6.0E-03	1.6E-02	3.4E-01	4.6E-01	1.49	2.35	-2.34	-3.02	1.32	2.00
LINC01197	long intergenic non-protein coding RNA 1197	6.3E-07	4.3E-04	6.2E-03	1.6E-02	3.5E-01	4.6E-01	-1.06	-0.87	5.88	5.13	-0.60	-1.92
OXTR	oxytocin receptor	6.9E-05	1.4E-02	7.0E-03	1.8E-02	4.2E-01	4.9E-01	4.73	4.46	2.01	2.27	4.94	4.39
FLNC	filamin C	3.0E-04	3.2E-02	7.5E-03	1.9E-02	3.2E-01	4.6E-01	1.69	-0.67	3.61	3.45	0.27	-0.63
CES1	carboxylesterase 1	4.7E-04	3.9E-02	7.7E-03	2.0E-02	2.0E-01	4.2E-01	2.46	-0.23	-7.46	-7.46	0.27	-1.45
PDZK1	PDZ domain containing 1	2.6E-05	6.8E-03	8.0E-03	2.0E-02	4.5E-01	4.9E-01	3.76	0.72	-6.65	-6.65	3.71	1.36
HBB	hemoglobin subunit beta	3.3E-05	8.1E-03	8.2E-03	2.1E-02	2.8E-01	4.6E-01	1.50	5.38	7.74	6.94	1.52	2.62
RYR2	ryanodine receptor 2	1.3E-05	4.4E-03	8.3E-03	2.1E-02	3.1E-01	4.6E-01	0.38	-4.04	3.57	2.18	-3.48	-2.77
HS1BP3-IT1	HS1BP3 intronic transcript 1	1.3E-05	4.3E-03	8.6E-03	2.1E-02	9.6E-03	1.7E-01	-4.71	-4.71	5.60	4.77	-0.99	0.23
KCNAB1	potassium voltage-gated channel subfamily A memb	7.2E-04	4.8E-02	8.7E-03	2.1E-02	3.8E-02	2.7E-01	0.64	0.44	3.30	4.29	-0.30	-0.95
NKD1	naked cuticle homolog 1	3.5E-04	3.4E-02	8.9E-03	2.2E-02	4.5E-01	4.9E-01	-1.76	-1.57	2.94	2.32	-1.11	-2.08
NET1	neuroepithelial cell transforming 1	2.3E-04	2.7E-02	9.5E-03	2.3E-02	4.2E-02	2.8E-01	4.62	4.74	6.89	6.42	4.88	4.97
ACTC1	actin, alpha, cardiac muscle 1	1.2E-05	4.3E-03	1.0E-02	2.4E-02	1.1E-01	4.0E-01	-5.17	-7.34	3.83	1.77	-4.51	-4.26
CASQ2	calsequestrin 2	2.7E-04	3.0E-02	1.1E-02	2.5E-02	3.4E-01	4.6E-01	-2.22	-1.13	3.45	4.38	-1.37	-2.89
SNX24	sorting nexin 24	6.8E-04	4.7E-02	1.1E-02	2.5E-02	1.0E-01	3.8E-01	2.88	3.40	-3.54	-2.09	2.13	2.71
MEOX2	mesenchyme homeobox 2	1.2E-04	2.0E-02	1.1E-02	2.6E-02	3.7E-01	4.7E-01	2.55	3.25	5.68	5.78	3.14	2.22
CAV1	caveolin 1	1.4E-04	2.2E-02	1.1E-02	2.6E-02	1.4E-01	4.0E-01	4.27	4.02	6.26	6.29	3.94	3.11
ADCY2	adenylate cyclase 2	3.9E-04	3.6E-02	1.1E-02	2.6E-02	4.1E-02	2.7E-01	-4.65	-2.55	1.87	2.61	-0.15	-0.17
NGFR	nerve growth factor receptor	3.4E-04	3.4E-02	1.2E-02	2.7E-02	4.5E-01	4.9E-01	3.84	4.07	1.26	0.55	4.33	3.67
SPECC1	sperm antigen with calponin homology and coiled-c	4.5E-04	3.8E-02	1.2E-02	2.7E-02	1.5E-01	4.0E-01	2.51	1.79	4.12	4.62	2.76	2.57
EDNRA	endothelin receptor type A	2.0E-04	2.6E-02	1.2E-02	2.7E-02	1.2E-01	4.0E-01	2.42	3.99	5.95	4.85	1.98	1.84
TBX15	T-box 15	6.5E-05	1.3E-02	1.2E-02	2.8E-02	6.0E-03	1.6E-01	1.70	1.74	5.14	4.44	2.65	2.47
SUSD5	sushi domain containing 5	2.7E-05	6.9E-03	1.3E-02	2.8E-02	8.8E-02	3.7E-01	2.85	-0.36	5.50	3.71	-1.64	-3.02
TNS1	tensin 1	1.9E-04	2.5E-02	1.3E-02	2.9E-02	1.0E-01	3.8E-01	5.57	4.98	7.59	6.87	4.82	4.30
DSC2	desmocollin 2	5.4E-04	4.2E-02	1.5E-02	3.2E-02	1.3E-01	4.0E-01	5.18	4.66	2.64	3.36	5.73	5.23
SLFN13	schlafen family member 13	1.4E-06	8.6E-04	1.5E-02	3.2E-02	1.5E-01	4.0E-01	1.66	1.47	-8.53	-5.44	1.65	1.89

PLIN1	perilipin 1	6.3E-05	1.3E-02	1.5E-02	3.2E-02	5.8E-02	3.2E-01	1.72	1.98	4.41	5.72	0.80	1.32
CXADR	CXADR, Ig-like cell adhesion molecule	1.8E-04	2.4E-02	1.5E-02	3.2E-02	4.0E-01	4.8E-01	4.00	2.83	0.76	-0.03	3.54	2.91
PTGIS	prostaglandin I2 synthase	3.8E-06	1.7E-03	1.6E-02	3.3E-02	4.3E-01	4.9E-01	2.61	0.38	4.98	4.60	1.87	0.63
SPEG	SPEG complex locus	7.1E-04	4.8E-02	1.7E-02	3.5E-02	6.0E-03	1.6E-01	1.88	1.62	4.44	3.21	0.52	0.59
H2AFY2	H2A histone family member Y2	1.6E-04	2.2E-02	1.7E-02	3.5E-02	3.7E-01	4.7E-01	3.38	3.48	-1.60	-3.49	3.78	2.67
PLN	phospholamban	2.1E-10	6.4E-07	1.7E-02	3.5E-02	6.1E-02	3.2E-01	2.26	0.76	6.72	5.07	-1.19	-3.97
GABPB1-IT1	GABPB1 intronic transcript	8.3E-06	3.2E-03	1.8E-02	3.5E-02	4.7E-01	4.9E-01	2.50	2.16	-2.73	-4.93	1.90	2.85
MYOM1	myomesin 1	1.8E-05	5.4E-03	1.8E-02	3.6E-02	1.8E-02	2.0E-01	1.82	1.42	5.36	3.80	0.58	0.63
ANGPT2	angiopoietin 2	1.3E-04	2.1E-02	1.8E-02	3.6E-02	3.7E-02	2.7E-01	0.74	0.52	4.77	3.13	-0.25	-0.90
RHOBTB3	Rho related BTB domain containing 3	5.5E-04	4.2E-02	1.8E-02	3.6E-02	1.7E-02	2.0E-01	4.13	4.03	1.43	2.43	4.59	4.45
ANGPT1	angiopoietin 1	1.3E-04	2.1E-02	1.9E-02	3.6E-02	2.5E-01	4.4E-01	1.91	-0.07	5.23	3.53	0.03	0.16
LRRC32	leucine rich repeat containing 32	7.3E-04	4.9E-02	1.9E-02	3.6E-02	2.1E-02	2.2E-01	2.65	2.12	5.48	3.93	0.86	0.32
MREG	melanoregulin	7.3E-08	1.0E-04	1.9E-02	3.7E-02	9.4E-02	3.7E-01	3.98	4.21	0.26	-1.87	4.36	4.80
SLC9A5	solute carrier family 9 member A5	2.0E-06	9.9E-04	1.9E-02	3.7E-02	2.2E-01	4.4E-01	-7.42	-1.81	4.33	2.65	-2.53	-1.18
GCLC	glutamate-cysteine ligase catalytic subunit	3.3E-04	3.4E-02	2.0E-02	3.9E-02	3.9E-01	4.8E-01	3.36	3.04	-1.84	-0.14	3.00	3.27
GLRA3	glycine receptor alpha 3	3.6E-07	2.9E-04	2.1E-02	3.9E-02	3.1E-01	4.6E-01	2.32	2.71	-4.22	-3.33	4.80	1.92
FILIP1L	filamin A interacting protein 1 like	7.7E-06	3.1E-03	2.1E-02	3.9E-02	1.6E-01	4.0E-01	4.17	2.89	6.78	5.42	2.89	2.29
ST3GAL6	ST3 beta-galactoside alpha-2,3-sialyltransferase 6	1.1E-04	1.9E-02	2.1E-02	3.9E-02	3.3E-01	4.6E-01	1.47	1.93	-4.46	-7.56	1.38	2.78
NRG1	neuregulin 1	9.9E-05	1.8E-02	2.1E-02	3.9E-02	4.2E-01	4.9E-01	1.59	3.72	0.39	-0.65	2.58	2.25
TACO1	translational activator of cytochrome c oxidase I	4.4E-04	3.8E-02	2.1E-02	3.9E-02	2.9E-01	4.6E-01	3.66	2.71	-6.01	-2.92	3.06	2.65
JCHAIN	joining chain of multimeric IgA and IgM	1.9E-10	6.4E-07	2.3E-02	4.1E-02	3.4E-01	4.6E-01	8.83	8.61	5.35	4.65	9.08	7.74
LCP1	lymphocyte cytosolic protein 1	4.7E-04	3.9E-02	2.3E-02	4.2E-02	4.3E-01	4.9E-01	4.36	5.00	1.88	2.87	4.56	4.94
GRIA2	glutamate ionotropic receptor AMPA type subunit 2	2.0E-05	5.8E-03	2.4E-02	4.2E-02	1.3E-01	4.0E-01	1.44	2.79	-8.00	-4.91	0.26	1.34
ANXA3	annexin A3	7.2E-04	4.8E-02	2.4E-02	4.3E-02	4.5E-01	4.9E-01	5.66	5.38	2.64	3.59	5.74	5.22
EHBP1L1	EH domain binding protein 1 like 1	6.4E-04	4.6E-02	2.4E-02	4.3E-02	2.9E-01	4.6E-01	3.25	2.72	5.46	4.66	3.03	3.34
COL5A1	collagen type V alpha 1 chain	1.8E-04	2.4E-02	2.7E-02	4.7E-02	4.8E-01	4.9E-01	3.21	2.46	4.49	5.38	3.07	2.55
RCAN2	regulator of calcineurin 2	2.3E-04	2.7E-02	2.7E-02	4.7E-02	6.9E-02	3.3E-01	1.95	2.02	5.54	3.96	0.88	1.53
LINC00327	long intergenic non-protein coding RNA 327	1.4E-04	2.2E-02	2.7E-02	4.7E-02	1.8E-03	9.6E-02	-6.51	-6.51	1.01	3.62	-3.32	-2.91
LDB3	LIM domain binding 3	5.3E-04	4.2E-02	2.8E-02	4.8E-02	1.5E-02	1.8E-01	0.25	0.75	4.64	2.13	-1.56	-2.24
HBA2	hemoglobin subunit alpha 2	7.1E-06	2.9E-03	2.8E-02	4.8E-02	2.1E-01	4.3E-01	-4.73	-4.73	4.45	5.97	-1.00	-4.73
ST6GAL2	ST6 beta-galactoside alpha-2,6-sialyltransferase 2	4.3E-04	3.7E-02	2.8E-02	4.8E-02	1.3E-02	1.8E-01	-0.13	-0.42	3.68	2.59	0.99	0.74
EGF	epidermal growth factor	4.2E-04	3.7E-02	2.9E-02	4.8E-02	4.6E-01	4.9E-01	1.93	3.35	-2.42	-0.40	2.50	2.96
AEBP1	AE binding protein 1	1.4E-06	8.6E-04	2.9E-02	4.9E-02	1.3E-01	4.0E-01	5.46	4.56	8.37	6.80	4.46	3.61
IGFBP5	insulin like growth factor binding protein 5	1.7E-06	9.5E-04	3.0E-02	5.1E-02	2.7E-01	4.6E-01	6.73	5.55	9.35	8.04	6.04	5.10
CLEC5A	C-type lectin domain containing 5A	2.3E-05	6.2E-03	3.1E-02	5.1E-02	3.1E-02	2.5E-01	-7.27	-7.27	2.43	2.51	-1.14	-3.67
PDE3A	phosphodiesterase 3A	3.7E-07	2.9E-04	3.1E-02	5.1E-02	1.8E-01	4.1E-01	1.10	0.01	5.05	3.17	0.20	-1.43
GCOM1	GRINL1A complex locus 1	2.1E-05	5.9E-03	3.2E-02	5.1E-02	1.2E-04	3.2E-02	-8.21	-8.21	2.22	0.51	-1.78	-1.98
SCGB2A1	secretoglobin family 2A member 1	2.1E-05	5.9E-03	3.2E-02	5.1E-02	3.3E-01	4.6E-01	3.13	3.93	-4.51	-4.51	2.31	7.25
SCARA5	scavenger receptor class A member 5	1.2E-04	2.0E-02	3.2E-02	5.1E-02	1.3E-01	4.0E-01	3.67	4.63	1.66	0.41	3.33	3.45
AKAP6	A-kinase anchoring protein 6	1.4E-04	2.2E-02	3.1E-02	5.1E-02	4.7E-01	4.9E-01	1.41	1.42	4.47	3.21	1.27	1.54
ADAM33	ADAM metallopeptidase domain 33	1.7E-04	2.3E-02	3.2E-02	5.1E-02	5.4E-02	3.1E-01	3.85	3.24	4.75	6.49	1.15	2.30
SORBS2	sorbin and SH3 domain containing 2	2.1E-04	2.6E-02	3.1E-02	5.1E-02	3.6E-01	4.7E-01	4.25	3.38	6.29	5.35	4.09	3.91
ANKS1B	ankyrin repeat and sterile alpha motif domain contai	2.9E-04	3.2E-02	3.2E-02	5.1E-02	3.6E-01	4.7E-01	2.04	3.06	-2.17	-0.31	2.11	2.54
KCNC2	potassium voltage-gated channel subfamily C meml	3.2E-07	2.9E-04	3.3E-02	5.1E-02	2.2E-01	4.4E-01	3.25	1.86	-4.03	-8.05	1.17	2.26
CLGN	calmegin	1.5E-04	2.2E-02	3.3E-02	5.2E-02	1.5E-01	4.0E-01	1.07	2.41	-2.93	-6.95	2.37	4.39
SYNPO	synaptopodin	3.8E-04	3.5E-02	3.4E-02	5.2E-02	1.7E-01	4.1E-01	3.13	2.35	5.07	4.34	2.44	0.96
GRPR	gastrin releasing peptide receptor	1.2E-07	1.4E-04	3.5E-02	5.3E-02	1.7E-01	4.1E-01	4.46	3.20	-6.87	-3.78	3.31	0.67
MYOC	myocilin	6.0E-05	1.3E-02	3.5E-02	5.3E-02	6.1E-02	3.2E-01	-6.48	-6.48	2.18	3.62	-1.41	-4.24
HMGCS2	3-hydroxy-3-methylglutaryl-CoA synthase 2	1.0E-08	2.5E-05	3.5E-02	5.3E-02	3.9E-01	4.8E-01	4.84	2.47	-2.18	-6.75	3.37	4.84
ANK1	ankyrin 1	1.5E-04	2.2E-02	3.5E-02	5.3E-02	1.5E-01	4.0E-01	-8.67	-5.13	0.92	1.46	-5.18	-2.43
FYB2	FYN binding protein 2	2.7E-04	3.0E-02	3.6E-02	5.4E-02	4.1E-01	4.9E-01	1.19	2.77	-3.22	-7.24	1.56	2.91
DUBR	DPPA2 upstream binding RNA	5.0E-04	4.1E-02	3.7E-02	5.5E-02	4.6E-01	4.9E-01	0.05	3.36	5.26	3.89	1.52	2.23
TM2D3	TM2 domain containing 3	3.5E-04	3.4E-02	3.7E-02	5.5E-02	3.3E-02	2.6E-01	4.23	4.70	-0.44	1.35	3.50	3.63
SLFN11	schlafen family member 11	3.6E-04	3.5E-02	3.7E-02	5.5E-02	3.1E-01	4.6E-01	0.92	1.72	3.72	4.75	2.22	1.18
MCAM	melanoma cell adhesion molecule	3.9E-06	1.7E-03	3.8E-02	5.6E-02	2.5E-01	4.4E-01	4.80	3.81	7.49	6.11	4.27	3.04
PNLDC1	PARN like, ribonuclease domain containing 1	1.1E-04	1.9E-02	3.9E-02	5.7E-02	1.2E-02	1.7E-01	-6.41	-6.41	2.11	3.26	-1.74	0.03
CHMP4C	charged multivesicular body protein 4C	2.3E-04	2.7E-02	3.9E-02	5.7E-02	1.9E-01	4.1E-01	4.15	3.88	-2.31	0.67	4.34	4.09
TFPI2	tissue factor pathway inhibitor 2	2.5E-08	4.3E-05	4.0E-02	5.7E-02	2.9E-01	4.6E-01	4.55	-0.07	-3.63	-2.74	6.60	2.16
ADORA1	adenosine A1 receptor	5.5E-04	4.2E-02	4.0E-02	5.7E-02	1.4E-01	4.0E-01	2.70	1.89	-2.42	-7.00	2.93	2.88
MIR17HG	miR-17-92a-1 cluster host gene	2.1E-04	2.6E-02	4.0E-02	5.8E-02	1.8E-01	4.1E-01	2.04	1.67	-3.20	-7.78	2.05	2.08
CSE1L	chromosome segregation 1 like	3.8E-05	9.0E-03	4.1E-02	5.8E-02	3.3E-01	4.6E-01	4.38	4.88	0.98	2.70	5.19	4.51
PDGFRB	platelet derived growth factor receptor beta	2.6E-04	3.0E-02	4.1E-02	5.8E-02	4.5E-01	4.9E-01	4.63	4.75	7.03	6.12	5.02	4.25
KANK2	KN motif and ankyrin repeat domains 2	7.2E-04	4.8E-02	4.1E-02	5.8E-02	9.5E-02	3.7E-01	4.68	4.33	6.88	5.54	3.92	2.81
ACAN	aggrecan	6.7E-22	1.2E-17	4.2E-02	5.9E-02	6.6E-03	1.6E-01	-8.59	-8.59	5.40	-0.01	-5.77	-6.36
NEBL	nebulette	6.2E-04	4.5E-02	4.4E-02	6.1E-02	7.0E-02	3.3E-01	3.65	4.07	0.94	2.60	4.31	4.55
BCL2L1	BCL2 like 1	7.4E-04	4.9E-02	4.4E-02	6.1E-02	3.3E-01	4.6E-01	3.78	3.60	1.25	-0.95	3.71	3.55
DPY19L2P4	DPY19L2 pseudogene 4	4.6E-05	1.0E-02	4.4E-02	6.1E-02	2.4E-02	2.3E-01	4.44	3.07	-3.36	-6.46	0.57	-0.30
TJP3	tight junction protein 3	1.2E-05	4.3E-03	4.6E-02	6.2E-02	4.9E-01	5.0E-01	3.55	2.28	-7.19	-2.23	3.20	2.67
MARCKSL1	MARCKS like 1	2.0E-04	2.6E-02	4.6E-02	6.2E-02	1.6E-01	4.0E-01	6.07	5.21	3.97	1.82	6.58	6.04
ZNF79	zinc finger protein 79	3.7E-04	3.5E-02	4.6E-02	6.2E-02	2.8E-01	4.6E-01	3.35	2.51	-6.67	-2.11	2.54	2.73
LAMA5	laminin subunit alpha 5	6.7E-04	4.7E-02	4.6E-02	6.2E-02	1.7E-01	4.1E-01	2.72	2.98	5.33	4.03	2.73	2.61
C1QTNF2	C1q and TNF related 2	7.1E-04	4.8E-02	4.5E-02	6.2E-02	1.4E-01	4.0E-01	-3.72	-6.71	1.31	3.45	-1.33	-3.63
FHL5	four and a half LIM domains 5	9.2E-06	3.5E-03	4.8E-02	6.4E-02	1.7E-01	4.1E-01	3.56	0.57	6.79	3.50	0.44	-0.08
PRPF19	pre-mRNA processing factor 19	1.7E-04	2.3E-02	4.9E-02	6.5E-02	4.7E-01	4.9E-01	4.79	4.55	0.43	2.50	4.94	4.34
SORD	sorbitol dehydrogenase	7.6E-04	5.0E-02	5.0E-02	6.6E-02	7.7E-03	1.7E-01	2.20	1.73	-1.44	-6.99	3.83	3.92
PPP1R12B	protein phosphatase 1 regulatory subunit 12B	3.6E-04	3.5E-02	5.0E-02	6.6E-02	3.7E-01	4.7E-01	4.03	3.66	6.44	5.13	3.41	4.00
COL1A2	collagen type I alpha 2 chain	2.3E-04	2.7E-02	5.1E-02	6.7E-02	3.4E-01	4.6E-01	5.76	4.89	7.18	7.33	6.20	5.10
METTL22	methyltransferase like 22	6.9E-04	4.8E-02	5.1E-02	6.7E-02	2.6E-02	2.4E-01	3.41	3.53	-1.55	-6.13	2.59	2.91
CIR1	corepressor interacting with RBPJ, 1	4.4E-05	1.0E-02	5.2E-02	6.8E-02	7.1E-02	3.3E-01	4.67	5.17	2.98	0.09	5.47	5.88
METTL14	methyltransferase like 14	3.4E-04	3.4E-02	5.4E-02	6.9E-02	4.6E-01	4.9E-01	4.02	4.58	2.54	0.61	4.12	4.57
SPEF2	sperm flagellar 2	3.2E-05	7.9E-03	5.6E-02	7.2E-02	3.4E-01	4.6E-01	2.53	3.09	-0.11	-2.73	1.90	3.08
COL5A2	collagen type V alpha 2 chain	6.2E-04	4.5E-02	5.7E-02	7.2E-02	3.1E-01	4.6E-01	2.80	3.23	5.07	4.84	3.94	2.80
FOXL1	forkhead box L1	2.7E-04	3.0E-02	5.7E-02	7.3E-02	5.5E-03	1.6E-01	-7.12	-7.12	2.68	0.12	-1.81	-2.83
LINC00504	long intergenic non-protein coding RNA 504	3.7E-04	3.5E-02	5.8E-02	7.3E-02	4.9E-01	5.0E-01	1.40	2.25	-2.70	-7.67	1.02	2.57
IGFBP7	insulin like growth factor binding protein 7	3.0E-06	1.5E-03	5.9E-02	7.4E-02	2.4E-01	4.4E-01	6.11	5.46	9.25	7.24	5.76	4.78
CCDC102B	coiled-coil domain containing 102B	5.2E-06	2.2E-03	5.9E-02	7.4E-02	2.2E-01	4.4E-01	-4.83	1.57	5.43	3.24	1.11	1.62
MPZL3	myelin protein zero like 3	4.0E-04	3.6E-02	5.9E-02	7.4E-02	4.4E-01	4.9E-01	3.19	0.64	-7.45	-2.18	2.01	2.29
CSPG4	chondroitin sulfate proteoglycan 4	1.8E-08	3.5E-05	6.0E-02	7.4E-02	1.9E-01	4.1E-01	1.04	0.87	5.76	2.95	0.90	0.18
HMGB3	high mobility group box 3	4.3E-04	3.7E-02	6.0E-02	7.5E-02	3.9E-01	4.8E-01	3.74	1.30	-3.40	-0.11	3.72	2.21
IGLL5	immunoglobulin lambda like polypeptide 5	3.4E-14	2.9E-10	6.1E-02	7.5E-02	4.6E-01	4.9E-01	7.32	6.06	-1.51	2.67	7.52	5.57
YARS2	tyrosyl-tRNA synthetase 2	5.7E-04	4.3E-02	6.3E-02	7.7E-02	2.0E-01	4.2E-01	2.84	3.45	-1.28	-6.57	2.72	2.90
ANKRD36BP2	ankyrin repeat domain 36B pseudogene 2	7.4E-04	4.9E-02	6.3E-02	7.7E-02	1.4E-01	4.0E-01	4.14	4.61	-1.67	1.53	4.11	3.88
RHPN2	rhophilin Rho GTPase binding protein 2	5.0E-07	3.6E-04	6.4E-02	7.7E-02	4.3E-01	4.9E-01	4.50	3.24	-4.16	0.29	3.44	4.02
INHBA	inhibin beta A subunit	1.6E-10	6.4E-07	6.4E-02	7.8E-02	3.9E-01	4.8E-01	-0.36	-0.88	5.86	2.25	-0.26	-0.75
LRP12	LDL receptor related protein 12	5.7E-04	4.3E-02	6.6E-02	8.0E-02	9.0E-02	3.7E-01	2.22	2.18	-1.74	-7.50	2.34	2.61
EBF2	early B cell factor 2	1.8E-04	2.4E-02	7.0E-02	8.3E-02	3.8E-01	4.8E-01	-0.64	0.11	4.65	2.13	0.64	-0.66
COL1A1	collagen type I alpha 1 chain	6.8E-08	1.0E-04	7.2E-02	8.5E-02	2.7E-01	4.6E-01	5.45	3.89	7.22	8.04	6.31	4.71
MIR650	microRNA 650	5.6E-05	1.2E-02	7.9E-02	9.3E-02	1.7E-01	4.1E-01	8.67	7.10	-2.07	1.93	7.30	4.02
MYL9	myosin light chain 9	2.3E-04	2.7E-02	8.0E-02	9.4E-02	2.4E-01	4.4E-01	7.30	6.64	9.65	7.88	7.00	5.68
PRKG1	protein kinase, cGMP-dependent, type I	9.5E-05	1.7E-02	8.4E-02	9.9E-02	4.2E-01	4.9E-01	1.56	3.09	5.69	3.42	2.15	2.15
TNN	tenascin N	1.4E-04	2.2E-02	8.6E-02	1.0E-01	3.7E-01	4.7E-01	3.54	3.42	1.40	-2.51	3.84	3.32
COL18A1	collagen type XVIII alpha 1 chain	1.4E-04	2.2E-02	8.6E-02	1.0E-01	2.4E-01	4.4E-01	4.90	4.12	7.37	5.36	4.52	3.26
DCD	dermcidin	2.2E-10	6.4E-07	9.1E-02	1.1E-01	4.8E-01	4.9E-01	8.32	-4.95	-4.95	-4.95	4.24	-1.88
BGN	biglycan	1.6E-06	9.3E-04	9.6E-02	1.1E-01	1.4E-01	4.0E-01	4.14	3.61	8.01	4.60	3.45	1.90
RRM1	ribonucleotide reductase catalytic subunit M1	2.5E-04	2.9E-02	9.6E-02	1.1E-01	2.4E-01	4.4E-01	4.22	3.20	-2.00	2.21	4.55	3.90
EDIL3	EGF like repeats and discoidin domains 3	3.3E-04	3.4E-02	1.0E-01	1.2E-01	3.4E-01	4.6E-01	2.69	2.96	5.94	3.74	3.08	2.07
LTBP1	latent transforming growth factor beta binding protei	3.6E-07	2.9E-04	1.0E-01	1.2E-01	3.4E-01	4.6E-01	2.80	1.92	6.96	3.57	2.22	2.06
SLC9A3R1	SLC9A3 regulator 1	3.7E-04	3.5E-02	1.0E-01	1.2E-01	2.8E-01	4.6E-01	6.06	5.55	2.17	4.61	5.66	5.59
PLS3	plastin 3	3.9E-05	9.2E-03	1.1E-01	1.2E-01	1.3E-01	4.0E-01	4.27	4.10	7.33	5.37	4.78	4.33
CLCA2	chloride channel accessory 2	1.1E-07	1.4E-04	1.1E-01	1.2E-01	1.3E-01	4.0E-01	4.12	4.97	1.70	-1.43	2.46	3.98
CTGF	connective tissue growth factor	1.5E-07	1.5E-04	1.1E-01	1.2E-01	3.0E-01	4.6E-01	4.34	2.39	8.36	4.34	3.19	2.21
CA2	carbonic anhydrase 2	7.9E-05	1.5E-02	1.2E-01	1.3E-01	4.4E-01	4.9E-01	7.04	2.31	1.99	-6.19	6.53	3.79
RASGRF2	Ras protein specific guanine nucleotide releasing fa	5.0E-04	4.1E-02	1.2E-01	1.3E-01	2.8E-01	4.6E-01	-1.35	0.88	1.59	4.19	0.10	1.08
PCNA	proliferating cell nuclear antigen	2.3E-04	2.7E-02	1.2E-01	1.4E-01	3.9E-01	4.8E-01	4.61	4.87	1.96	-5.98	5.15	3.91
MUCL1	mucin like 1	5.8E-05	1.2E-02	1.3E-01	1.4E-01	4.2E-01	4.9E-01	11.75	8.30	8.07	4.56	10.66	8.48
ZNF812P	zinc finger protein 812, pseudogene	6.6E-04	4.6E-02	1.3E-01	1.4E-01	2.2E-02	2.2E-01	-4.89	-7.06	-0.03	3.12	-1.10	-0.75
TSPAN13	tetraspanin 13	2.6E-05	6.8E-03	1.3E-01	1.5E-01	5.6E-02	3.2E-01	5.70	5.31	-0.83	3.75	5.00	4.86
SPARC	secreted protein acidic and cysteine rich	6.6E-04	4.6E-02	1.4E-01	1.5E-01	4.5E-01	4.9E-01	6.01	5.96	8.64	6.58	6.41	5.42
FN1	fibronectin 1	1.3E-07	1.4E-04	1.4E-01	1.5E-01	2.8E-01	4.6E-01	5.20	4.18	9.07	5.14	4.90	2.88
PLAT	plasminogen activator, tissue type	2.2E-04	2.7E-02	1.4E-01	1.6E-01	4.0E-01	4.8E-01	5.57	5.62	2.15	4.64	6.06	4.75
CHMP2A	charged multivesicular body protein 2A	4.7E-04	3.9E-02	1.5E-01	1.6E-01	4.4E-01	4.9E-01	7.22	5.56	2.39	5.52	6.72	5.73
COL4A1	collagen type IV alpha 1 chain	1.1E-05	4.2E-03	1.5E-01	1.6E-01	2.3E-01	4.4E-01	3.87	3.74	7.38	4.65	4.41	3.78
RGS5	regulator of G protein signaling 5	1.7E-05	5.3E-03	1.6E-01	1.7E-01	5.0E-02	3.1E-01	4.50	4.71	8.68	4.80	4.09	4.29
TAGLN	transgelin	4.2E-04	3.7E-02	1.7E-01	1.8E-01	4.9E-01	5.0E-01	6.33	5.74	8.59	6.69	6.93	5.05
SERPINA1	serpin family A member 1	2.0E-05	5.8E-03	1.8E-01	1.9E-01	3.8E-01	4.8E-01	4.27	-1.82	-8.33	1.10	4.86	0.17
NCOA3	nuclear receptor coactivator 3	1.2E-07	1.4E-04	2.0E-01	2.1E-01	4.0E-01	4.8E-01	3.71	3.72	8.96	3.84	3.65	3.83
SNORA81	small nucleolar RNA, H/ACA box 81	5.9E-04	4.4E-02	2.1E-01	2.2E-01	5.3E-02	3.1E-01	4.78	6.12	8.09	7.66	7.89	9.94
PI15	peptidase inhibitor 15	3.1E-05	7.8E-03	2.2E-01	2.3E-01	2.1E-01	4.3E-01	7.19	5.43	3.86	3.97	5.99	3.88
COL3A1	collagen type III alpha 1 chain	2.9E-04	3.2E-02	2.3E-01	2.4E-01	5.3E-02	3.1E-01	5.29	5.22	7.38	6.88	7.11	6.14

ITGA1	integrin subunit alpha 1	5.5E-04	4.2E-02	2.6E-01	2.7E-01	1.4E-01	4.0E-01	3.53	3.88	6.74	4.05	4.68	3.92
ALB	albumin	5.7E-04	4.3E-02	2.6E-01	2.7E-01	4.3E-01	4.9E-01	3.72	1.52	2.79	7.53	5.51	-1.92
TNC	tenascin C	5.4E-05	1.2E-02	2.6E-01	2.7E-01	3.5E-01	4.6E-01	4.45	3.01	6.77	4.35	5.41	3.24
DIO2	iodothyronine deiodinase 2	6.1E-06	2.5E-03	2.8E-01	2.9E-01	1.2E-01	4.0E-01	-0.53	0.25	3.45	0.73	6.48	1.54
VCAN	versican	1.8E-06	9.5E-04	2.9E-01	3.0E-01	2.2E-01	4.4E-01	3.47	3.48	7.87	3.26	4.63	3.45
LINC02344	long intergenic non-protein coding RNA 2344	3.4E-04	3.4E-02	4.1E-01	4.1E-01	1.1E-03	9.6E-02	-4.35	-4.35	-4.35	6.48	2.18	2.84
CP	ceruloplasmin	1.9E-07	1.8E-04	4.5E-01	4.5E-01	3.0E-01	4.6E-01	4.55	-1.60	5.77	2.71	6.04	1.62
TEX41	testis expressed 41 (non-protein coding)	4.2E-04	3.7E-02	4.7E-01	4.7E-01	8.0E-04	9.6E-02	-7.71	-7.71	3.21	-7.71	-2.56	-2.95
LINC01268	long intergenic non-protein coding RNA 1268	2.4E-04	2.8E-02	4.8E-01	4.9E-01	1.3E-02	1.7E-01	-6.91	-6.91	4.24	-6.91	-2.03	-0.14
ECEL1	endothelin converting enzyme like 1	4.2E-04	3.7E-02	4.9E-01	4.9E-01	9.4E-03	1.7E-01	-6.98	-6.98	3.54	-3.90	0.60	-1.25

Supplementary Table 2: Demographics of the study cohort: Susceptible and healthy control (HC) breast tissues were analyzed by immunohistochemistry

	Age at Donation_range	BMI* range	Racial background[#]	Menopausal status[**]	BC*** History	Family History of BC	BC Type	Collection year	Year of diagnosis	ER	PR	HER2
Susceptible 9	56-60	>35	White	post	No	Yes (sisiter)	Invasive	2014	2014	+	+	-
Susceptible 8	51-55	20-25	White	pre	No	No	DCIS	2011	2012	+	+	+
Susceptible 38	51-55	>35	AfrAm	post	No	No	DCIS	2012	2017	N/A	N/A	N/A
Susceptible 37	51-55	31-35	AfrAm	post	No	Yes (mother)	N/A	2010	2013	N/A	N/A	N/A
Susceptible 36	36-40	31-35	AfrAm	pre	No	NA	DCIS, Invasive	2012	2014	-	-	-
Susceptible 35	61-65	26-30	AfrAm	post	No	No	N/A	2012	2012	N/A	N/A	N/A
Susceptible 34	36-40	26-30	White	pre	No	No	DCIS	2014	2018	+	+	-
Susceptible 33	61-65	31-35	White	post	No	Yes (sister)	Invasive	2012	2017	+	+	-
Susceptible 32	56-60	31-35	White	post	No	No	DCIS	2012	2017	+	+	-
Susceptible 31	51-55	20-25	White	post	No	No	DCIS	2011	2012	+	-	-
Susceptible 30	41-45	26-30	AfrAm	pre	No	Yes (mother)	Invasive	2012	2017	+	+	-
Susceptible 29	30-35	20-25	White	pre	No	No	Invasive, DCIS, IDC	2010	2017	-	-	+
Susceptible 28	66-70	26-30	White	post	No	Yes (mother, sister)	Invasive, DCIS, IDC	2010	2017	+	N/A	+
Susceptible 27	66-70	26-30	White	post	No	Yes (sister)	DCIS	2014	2016	+	-	-
Susceptible 26	61-65	20-25	White	post	No	Yes (mother)	DCIS	2014	2017	N/A	N/A	N/A
Susceptible 25	56-60	26-30	White	post	No	No	Invasive	2015	2015	-	-	+
Susceptible 24	51-55	>35	White	post	No	No	DCIS	2011	2017	N/A	N/A	N/A
Susceptible 23	41-45	26-30	White	post	No	No	Invasive	2010	2017	-	-	-
Susceptible 22	51-55	31-35	White	post	No	Yes (mother)	DCIS	2011	2016	+	+	-
Susceptible 21	61-65	26-30	White	post	No	No	Invasive, DCIS, IDC	2010	2016	-	-	-
Susceptible 20	46-50	31-35	White	pre	No	No	DCIS	2009	2016	-	-	-
Susceptible 19	46-50	31-35	White	pre	No	No	Invasive	2013	2017	-	-	+
Susceptible 18	66-70	<20	White	post	No	Yes (mother)	DCIS	2014	2015	+	-	-
Susceptible 17	56-60	26-30	White	post	No	No	Invasive	2011	2016	+	+	+
Susceptible 16	61-65	31-35	White	post	No	Yes (Mother)	Invasive	2010	2015	+	-	-
Susceptible 15	36-40	31-35	White	pre	No	No	Invasive	2011	2015	+	+	-
Susceptible 14	56-60	26-30	White	post	No	Yes (mother)	Invasive/ DCIS	2012	2016	+	+	-
Susceptible 13	56-60	31-35	White	post	No	No	DCIS	2011	2014	+	+	-
Susceptible 12	56-60	26-30	White	post	No	No	DCIS	2014	2014	-	-	+
Susceptible 11	>70	26-30	White	post	No	No	Invasive	2009	2012	-	-	-
Susceptible 10	56-60	26-30	White	post	No	No	DCIS	2012	2012	+	-	-
HC9	56-60	31-35	White	pre	No	Yes (Mother)		2011				
HC8	51-55	26-30	White	post	No	No		2012				
HC38	51-55	31-35	AfrAm	post	No	No		2012				
HC37	51-55	31-35	AfrAm	post	No	Yes (mother)		2012				
HC36	36-40	31-35	AfrAm	pre	No	No		2012				
HC35	61-65	26-30	AfrAm	post	No	Yes (mother)		2016				
HC34	36-40	20-25	White	pre	No	No		2016				
HC33	61-65	31-35	White	post	No	No		2013				
HC32	51-55	31-35	White	post	No	No		2013				
HC31	51-55	26-30	White	post	No	No		2012				
HC30	41-45	26-30	AfrAm	pre	No	No		2012				
HC29	30-35	20-25	White	pre	No	No		2012				
HC28	66-70	26-30	White	post	No	No		2011				
HC27	66-70	26-30	White	post	No	No		2013				
HC26	66-70	20-25	White	post	No	No		2013				
HC25	51-55	26-30	White	post	No	No		2014				
HC24	51-55	31-35	White	post	No	No		2011				
HC23	41-45	31-35	White	post	No	No		2010				
HC22	51-55	31-35	White	post	No	No		2010				
HC21	61-65	26-30	White	post	No	Yes (mother, sister)		2016				
HC20	46-50	31-35	White	pre	No	Yes (sisters)		2012				
HC19	46-50	31-35	White	pre	No	No		2014				
HC18	66-70	20-25	White	post	No	Yes (mother)		2014				
HC17	56-60	26-30	White	post	No	No		2010				
HC16	61-65	26-30	White	post	No	No		2010				
HC15	36-40	>35	White	pre	No	No		2014				
HC14	56-60	26-30	White	post	No	Yes (sister)		2013				
HC13	56-60	>35	White	post	No	Yes (Mother)		2010				
HC12	56-60	26-30	White	post	No	No		2009				
HC11	>70	26-30	White	post	No	No		2012				
HC10	56-60	26-30	White	post	No	No		2014				

*BMI: Body Mass Index;#: White: Caucasian, AfrAm: African American;**pre: premenopausal, post: postmenopausal; ** BC: Breast Cancer; [#]N/A: Not Available; DCIS: Ductal Carcinoma *In Situ;* IDC: Invasive Ductal Carcinoma; ILC:Infiltrating Lobular Carcinoma.

Supplementary Table 3: List of genes differentially expressed in the microdissected breast epithelium, stroma and adipose tissue between healthy women at either luteal (italic) or follicular (underlined) phase of the menstrual cycle.

Breast compartment	Reason for removal	Gene Symbol	Gene Name	*p* value*	Raw counts															
					K105450	K104334	K102687	K105891	K106169	K107267	K105969	K104374	K105568	*K105179*	*K106154*	*K106715*	*K106836*	*K105868*	*K105805*	*K100899*
Microdissected TDLU	Change between Menstrual Phases	HLA-H	major histocompatibility complex, class I, H (pseudogene)	1.8E-03	239	158	158	339	270	194	75	264	111	939	461	275	519	467	421	286
Microdissected TDLU	Change between Menstrual Phases	FECH	ferrochelatase	1.4E-02	396	370	284	399	293	253	308	350	343	472	376	343	396	360	496	363
Microdissected TDLU	Change between Menstrual Phases	CCDC184	coiled-coil domain containing 184	5.8E-03	16	15	13	21	10	17	11	22	9	30	32	14	16	51	24	27
Microdissected TDLU	Change between Menstrual Phases	RHOBTB3	Rho related BTB domain containing 3	1.2E-02	1358	797	729	690	1126	650	964	1072	760	2427	1082	1241	958	1104	2264	1178
Microdissected TDLU	Change between Menstrual Phases	MFHAS1	malignant fibrous histiocytoma amplified sequence 1	7.2E-03	213	166	161	192	107	117	142	195	109	280	205	178	231	187	159	268
Microdissected TDLU	Change between Menstrual Phases	CYP4F22	cytochrome P450 family 4 subfamily F member 22	8.8E-03	130	29	507	118	179	139	49	143	22	572	357	367	213	312	345	175
Microdissected TDLU	Change between Menstrual Phases	ZNF233	zinc finger protein 233	4.2E-02	71	73	75	39	66	53	41	94	52	64	71	28	43	29	48	43
Microdissected TDLU	Change between Menstrual Phases	CBFA2T3	CBFA2/RUNX1 translocation partner 3	2.3E-03	136	108	155	135	121	146	91	105	91	221	133	132	139	186	191	193
Microdissected TDLU	Change between Menstrual Phases	SCGB1B2P	secretoglobin family 1B member 2, pseudogene	1.4E-02	4	3	25	12	11	4	8	16	12	45	14	21	4	36	96	140
Microdissected TDLU	Change between Menstrual Phases	AKR1C3	aldo-keto reductase family 1 member C3	3.1E-02	181	111	220	93	118	143	132	106	237	108	135	73	182	68	82	31
Microdissected TDLU	Change between Menstrual Phases	ATAD2	ATPase family, AAA domain containing 2	9.8E-03	972	688	851	749	681	335	729	947	840	1414	894	1642	870	1059	964	787
Microdissected TDLU	Change between Menstrual Phases	HCAR2	hydroxycarboxylic acid receptor 2	1.1E-02	18	10	22	6	17	6	12	13	4	26	11	17	20	17	19	31
Microdissected TDLU	Change between Menstrual Phases	MIR103A1	microRNA 103a-1	2.7E-02	14	6	13	7	10	5	11	7	15	12	7	8	2	0	8	2
Microdissected TDLU	Change between Menstrual Phases	GLA	galactosidase alpha	4.6E-02	182	521	409	347	218	260	143	149	111	233	236	144	85	127	150	134
Microdissected TDLU	Change between Menstrual Phases	KLHL31	kelch like family member 31	1.8E-02	119	160	166	45	99	42	192	78	178	57	47	89	60	70	90	64
Microdissected TDLU	Change between Menstrual Phases	KCNIP3	potassium voltage-gated channel interacting protein 3	6.5E-03	56	61	24	69	55	71	37	57	21	68	84	83	56	85	67	66
Microdissected TDLU	Change between Menstrual Phases	LOC729970	hCG2028352-like	1.3E-02	35	26	35	30	5	29	13	32	14	53	38	29	32	33	36	35
Microdissected TDLU	Change between Menstrual Phases	APOBEC3F	apolipoprotein B mRNA editing enzyme catalytic subunit 3F	2.5E-02	163	182	158	228	136	155	143	205	115	238	180	127	228	234	203	226
Microdissected TDLU	Change between Menstrual Phases	CYP4A11	cytochrome P450 family 4 subfamily A member 11	2.7E-02	3	0	3	1	1	4	4	1	0	9	7	2	1	30	1	15
Microdissected TDLU	Change between Menstrual Phases	FZD2	frizzled class receptor 2	3.2E-02	82	72	23	46	59	41	59	74	44	103	91	49	64	94	59	67
Microdissected TDLU	Change between Menstrual Phases	C14orf180	chromosome 14 open reading frame 180	3.4E-02	7	3	7	0	0	8	2	1	1	0	2	0	1	0	2	0
Microdissected TDLU	Change between Menstrual Phases	BMP8A	bone morphogenetic protein 8a	3.6E-02	19	11	15	6	2	11	4	9	6	30	11	12	12	13	12	16
Microdissected TDLU	Change between Menstrual Phases	AFF3	AF4/FMR2 family member 3	3.8E-02	2584	1914	4034	1540	2343	1059	1417	2018	1216	6439	3969	1917	1513	2027	3445	3566
Microdissected TDLU	Change between Menstrual Phases	KCNK2	potassium two pore domain channel subfamily K member 2	4.0E-02	497	324	175	634	90	553	184	136	270	62	299	162	215	163	174	101
Microdissected TDLU	Change between Menstrual Phases	IGFN1	immunoglobulin-like and fibronectin type III domain containing 1	4.2E-02	11	2	3	1	2	2	3	0	2	3	5	12	3	2	9	9
Microdissected TDLU	Change between Menstrual Phases	KDELR3	KDEL endoplasmic reticulum protein retention receptor 3	4.4E-02	169	86	64	87	140	55	59	87	41	284	87	113	113	82	228	100
Microdissected TDLU	Change between Menstrual Phases	NFKBIZ	NFKB inhibitor zeta	4.5E-02	2155	1769	996	1598	2009	1936	2141	1165	2662	881	1904	626	1470	1554	2032	912
Microdissected TDLU	Pardo et al, 2014	ABCC8	ATP binding cassette subfamily C member 8	8.4E-02	80	101	162	99	107	48	44	90	55	316	271	62	65	145	79	62
Microdissected TDLU	Pardo et al, 2014	EREG	epiregulin	4.5E-01	19	55	129	43	59	1	13	11	42	153	132	12	0	10	3	4
Microdissected TDLU	Pardo et al, 2014	ESRRB	estrogen related receptor beta	3.0E-01	4	44	0	0	3	19	17	72	16	6	31	17	8	21	7	10
Microdissected TDLU	Pardo et al, 2014	HMGCS2	3-hydroxy-3-methylglutaryl-CoA synthase 2	4.6E-01	285	6	3984	550	52	87	18	17	23	456	152	226	103	85	747	1731
Microdissected TDLU	Pardo et al, 2014	FASN	fatty acid synthase	1.6E-01	9686	8730	26884	2389	6336	4453	2002	3686	2850	16435	10866	9830	7072	32158	3509	4660
Microdissected TDLU	Pardo et al, 2014	INPP5J	inositol polyphosphate-5-phosphatase J	4.3E-01	138	160	415	286	160	128	62	474	141	309	266	176	108	173	224	347
Microdissected TDLU	Pardo et al, 2014	MUC4	mucin 4, cell surface associated	3.8E-01	17	58	17	5	36	26	12	371	28	83	75	139	93	123	15	16
Microdissected TDLU	Pardo et al, 2014	RMI2	RecQ mediated genome instability 2	1.2E-01	79	48	47	40	34	42	45	47	13	46	55	78	73	91	13	43
Microdissected TDLU	Pardo et al, 2014	SLC26A3	solute carrier family 26 member 3	6.9E-02	1	6	587	49	13	15	106	20	21	142	498	277	388	342	21	12
Microdissected TDLU	Pardo et al, 2014	VSNL1	visinin like 1	1.6E-01	270	255	141	268	174	134	302	415	282	370	313	292	162	409	345	177
					SK10545(	SK10268?	SK105891	SK10726?	SK10596?	SK10437?	SK10616?	SK10556?	*SK105179*	*SK106154*	*SK10671?*	*SK100899*	*SK10580?*	*SK106836*		
Microdissected Stroma	Change between Menstrual Phases	NUTM2B	NUT family member 2B	4.0E-04	2	0	2	1	6	2	1	0	11	11	6	5	9	4		
Microdissected Stroma	Change between Menstrual Phases	TMEM150B	transmembrane protein 150B	2.5E-03	29	6	16	35	31	13	23	21	47	61	35	47	24	39		
Microdissected Stroma	Change between Menstrual Phases	MIR1284	microRNA 1284	3.7E-03	15	11	15	9	15	10	12	4	7	11	5	1	3	2		
Microdissected Stroma	Change between Menstrual Phases	CETN4P	centrin 4, pseudogene	3.8E-03	1	1	5	4	1	5	7	3	5	14	18	7	9	6		
Microdissected Stroma	Change between Menstrual Phases	SIGLEC16	sialic acid binding Ig like lectin 16 (gene/pseudogene)	4.5E-03	6	4	17	11	19	4	12	14	42	22	11	35	17	25		
Microdissected Stroma	Change between Menstrual Phases	RSPO1	R-spondin 1	4.8E-03	128	332	115	176	262	254	155	236	67	77	104	119	140	131		
Microdissected Stroma	Change between Menstrual Phases	SNORD76	small nucleolar RNA, C/D box 76	6.2E-03	371	574	515	297	655	511	622	577	423	435	194	383	166	342		
Microdissected Stroma	Change between Menstrual Phases	ABCC6	ATP binding cassette subfamily C member 6	6.4E-03	25	29	16	58	15	29	50	63	45	74	41	87	99	63		
Microdissected Stroma	Change between Menstrual Phases	MIR548AR	microRNA 548ar	6.8E-03	18	16	11	14	24	12	9	21	14	11	7	7	5	8		
Microdissected Stroma	Change between Menstrual Phases	SNORD41	small nucleolar RNA, C/D box 41	7.0E-03	56	69	61	40	79	50	78	83	47	57	30	50	24	46		
Microdissected Stroma	Change between Menstrual Phases	ROGDI	rogdi homolog	7.2E-03	129	115	124	131	117	133	124	93	216	135	217	214	82	237		
Microdissected Stroma	Change between Menstrual Phases	CYP4F35P	cytochrome P450 family 4 subfamily F member 35, pseudogene	8.8E-03	30	24	5	15	36	23	18	15	49	24	58	34	24	38		
Microdissected Stroma	Change between Menstrual Phases	SNORD58A	small nucleolar RNA, C/D box 58A	9.1E-03	347	324	375	225	396	388	405	440	271	339	173	317	175	295		
Microdissected Stroma	Change between Menstrual Phases	LOC284865	uncharacterized LOC284865	9.1E-03	5	5	6	5	10	8	2	6	5	14	11	14	8	9		
Microdissected Stroma	Change between Menstrual Phases	GSTK1	glutathione S-transferase kappa 1	9.9E-03	897	728	811	863	989	933	977	859	1003	1006	1120	1445	815	1331		
Microdissected Stroma	Change between Menstrual Phases	CHRDL2	chordin like 2	1.0E-02	7	1	9	7	5	2	1	3	10	12	16	2	33	14		
Microdissected Stroma	Change between Menstrual Phases	SLC6A20	solute carrier family 6 member 20	1.4E-02	10	8	13	9	15	14	12	8	8	9	27	28	49	26		
Microdissected Stroma	Change between Menstrual Phases	CSPG4	chondroitin sulfate proteoglycan 4	1.4E-02	565	257	287	317	255	274	394	242	412	427	711	533	305	546		
Microdissected Stroma	Change between Menstrual Phases	GRIN2D	glutamate ionotropic receptor NMDA type subunit 2D	1.5E-02	55	6	18	18	16	9	13	12	37	34	47	23	32	40		
Microdissected Stroma	Change between Menstrual Phases	SNORD87	small nucleolar RNA, C/D box 87	1.5E-02	282	412	495	291	543	467	575	469	271	387	251	343	208	412		
Microdissected Stroma	Change between Menstrual Phases	PKDCC	protein kinase domain containing, cytoplasmic	1.6E-02	399	150	103	129	182	140	250	168	398	425	468	273	127	303		
Microdissected Stroma	Change between Menstrual Phases	HELB	DNA helicase B	1.6E-02	326	426	414	290	421	457	234	264	301	277	172	309	207	274		
Microdissected Stroma	Change between Menstrual Phases	SNORA58	small nucleolar RNA, H/ACA box 58	1.6E-02	36	103	111	35	135	57	83	74	45	63	23	61	27	20		
Microdissected Stroma	Change between Menstrual Phases	MIR22HG	MIR22 host gene	1.6E-02	301	208	203	165	164	169	184	83	294	275	346	250	162	255		
Microdissected Stroma	Change between Menstrual Phases	ACTN2	actinin alpha 2	1.9E-02	11	47	45	8	12	21	7	13	12	32	72	36	86	48		
Microdissected Stroma	Change between Menstrual Phases	TMEM134	transmembrane protein 134	2.0E-02	128	121	99	77	131	110	110	97	131	162	138	196	88	141		
Microdissected Stroma	Change between Menstrual Phases	GLDN	gliomedin	2.0E-02	91	86	101	112	57	26	65	59	34	122	161	195	98	291		
Microdissected Stroma	Change between Menstrual Phases	TRIM16	tripartite motif containing 16	2.1E-02	147	110	138	96	153	127	115	99	146	164	172	161	102	168		
Microdissected Stroma	Change between Menstrual Phases	TMEM106A	transmembrane protein 106A	2.1E-02	594	316	360	304	684	413	324	394	833	837	596	631	355	491		
Microdissected Stroma	Change between Menstrual Phases	TKT	transketolase	2.1E-02	853	702	689	1184	686	786	1178	849	1122	1139	1169	1334	676	1655		
Microdissected Stroma	Change between Menstrual Phases	TMEM53	transmembrane protein 53	2.2E-02	32	34	32	30	29	46	27	42	31	44	59	48	34	49		
Microdissected Stroma	Change between Menstrual Phases	PDHA1	pyruvate dehydrogenase (lipoamide) alpha 1	2.2E-02	596	561	674	784	721	846	812	604	720	865	1072	1064	569	1094		
Microdissected Stroma	Change between Menstrual Phases	FCGBP	Fc fragment of IgG binding protein	2.2E-02	59	70	178	115	253	218	162	145	386	102	449	495	146	1182		
Microdissected Stroma	Change between Menstrual Phases	RPS16P5	ribosomal protein S16 pseudogene 5	2.4E-02	140	145	82	136	183	125	137	73	189	266	147	355	114	153		
Microdissected Stroma	Change between Menstrual Phases	AQP4	aquaporin 4	2.4E-02	0	4	12	2	8	17	16	3	19	4	14	88	91	16		
Microdissected Stroma	Change between Menstrual Phases	ASF1B	anti-silencing function 1B histone chaperone	2.6E-02	11	3	7	15	6	10	9	2	18	6	22	13	8	16		
Microdissected Stroma	Change between Menstrual Phases	ADAMTS9-AS1	ADAMTS9 antisense RNA 1	2.7E-02	61	20	41	34	73	52	53	35	60	83	101	52	40	74		
Microdissected Stroma	Change between Menstrual Phases	C5orf30	chromosome 5 open reading frame 30	2.8E-02	63	52	46	52	53	45	99	40	39	95	91	121	66	82		
Microdissected Stroma	Change between Menstrual Phases	LOC644656	uncharacterized LOC644656	2.9E-02	51	44	51	31	65	58	44	44	36	54	25	27	35	43		
Microdissected Stroma	Change between Menstrual Phases	SIGLEC14	sialic acid binding Ig like lectin 14	3.0E-02	18	7	0	51	8	2	8	12	103	8	69	31	13	34		
Microdissected Stroma	Change between Menstrual Phases	PLB1	phospholipase B1	3.1E-02	66	49	46	92	70	58	67	81	178	71	74	70	78	138		
Microdissected Stroma	Change between Menstrual Phases	BCORL1	BCL6 corepressor-like 1	3.1E-02	973	617	690	544	914	650	636	586	863	1075	1003	960	594	802		
Microdissected Stroma	Change between Menstrual Phases	NDUFS3	NADH:ubiquinone oxidoreductase core subunit S3	3.2E-02	136	101	147	95	95	100	128	132	137	156	179	137	89	190		
Microdissected Stroma	Change between Menstrual Phases	MAFG-AS1	MAFG antisense RNA 1 (head to head)	3.2E-02	16	8	12	1	20	12	19	13	20	17	21	24	13	16		
Microdissected Stroma	Change between Menstrual Phases	NAGPA-AS1	NAGPA antisense RNA 1	3.2E-02	27	19	13	15	29	24	20	43	8	14	18	19	13	19		
Microdissected Stroma	Change between Menstrual Phases	KCNE1	potassium voltage-gated channel subfamily E regulatory subunit	3.3E-02	38	34	52	29	18	51	47	38	37	67	38	104	52	53		
Microdissected Stroma	Change between Menstrual Phases	PNOC	prepronociceptin	3.3E-02	6	7	17	18	14	13	4	23	22	8	34	14	27	23		
Microdissected Stroma	Change between Menstrual Phases	TTC4	tetratricopeptide repeat domain 4	3.3E-02	68	34	73	63	62	61	50	61	72	92	89	66	46	84		
Microdissected Stroma	Change between Menstrual Phases	LOC103091866	uncharacterized LOC103091866	3.4E-02	19	12	14	22	13	23	18	14	19	26	19	65	20	30		
Microdissected Stroma	Change between Menstrual Phases	LOC646214	p21 protein (Cdc42/Rac)-activated kinase 2 pseudogene	3.4E-02	93	126	169	128	163	140	144	186	129	141	198	167	256	222		
Microdissected Stroma	Change between Menstrual Phases	NPR3	natriuretic peptide receptor 3	3.4E-02	107	141	168	188	223	164	333	72	83	158	353	573	358	344		
Microdissected Stroma	Change between Menstrual Phases	KCNK6	potassium two pore domain channel subfamily K member 6	3.4E-02	314	240	223	179	225	198	326	208	406	314	447	360	164	252		
Microdissected Stroma	Change between Menstrual Phases	SNORD111B	small nucleolar RNA, C/D box 111B	3.5E-02	6	28	25	21	29	24	26	21	8	18	10	23	14	19		
Microdissected Stroma	Change between Menstrual Phases	CDK1	cyclin dependent kinase 1	3.7E-02	29	8	23	3	25	20	19	11	53	13	33	24	24	27		
Microdissected Stroma	Change between Menstrual Phases	DHDDS	dehydrodolichyl diphosphate synthase subunit	3.7E-02	461	304	274	562	398	417	494	436	433	575	572	606	355	595		
Microdissected Stroma	Change between Menstrual Phases	SMIM3	small integral membrane protein 3	3.7E-02	79	107	86	107	92	131	120	100	117	167	129	133	85	123		
Microdissected Stroma	Change between Menstrual Phases	GPER1	G protein-coupled estrogen receptor 1	3.8E-02	60	60	73	43	49	125	50	47	63	71	97	126	77	108		
Microdissected Stroma	Change between Menstrual Phases	ARL8A	ADP ribosylation factor like GTPase 8A	3.8E-02	374	208	256	227	259	291	197	198	297	325	364	314	207	381		
Microdissected Stroma	Change between Menstrual Phases	GATSL3	GATS protein like 3	3.8E-02	48	46	52	41	25	77	35	58	56	69	48	108	58	62		
Microdissected Stroma	Change between Menstrual Phases	ACTG1P17	actin gamma 1 pseudogene 17	3.8E-02	17	24	15	15	33	24	35	29	12	25	9	24	18	7		
Microdissected Stroma	Change between Menstrual Phases	ZMYND10	zinc finger MYND-type containing 10	3.9E-02	10	5	11	4	8	21	18	7	13	18	9	29	30	11		
Microdissected Stroma	Change between Menstrual Phases	SSTR3	somatostatin receptor 3	3.9E-02	10	0	4	10	6	23	3	3	5	14	11	13	21	26		
Microdissected Stroma	Change between Menstrual Phases	ZNF660	zinc finger protein 660	3.9E-02	190	171	213	142	186	176	137	148	126	203	128	135	127	127		
Microdissected Stroma	Change between Menstrual Phases	KLK14	kallikrein related peptidase 14	4.0E-02	14	8	8	5	3	0	2	4	8	5	13	47	80	5		
Microdissected Stroma	Change between Menstrual Phases	MRPS7	mitochondrial ribosomal protein S7	4.0E-02	225	112	199	158	158	164	166	119	181	194	275	230	115	317		
Microdissected Stroma	Change between Menstrual Phases	FAM87B	family with sequence similarity 87 member B	4.1E-02	5	3	1	5	3	5	5	8	7	9	8	5	2	13		
Microdissected Stroma	Change between Menstrual Phases	POLR2E	RNA polymerase II subunit E	4.3E-02	365	278	279	430	342	412	459	415	413	513	548	475	252	664		
Microdissected Stroma	Change between Menstrual Phases	TRIM8	tripartite motif containing 8	4.3E-02	1213	1069	1146	1195	1259	1173	1169	1020	1139	1569	1303	1562	918	1570		
Microdissected Stroma	Change between Menstrual Phases	MYO1C	myosin IC	4.4E-02	2643	1979	2225	2682	2299	2860	3270	1937	2310	3153	4199	3836	1929	3525		
Microdissected Stroma	Change between Menstrual Phases	BCAP31	B-cell receptor-associated protein 31	4.4E-02	596	525	532	614	514	640	677	570	540	764	838	822	430	848		
Microdissected Stroma	Change between Menstrual Phases	KCNK2	potassium two pore domain channel subfamily K member 2	4.6E-02	45	181	129	91	43	96	64	34	12	64	35	44	35	78		
Microdissected Stroma	Change between Menstrual Phases	SPACA6P-AS	SPACA6P antisense RNA	4.6E-02	18	16	8	13	37	21	17	24	25	42	17	20	40	27		
Microdissected Stroma	Change between Menstrual Phases	SNORD61	small nucleolar RNA, C/D box 61	4.8E-02	68	157	165	96	167	87	128	107	96	126	53	75	86	101		
Microdissected Stroma	Change between Menstrual Phases	SLC1A4	solute carrier family 1 member 4	4.8E-02	269	291	282	261	273	270	245	184	281	290	354	740	278	311		
Microdissected Stroma	Change between Menstrual Phases	SLC4A4	solute carrier family 4 member 4	4.9E-02	85	71	59	122	34	68	181	92	25	142	159	190	141	175		
					FK107267	FK10433?	FK102687	FK105891	FK106169	FK10556?	FK10545(	*FK105179*	*FK106154*	*FK10671?*	*FK105868*					
Microdissected Adipose Tissue	Change between Menstrual Phases	ABCA12	ATP binding cassette subfamily A member 12	5.2E-02	9	4	20	1	18	4	5	1	3	0	3					
Microdissected Adipose Tissue	Change between Menstrual Phases	ACSL3	acyl-CoA synthetase long-chain family member 3	4.2E-02	788	468	436	472	795	890	749	1001	1281	1148	461					
Microdissected Adipose Tissue	Change between Menstrual Phases	ADAMTS16	ADAM metallopeptidase with thrombospondin type 1 motif 16	2.0E-02	12	23	39	61	200	37	463	1671	295	1731	4					
Microdissected Adipose Tissue	Change between Menstrual Phases	ADCY8	adenylate cyclase 8	2.5E-02	0	0	3	17	21	2	10	60	43	1	20					
Microdissected Adipose Tissue	Change between Menstrual Phases	AGAP7P	ArfGAP with GTPase domain, ankyrin repeat and PH domain 7, p	2.7E-02	4	4	10	8	40	30	8	19	73	15	81					
Microdissected Adipose Tissue	Change between Menstrual Phases	ANKRD13B	ankyrin repeat domain 13B	1.4E-02	56	81	136	148	198	121	223	627	434	171	198					
Microdissected Adipose Tissue	Change between Menstrual Phases	ANXA8L1	annexin A8-like 1	4.1E-02	17	22	11	17	29	0	7	0	12	5	0					
Microdissected Adipose Tissue	Change between Menstrual Phases	APOBEC3D	apolipoprotein B mRNA editing enzyme catalytic subunit 3D	1.8E-02	22	31	47	65	93	58	57	140	120	87	50					
Microdissected Adipose Tissue	Change between Menstrual Phases	ASAP3	ArfGAP with SH3 domain, ankyrin repeat and PH domain 3	4.7E-02	453	379	664	756	780	580	397	1232	1137	599	507					
Microdissected Adipose Tissue	Change between Menstrual Phases	ATP8B2	ATPase phospholipid transporting 8B2	8.5E-03	424	379	585	650	585	394	448	1344	995	683	568					
Microdissected Adipose Tissue	Change between Menstrual Phases	BCORL1	BCL6 corepressor-like 1	2.4E-03	265	214	392	451	530	395	260	971	831	569	514					
Microdissected Adipose Tissue	Change between Menstrual Phases	C14orf37	chromosome 14 open reading frame 37	5.4E-03	132	137	137	156	194	238	183	538	303	282	231					
Microdissected Adipose Tissue	Change between Menstrual Phases	CA11	carbonic anhydrase 11	2.5E-03	17	15	55	44	91	56	29	149	92	82	108					
Microdissected Adipose Tissue	Change between Menstrual Phases	CD180	CD180 molecule	3.7E-02	30	28	11	7	14	34	31	119	22	78	18					
Microdissected Adipose Tissue	Change between Menstrual Phases	CNN2	calponin 2	3.6E-02	611	678	354	643	898	953	503	1011	965	1326	593					
Microdissected Adipose Tissue	Change between Menstrual Phases	CORIN	corin, serine peptidase	2.1E-02	14	16	34	5	20	23	27	888	66	552	10					
Microdissected Adipose Tissue	Change between Menstrual Phases	CTAGE6	CTAGE family member 6	4.5E-02	4	4	4	3	11	8	2	10	13	8	5					
Microdissected Adipose Tissue	Change between Menstrual Phases	DEFB124	defensin beta 124	2.6E-02	0	3	18	0	0	2	8	31	11	13	8					
Microdissected Adipose Tissue	Change between Menstrual Phases	DLG4	discs large MAGUK scaffold protein 4	1.3E-02	96	128	201	155	245	154	115	407	281	203	201					
Microdissected Adipose Tissue	Change between Menstrual Phases	ELF4	E74 like ETS transcription factor 4	4.3E-02	259	156	195	186	209	181	164	499	342	211	161					
Microdissected Adipose Tissue	Change between Menstrual Phases	EMX2OS	EMX2 opposite strand/antisense RNA	2.4E-02	281	249	103	179	310	381	216	560	601	330	226					
Microdissected Adipose Tissue	Change between Menstrual Phases	FCER2	Fc fragment of IgE receptor II	2.5E-02	7	26	30	49	32	68	24	78	142	78	22					
Microdissected Adipose Tissue	Change between Menstrual Phases	G6PD	glucose-6-phosphate dehydrogenase	3.7E-02	330	239	192	239	293	346	357	357	394	551	270					
Microdissected Adipose Tissue	Change between Menstrual Phases	HCN1	hyperpolarization activated cyclic nucleotide gated potassium ch	4.0E-04	1	1	0	1	2	1	1	4	4	2	3					
Microdissected Adipose Tissue	Change between Menstrual Phases	HIST1H2BH	histone cluster 1 H2B family member h	4.8E-02	15	11	28	7	26	15	7	66	11	56	13					
Microdissected Adipose Tissue	Change between Menstrual Phases	HIST1H3I	histone cluster 1 H3 family member i	7.7E-03	6	17	21	6	25	45	24	74	24	58	48					
Microdissected Adipose Tissue	Change between Menstrual Phases	HIST1H4L	histone cluster 1 H4 family member l	3.4E-02	4	1	2	4	16	19	13	19	16	34	9					
Microdissected Adipose Tissue	Change between Menstrual Phases	HLA-H	major histocompatibility complex, class I, H (pseudogene)	2.9E-02	119	111	183	286	186	110	145	438	310	332	99					
Microdissected Adipose Tissue	Change between Menstrual Phases	HRH1	histamine receptor H1	4.8E-02	135	144	100	132	132	195	166	232	287	280	70					
Microdissected Adipose Tissue	Change between Menstrual Phases	KCND1	potassium voltage-gated channel subfamily D member 1	5.6E-03	42	30	19	31	34	68	28	78	73	49	62					
Microdissected Adipose Tissue	Change between Menstrual Phases	KIAA0226L	NA	2.4E-02	36	65	17	67	59	60	58	111	121	155	24					
Microdissected Adipose Tissue	Change between Menstrual Phases	KRI1	KRI1 homolog	3.9E-02	400	335	579	423	745	361	214	705	633	642	515					
Microdissected Adipose Tissue	Change between Menstrual Phases	KRT1	keratin 1	2.8E-02	19	28	6	19	1	2	13	1	2	0	2					
Microdissected Adipose Tissue	Change between Menstrual Phases	KYNU	kynureninase	5.1E-02	15	36	12	64	23	31	39	105	65	62	13					
Microdissected Adipose Tissue	Change between Menstrual Phases	LILRB1	leukocyte immunoglobulin like receptor B1	3.8E-02	44	47	32	31	87	45	39	142	64	83	43					
Microdissected Adipose Tissue	Change between Menstrual Phases	LINC01143	long intergenic non-protein coding RNA 1143	4.1E-02	0	0	3	1	1	1	1	0	3	3	6					
Microdissected Adipose Tissue	Change between Menstrual Phases	LOC101928034	uncharacterized LOC101928034	8.3E-03	70	58	21	65	47	103	143	296	180	245	61					
Microdissected Adipose Tissue	Change between Menstrual Phases	LOC101929122	uncharacterized LOC101929122	2.4E-02	0	0	1	3	4	2	2	21	4	1	11					
Microdissected Adipose Tissue	Change between Menstrual Phases	LOC101929331	uncharacterized LOC101929331	2.9E-02	18	20	29	17	8	16	4	42	33	58	7					
Microdissected Adipose Tissue	Change between Menstrual Phases	LPAR5	lysophosphatidic acid receptor 5	2.9E-02	11	35	4	8	19	9	8	28	28	49	14					
Microdissected Adipose Tissue	Change between Menstrual Phases	MEG8	maternally expressed 8 (non-protein coding)	2.4E-02	32	31	167	82	189	70	101	812	319	207	113					
Microdissected Adipose Tissue	Change between Menstrual Phases	MICALL1	MICAL like 1	8.7E-03	374	222	256	296	520	283	321	838	643	627	299					
Microdissected Adipose Tissue	Change between Menstrual Phases	MIR27B	microRNA 27b	2.0E-02	17	16	35	13	28	21	17	9	13	6	15					
Microdissected Adipose Tissue	Change between Menstrual Phases	MIR487A	microRNA 487a	9.5E-04	0	3	0	2	3	3	8	18	12	7	11					
Microdissected Adipose Tissue	Change between Menstrual Phases	MIR495	microRNA 495	7.7E-03	0	0	3	4	9	4	2	25	18	16	1					
Microdissected Adipose Tissue	Change between Menstrual Phases	MIR645	microRNA 645	3.6E-02	6	14	8	10	10	5	9	14	14	15	8					
Microdissected Adipose Tissue	Change between Menstrual Phases	NFATC4	nuclear factor of activated T-cells 4	2.6E-02	228	253	729	838	613	301	407	1356	819	476	893					
Microdissected Adipose Tissue	Change between Menstrual Phases	NGF	nerve growth factor	2.9E-02	21	1	7	5	19	3	13	61	39	19	5					
Microdissected Adipose Tissue	Change between Menstrual Phases	NHSL2	NHS like 2	3.3E-02	262	252	322	334	234	317	257	291	453	400	289					
Microdissected Adipose Tissue	Change between Menstrual Phases	NPHP4	nephrocystin 4	1.9E-02	136	79	133	146	179	81	82	346	191	292	92					
Microdissected Adipose Tissue	Change between Menstrual Phases	NPTXR	neuronal pentraxin receptor	1.1E-02	88	79	87	133	82	70	67	181	130	140	89					
Microdissected Adipose Tissue	Change between Menstrual Phases	OR51I1	olfactory receptor family 51 subfamily I member 1	3.8E-02	7	0	0	0	1	3	1	4	6	5	3					
Microdissected Adipose Tissue	Change between Menstrual Phases	PARVG	parvin gamma	4.0E-02	114	67	93	57	93	87	66	250	122	185	41					
Microdissected Adipose Tissue	Change between Menstrual Phases	PLA2G2A	phospholipase A2 group IIA	2.8E-02	56	7	29	3	21	71	15	193	58	194	0					
Microdissected Adipose Tissue	Change between Menstrual Phases	PLXNC1	plexin C1	4.8E-02	338	278	166	399	367	331	340	888	1103	285	211					
Microdissected Adipose Tissue	Change between Menstrual Phases	PRCAT47	prostate cancer-associated 47	7.4E-03	0	0	2	2	2	4	3	13	2	7	7					
Microdissected Adipose Tissue	Change between Menstrual Phases	PRKAR1B	protein kinase cAMP-dependent type I regulatory subunit beta	3.8E-02	139	98	160	125	179	126	91	170	210	243	111					
Microdissected Adipose Tissue	Change between Menstrual Phases	PTPRD-AS1	PTPRD antisense RNA 1	3.3E-02	4	7	2	4	11	10	1	11	17	33	3					
Microdissected Adipose Tissue	Change between Menstrual Phases	RARG	retinoic acid receptor gamma	1.6E-02	229	291	264	392	411	351	355	672	639	407	318					
Microdissected Adipose Tissue	Change between Menstrual Phases	RYR1	ryanodine receptor 1	5.0E-03	150	303	265	320	337	346	281	677	587	606	258					
Microdissected Adipose Tissue	Change between Menstrual Phases	SARM1	sterile alpha and TIR motif containing 1	1.8E-02	169	110	165	229	313	164	176	345	358	271	200					
Microdissected Adipose Tissue	Change between Menstrual Phases	SNAP25	synaptosome associated protein 25	2.5E-02	12	10	8	4	15	16	14	255	41	91	13					
Microdissected Adipose Tissue	Change between Menstrual Phases	SNORD114-14	small nucleolar RNA, C/D box 114-14	3.1E-02	9	14	17	9	66	33	15	65	83	52	17					
Microdissected Adipose Tissue	Change between Menstrual Phases	TLDC2	TBC/LysM-associated domain containing 2	4.7E-02	3	1	7	7	5	4	6	7	9	10	4					
Microdissected Adipose Tissue	Change between Menstrual Phases	TMEM45A	transmembrane protein 45A	3.1E-02	18	30	15	23	28	23	26	47	39	80	14					
Microdissected Adipose Tissue	Change between Menstrual Phases	TRIM59	tripartite motif containing 59	1.7E-02	9	13	15	16	12	8	4	24	17	11	22					
Microdissected Adipose Tissue	Change between Menstrual Phases	TTC39C-AS1	TTC39C antisense RNA 1	4.1E-02	0	3	1	1	7	2	3	3	7	13	3					
Microdissected Adipose Tissue	Change between Menstrual Phases	ZNF469	zinc finger protein 469	1.9E-02	183	199	335	251	507	171	219	1904	737	429	482					
Microdissected Adipose Tissue	Change between Menstrual Phases	ZNF667	zinc finger protein 667	4.9E-02	73	69	127	125	213	144	100	219	203	210	96					
Microdissected Adipose Tissue	Change between Menstrual Phases	ZWINT	ZW10 interacting kinetochore protein	4.0E-02	13	18	15	17	33	13	8	66	21	82	6					

*unpaired two-tails t-test p value

Supplementary Table 4: Breast Epithelium-specific differentially expressed genes between susceptible and healthy control breast.

Symbol	geneName	FC*	log2FC	PValue	FDR
AQP7	aquaporin 7	2.49	1.32	1.16E-05	0.001
AKR1C1	aldo-keto reductase family 1 member C1	4.34	2.12	1.18E-05	0.001
XIRP2	xin actin binding repeat containing 2	7.36	2.88	2.44E-05	0.002
SIM1	single-minded family bHLH transcription factor 1	5.44	2.44	6.23E-05	0.003
RBP4	retinol binding protein 4	3.66	1.87	2.77E-04	0.010
PLIN1	perilipin 1	3.92	1.97	3.10E-04	0.010
SORCS1	sortilin related VPS10 domain containing receptor 1	3.01	1.59	3.72E-04	0.010
ZFP57	ZFP57 zinc finger protein	-28.97	-4.86	3.76E-04	0.010
LIPE	lipase E, hormone sensitive type	2.19	1.13	4.42E-04	0.010
MAPK10	mitogen-activated protein kinase 10	2.12	1.09	4.43E-04	0.010
GATA2-AS1	GATA2 antisense RNA 1	2.27	1.18	5.40E-04	0.010
SLED1	proteoglycan 3 pseudogene	2.98	1.58	6.72E-04	0.010
G0S2	G0/G1 switch 2	2.93	1.55	6.73E-04	0.010
CIDEA	cell death-inducing DFFA-like effector a	3.60	1.85	6.85E-04	0.010
NAV3	neuron navigator 3	1.38	0.47	7.01E-04	0.010
STAB2	stabilin 2	2.43	1.28	7.34E-04	0.010
ACVR1C	activin A receptor type 1C	2.19	1.13	1.01E-03	0.013
MYH2	myosin heavy chain 2	6.88	2.78	1.06E-03	0.013
RPS16P5	ribosomal protein S16 pseudogene 5	2.54	1.34	1.32E-03	0.015
FAM221B	family with sequence similarity 221 member B	2.75	1.46	1.56E-03	0.017
CRTC3-AS1	CRTC3 antisense RNA 1	-1.87	-0.90	1.64E-03	0.017
GABRE	gamma-aminobutyric acid type A receptor epsilon subunit	2.21	1.14	1.80E-03	0.018
TNNC2	troponin C2, fast skeletal type	2.37	1.24	1.82E-03	0.018
ADIPOQ	adiponectin, C1Q and collagen domain containing	3.82	1.93	1.93E-03	0.018
INO80B	INO80 complex subunit B	1.87	0.91	2.01E-03	0.018
AQP7P1	aquaporin 7 pseudogene 1	2.06	1.04	2.30E-03	0.020
ACTA1	actin, alpha 1, skeletal muscle	5.40	2.43	2.41E-03	0.020
LOC10192696	uncharacterized LOC101926960	4.33	2.12	2.63E-03	0.020
PLIN4	perilipin 4	2.53	1.34	2.66E-03	0.020
SLC19A3	solute carrier family 19 member 3	3.33	1.74	2.67E-03	0.020
GPD1	glycerol-3-phosphate dehydrogenase 1	2.92	1.55	3.19E-03	0.023
GRIA1	glutamate ionotropic receptor AMPA type subunit 1	2.89	1.53	3.43E-03	0.024
ABCC6	ATP binding cassette subfamily C member 6	2.13	1.09	3.52E-03	0.024
TRHDE-AS1	TRHDE antisense RNA 1	3.86	1.95	4.01E-03	0.025
MYOZ2	myozenin 2	2.13	1.09	4.05E-03	0.025
TUSC5	tumor suppressor candidate 5	4.07	2.03	4.09E-03	0.025
CD36	CD36 molecule	1.72	0.79	4.25E-03	0.026
NOVA1	NOVA alternative splicing regulator 1	1.58	0.66	4.81E-03	0.028
FABP4	fatty acid binding protein 4	2.22	1.15	5.55E-03	0.031
SDS	serine dehydratase	2.42	1.27	5.82E-03	0.031
SNORA13	small nucleolar RNA, H/ACA box 13	-4.30	-2.10	6.11E-03	0.031
FAM86C2P	family with sequence similarity 86, member A pseudogene	-1.42	-0.50	6.13E-03	0.031
TRAF5	TNF receptor associated factor 5	1.40	0.49	6.48E-03	0.031
KLRF1	killer cell lectin like receptor F1	2.38	1.25	6.50E-03	0.031
LOC10192796	uncharacterized LOC101927969	2.12	1.08	6.81E-03	0.031
SLC5A4	solute carrier family 5 member 4	2.01	1.01	6.85E-03	0.031
SAA4	serum amyloid A4, constitutive	-2.46	-1.30	6.86E-03	0.031
HEPACAM	hepatic and glial cell adhesion molecule	2.38	1.25	7.18E-03	0.031
SLC30A1	solute carrier family 30 member 1	1.29	0.36	7.25E-03	0.031
KLB	klotho beta	2.19	1.13	7.44E-03	0.031
FAM214A	family with sequence similarity 214 member A	1.64	0.72	7.48E-03	0.031
SLC4A10	solute carrier family 4 member 10	2.77	1.47	7.81E-03	0.031
SLC2A1-AS1	SLC2A1 antisense RNA 1	2.11	1.07	8.07E-03	0.031
MMP27	matrix metallopeptidase 27	-2.85	-1.51	8.08E-03	0.031
F8	coagulation factor VIII	1.30	0.38	8.22E-03	0.031

PAQR5	progestin and adipoQ receptor family member 5	1.92	0.94	8.28E-03	0.031
FN3KRP	fructosamine 3 kinase related protein	-1.36	-0.44	8.29E-03	0.031
PCAT19	prostate cancer associated transcript 19 (non-protein coding)	1.51	0.60	8.36E-03	0.031
CBR3-AS1	CBR3 antisense RNA 1	1.71	0.77	8.36E-03	0.031
TMEM229B	transmembrane protein 229B	-1.71	-0.77	8.43E-03	0.031
TSPAN11	tetraspanin 11	-1.72	-0.78	8.58E-03	0.031
DLEU1	deleted in lymphocytic leukemia 1	1.55	0.63	8.74E-03	0.031
PCCA	propionyl-CoA carboxylase alpha subunit	1.34	0.42	9.04E-03	0.031
TPRXL	tetrapeptide repeat homeobox like	1.85	0.89	9.07E-03	0.031
NR5A2	nuclear receptor subfamily 5 group A member 2	1.53	0.61	9.67E-03	0.032
APOB	apolipoprotein B	3.02	1.60	9.73E-03	0.032
TEKT4P2	tektin 4 pseudogene 2	-1.60	-0.68	9.77E-03	0.032
MIR3619	microRNA 3619	1.94	0.95	1.01E-02	0.032
RAET1E-AS1	RAET1E antisense RNA 1	-1.91	-0.93	1.02E-02	0.032
HSD17B13	hydroxysteroid 17-beta dehydrogenase 13	1.83	0.87	1.02E-02	0.032
EMX2	empty spiracles homeobox 2	-2.11	-1.08	1.02E-02	0.032
KCNMA1	potassium calcium-activated channel subfamily M alpha 1	1.52	0.61	1.06E-02	0.032
CPEB2	cytoplasmic polyadenylation element binding protein 2	1.33	0.41	1.06E-02	0.032
CXCR6	C-X-C motif chemokine receptor 6	-1.74	-0.80	1.09E-02	0.033
GYG2	glycogenin 2	1.79	0.84	1.13E-02	0.033
LILRA1	leukocyte immunoglobulin like receptor A1	-1.93	-0.95	1.13E-02	0.033
FAM169A	family with sequence similarity 169 member A	1.36	0.45	1.14E-02	0.033
CPNE5	copine 5	-1.78	-0.83	1.17E-02	0.033
KC6	keratoconus gene 6	5.30	2.41	1.24E-02	0.035
TRIM5	tripartite motif containing 5	-1.24	-0.31	1.26E-02	0.035
LOC10272335	uncharacterized LOC102723354	1.75	0.80	1.27E-02	0.035
LEP	leptin	3.76	1.91	1.33E-02	0.036
GUCY1A3	guanylate cyclase 1 soluble subunit alpha	-1.44	-0.52	1.40E-02	0.036
FAM129C	family with sequence similarity 129 member C	2.44	1.29	1.44E-02	0.036
CPA4	carboxypeptidase A4	-1.81	-0.86	1.44E-02	0.036
PCDHAC1	protocadherin alpha subfamily C, 1	1.75	0.81	1.44E-02	0.036
ARMCX4	armadillo repeat containing, X-linked 4	1.47	0.56	1.45E-02	0.036
C19orf18	chromosome 19 open reading frame 18	1.62	0.70	1.45E-02	0.036
SLC26A7	solute carrier family 26 member 7	1.67	0.74	1.47E-02	0.036
SARDH	sarcosine dehydrogenase	-1.44	-0.52	1.47E-02	0.036
C19orf73	chromosome 19 open reading frame 73	1.57	0.65	1.54E-02	0.037
RGPD4	RANBP2-like and GRIP domain containing 4	-2.12	-1.09	1.54E-02	0.037
COLCA1	colorectal cancer associated 1	1.58	0.66	1.60E-02	0.038
LPL	lipoprotein lipase	1.79	0.84	1.61E-02	0.038
MIR548N	microRNA 548n	2.06	1.05	1.63E-02	0.038
NIPAL3	NIPA like domain containing 3	-1.28	-0.36	1.67E-02	0.038
RAB38	RAB38, member RAS oncogene family	-1.35	-0.43	1.69E-02	0.038
ST6GALNAC5	ST6 N-acetylgalactosaminide alpha-2,6-sialyltransferase 5	-1.79	-0.84	1.71E-02	0.038
ZNF876P	zinc finger protein 876, pseudogene	1.33	0.41	1.72E-02	0.038
ALDH1L1	aldehyde dehydrogenase 1 family member L1	1.69	0.76	1.74E-02	0.038
CLDN19	claudin 19	1.46	0.55	1.75E-02	0.038
RPGRIP1	retinitis pigmentosa GTPase regulator interacting protein 1	1.67	0.74	1.77E-02	0.038
HCG27	HLA complex group 27 (non-protein coding)	1.72	0.78	1.78E-02	0.038
LINC01132	long intergenic non-protein coding RNA 1132	-1.72	-0.78	1.79E-02	0.038
PTH2R	parathyroid hormone 2 receptor	1.61	0.69	1.88E-02	0.039
C15orf57	chromosome 15 open reading frame 57	-1.23	-0.29	1.89E-02	0.039
KCND2	potassium voltage-gated channel subfamily D member 2	1.86	0.89	1.89E-02	0.039
RNF24	ring finger protein 24	-1.26	-0.33	1.92E-02	0.040
COL8A2	collagen type VIII alpha 2 chain	-1.36	-0.44	1.96E-02	0.040
CEP19	centrosomal protein 19	-1.46	-0.54	1.99E-02	0.040
C1orf21	chromosome 1 open reading frame 21	1.39	0.48	2.02E-02	0.040
ARMCX2	armadillo repeat containing, X-linked 2	-1.21	-0.28	2.04E-02	0.040
HTR7P1	5-hydroxytryptamine receptor 7 pseudogene 1	1.31	0.39	2.05E-02	0.040
WDYHV1	WDYHV motif containing 1	1.34	0.42	2.07E-02	0.040

GUCY1B3	guanylate cyclase 1 soluble subunit beta	-1.37	-0.45	2.14E-02	0.040
DHRS12	dehydrogenase/reductase 12	1.26	0.33	2.16E-02	0.040
SLC41A2	solute carrier family 41 member 2	1.50	0.59	2.17E-02	0.040
TESPA1	thymocyte expressed, positive selection associated 1	1.53	0.61	2.18E-02	0.040
PDE2A	phosphodiesterase 2A	1.47	0.56	2.20E-02	0.040
LINC01296	long intergenic non-protein coding RNA 1296	1.78	0.84	2.20E-02	0.040
CHST6	carbohydrate sulfotransferase 6	-2.24	-1.16	2.21E-02	0.040
PDE3B	phosphodiesterase 3B	1.47	0.56	2.25E-02	0.040
ANP32A-IT1	ANP32A intronic transcript 1	1.49	0.58	2.29E-02	0.040
ZNF793-AS1	ZNF793 antisense RNA 1 (head to head)	1.75	0.81	2.32E-02	0.040
LOC728730	uncharacterized LOC728730	1.28	0.36	2.33E-02	0.040
NPIPB11	nuclear pore complex interacting protein family member B11	1.67	0.74	2.34E-02	0.040
LOC10099663	killer cell lectin like receptor F1	2.37	1.25	2.38E-02	0.040
ADRA2A	adrenoceptor alpha 2A	1.87	0.91	2.38E-02	0.040
IRX6	iroquois homeobox 6	1.91	0.93	2.39E-02	0.040
HIF1A-AS1	HIF1A antisense RNA 1	-1.87	-0.90	2.40E-02	0.040
YEATS2	YEATS domain containing 2	-1.18	-0.24	2.46E-02	0.040
TIMP4	TIMP metallopeptidase inhibitor 4	1.73	0.79	2.49E-02	0.040
PLEKHD1	pleckstrin homology and coiled-coil domain containing D1	1.99	1.00	2.50E-02	0.040
GATA2	GATA binding protein 2	1.60	0.68	2.51E-02	0.040
LOC440300	chondroitin sulfate proteoglycan 4 pseudogene	1.71	0.77	2.52E-02	0.040
SPRN	shadow of prion protein homolog (zebrafish)	-1.89	-0.92	2.52E-02	0.040
KLHL7-AS1	KLHL7 antisense RNA 1 (head to head)	-1.95	-0.96	2.52E-02	0.040
SLC25A33	solute carrier family 25 member 33	1.28	0.36	2.53E-02	0.040
GPRIN2	G protein regulated inducer of neurite outgrowth 2	-1.40	-0.48	2.55E-02	0.040
ELOVL5	ELOVL fatty acid elongase 5	1.84	0.88	2.55E-02	0.040
PPP1R15B	protein phosphatase 1 regulatory subunit 15B	1.17	0.22	2.55E-02	0.040
HDC	histidine decarboxylase	2.18	1.12	2.61E-02	0.040
PIGL	phosphatidylinositol glycan anchor biosynthesis class L	1.41	0.50	2.62E-02	0.040
KLF9	Kruppel like factor 9	1.23	0.30	2.64E-02	0.040
SKAP1	src kinase associated phosphoprotein 1	-1.37	-0.46	2.67E-02	0.040
CIDEC	cell death inducing DFFA like effector c	3.01	1.59	2.67E-02	0.040
LOC10192806	uncharacterized LOC101928069	-1.48	-0.57	2.68E-02	0.040
LOC642236	NA	1.67	0.74	2.75E-02	0.041
TMEM132C	transmembrane protein 132C	1.75	0.80	2.78E-02	0.041
CYP39A1	cytochrome P450 family 39 subfamily A member 1	-1.39	-0.48	2.79E-02	0.041
PHC1	polyhomeotic homolog 1	1.22	0.29	2.87E-02	0.042
HRASLS5	HRAS like suppressor family member 5	1.34	0.42	2.87E-02	0.042
SORD	sorbitol dehydrogenase	1.84	0.88	2.88E-02	0.042
FZD4	frizzled class receptor 4	1.26	0.34	2.91E-02	0.042
IFITM10	interferon induced transmembrane protein 10	1.56	0.64	2.91E-02	0.042
LRRN3	leucine rich repeat neuronal 3	-1.48	-0.56	2.93E-02	0.042
FREM2	FRAS1 related extracellular matrix protein 2	-1.60	-0.67	2.99E-02	0.042
CD8A	CD8a molecule	-1.41	-0.50	3.04E-02	0.042
SLC25A30-AS	SLC25A30 antisense RNA 1	1.64	0.71	3.07E-02	0.042
THSD1	thrombospondin type 1 domain containing 1	1.24	0.31	3.08E-02	0.042
KCNIP2	potassium voltage-gated channel interacting protein 2	1.75	0.81	3.17E-02	0.042
THEM4	thioesterase superfamily member 4	1.29	0.36	3.18E-02	0.042
CHRNB2	cholinergic receptor nicotinic beta 2 subunit	1.46	0.55	3.18E-02	0.042
FAM81B	family with sequence similarity 81 member B	2.03	1.02	3.19E-02	0.042
SMO	smoothened, frizzled class receptor	-1.32	-0.41	3.20E-02	0.042
RGS22	regulator of G-protein signaling 22	1.59	0.67	3.20E-02	0.042
MAPK8	mitogen-activated protein kinase 8	1.26	0.34	3.20E-02	0.042
PPID	peptidylprolyl isomerase D	-1.20	-0.27	3.20E-02	0.042
ANKRD29	ankyrin repeat domain 29	1.34	0.42	3.21E-02	0.042
POU5F1P5	POU class 5 homeobox 1 pseudogene 5	1.71	0.77	3.21E-02	0.042
RNF130	ring finger protein 130	-1.20	-0.26	3.25E-02	0.042
TPSAB1	tryptase alpha/beta 1	2.08	1.06	3.30E-02	0.042
CXCR5	C-X-C motif chemokine receptor 5	2.29	1.20	3.32E-02	0.042

IL27RA	interleukin 27 receptor subunit alpha	-1.33	-0.41	3.32E-02	0.042
NEAT1	nuclear paraspeckle assembly transcript 1 (non-protein coding)	1.54	0.63	3.33E-02	0.042
MMRN1	multimerin 1	2.33	1.22	3.38E-02	0.043
LTBP1	latent transforming growth factor beta binding protein 1	-1.34	-0.42	3.44E-02	0.043
SDC2	syndecan 2	-1.32	-0.40	3.49E-02	0.043
COLCA2	colorectal cancer associated 2	1.52	0.60	3.60E-02	0.044
THOC6	THO complex 6	-1.28	-0.36	3.62E-02	0.044
PCDHB13	protocadherin beta 13	1.30	0.38	3.62E-02	0.044
TBP	TATA-box binding protein	1.20	0.26	3.66E-02	0.045
ACACB	acetyl-CoA carboxylase beta	1.34	0.42	3.67E-02	0.045
GPX3	glutathione peroxidase 3	1.51	0.59	3.79E-02	0.045
LINC01465	long intergenic non-protein coding RNA 1465	1.49	0.57	3.80E-02	0.045
LOC401052	uncharacterized LOC401052	1.44	0.53	3.80E-02	0.045
MURC	muscle related coiled-coil protein	-1.69	-0.75	3.82E-02	0.045
OXER1	oxoeicosanoid receptor 1	1.54	0.62	3.83E-02	0.045
LOC10012951	uncharacterized LOC100129518	1.58	0.66	3.83E-02	0.045
AMH	anti-Mullerian hormone	1.98	0.98	3.85E-02	0.045
RAPGEF3	Rap guanine nucleotide exchange factor 3	1.41	0.50	3.90E-02	0.045
ZMAT4	zinc finger matrin-type 4	1.77	0.83	3.90E-02	0.045
FAM181B	family with sequence similarity 181 member B	-1.55	-0.63	3.98E-02	0.046
STYK1	serine/threonine/tyrosine kinase 1	1.67	0.74	3.99E-02	0.046
PKIA	protein kinase (cAMP-dependent, catalytic) inhibitor alpha	1.38	0.46	4.01E-02	0.046
FAM90A1	family with sequence similarity 90 member A1	-1.62	-0.70	4.08E-02	0.046
ROR2	receptor tyrosine kinase like orphan receptor 2	-1.30	-0.38	4.15E-02	0.046
PRDM1	PR/SET domain 1	-1.26	-0.33	4.15E-02	0.046
EMP1	epithelial membrane protein 1	1.39	0.48	4.16E-02	0.046
MUC3A	mucin 3A, cell surface associated	1.51	0.59	4.16E-02	0.046
UNC119B	unc-119 lipid binding chaperone B	-1.16	-0.21	4.16E-02	0.046
AIM2	absent in melanoma 2	1.75	0.80	4.17E-02	0.046
MYO16	myosin XVI	2.17	1.12	4.18E-02	0.046
CGNL1	cingulin like 1	-1.21	-0.28	4.19E-02	0.046
MALAT1	metastasis associated lung adenocarcinoma transcript 1 (non-pro	1.43	0.52	4.24E-02	0.046
MIR24-1	microRNA 24-1	1.81	0.86	4.26E-02	0.046
HEY2	hes related family bHLH transcription factor with YRPW motif 2	-1.39	-0.47	4.32E-02	0.046
LOC10013134	RAD52 motif containing 1 pseudogene	1.62	0.70	4.34E-02	0.046
SPTLC3	serine palmitoyltransferase long chain base subunit 3	-1.21	-0.28	4.42E-02	0.047
SSTR2	somatostatin receptor 2	1.78	0.83	4.45E-02	0.047
ANAPC13	anaphase promoting complex subunit 13	1.20	0.26	4.46E-02	0.047
ABCB5	ATP binding cassette subfamily B member 5	1.96	0.97	4.47E-02	0.047
NRIP2	nuclear receptor interacting protein 2	-1.50	-0.58	4.52E-02	0.047
PCOLCE2	procollagen C-endopeptidase enhancer 2	2.30	1.20	4.54E-02	0.047
BTNL9	butyrophilin like 9	1.45	0.54	4.54E-02	0.047
EFNB3	ephrin B3	-1.25	-0.33	4.68E-02	0.048
HOXD3	homeobox D3	1.49	0.57	4.77E-02	0.049
SNX29P2	sorting nexin 29 pseudogene 2	1.44	0.53	4.82E-02	0.049
PPARG	peroxisome proliferator activated receptor gamma	1.37	0.45	4.93E-02	0.050
LINC00612	long intergenic non-protein coding RNA 612	1.61	0.68	4.95E-02	0.050
EMX2OS	EMX2 opposite strand/antisense RNA	-1.59	-0.67	4.96E-02	0.050
LOC10028863	OTU deubiquitinase 7A pseudogene	-1.61	-0.69	4.96E-02	0.050

*FC: Fold Change of Suscpetible sample versus healthy control

Supplementary Table 5: List of genes differentially expressed in breast stroma in susceptible versus healthy breasts

Symbol	geneName	FC*	log2FC	PValue	FDR
MIR6079	microRNA 6079	2.64	1.40	1.3E-05	0.007
ANKRD36BP2	ankyrin repeat domain 36B pseudogene 2	-2.10	-1.07	2.1E-05	0.007
RFTN1	raftlin, lipid raft linker 1	1.39	0.47	5.7E-05	0.013
LOC283710	uncharacterized LOC283710	-3.80	-1.93	1.2E-04	0.013
LRGUK	leucine rich repeats and guanylate kinase domain containing	-2.30	-1.20	1.2E-04	0.013
TBX5	T-box 5	-1.67	-0.74	1.4E-04	0.013
GAS2	growth arrest specific 2	-2.43	-1.28	1.6E-04	0.013
WNT10A	Wnt family member 10A	-2.13	-1.09	1.7E-04	0.013
SLC15A2	solute carrier family 15 member 2	-1.65	-0.72	1.8E-04	0.013
PDK1	pyruvate dehydrogenase kinase 1	-1.53	-0.61	2.0E-04	0.013
FAM46C	family with sequence similarity 46 member C	-2.46	-1.30	2.1E-04	0.013
HIST1H4D	histone cluster 1 H4 family member d	-1.63	-0.71	2.9E-04	0.015
DERL3	derlin 3	-2.14	-1.10	2.9E-04	0.015
BLNK	B-cell linker	-1.43	-0.52	3.0E-04	0.015
PIM2	Pim-2 proto-oncogene, serine/threonine kinase	-1.97	-0.98	3.2E-04	0.015
GPR15	G protein-coupled receptor 15	-6.62	-2.73	3.4E-04	0.015
MAPK10	mitogen-activated protein kinase 10	1.60	0.67	4.1E-04	0.016
SYNJ1	synaptojanin 1	-1.30	-0.38	4.5E-04	0.017
CD38	CD38 molecule	-1.85	-0.89	4.8E-04	0.017
PIN1	peptidylprolyl cis/trans isomerase, NIMA-interacting 1	1.42	0.50	5.5E-04	0.019
SLC17A9	solute carrier family 17 member 9	-1.68	-0.75	6.9E-04	0.020
TCEANC2	transcription elongation factor A N-terminal and central domain containin	-1.31	-0.39	7.7E-04	0.020
DDIT4	DNA damage inducible transcript 4	1.72	0.78	7.8E-04	0.020
FADS3	fatty acid desaturase 3	1.50	0.59	7.8E-04	0.020
C6orf165	NA	-3.02	-1.59	8.0E-04	0.020
LINC01504	long intergenic non-protein coding RNA 1504	-3.04	-1.60	8.2E-04	0.020
TCAP	titin-cap	2.61	1.39	8.6E-04	0.020
LOC100128593	uncharacterized LOC100128593	-2.29	-1.19	9.3E-04	0.020
LOC100506725	uncharacterized LOC100506725	-2.56	-1.36	9.3E-04	0.020
HERPUD1	homocysteine inducible ER protein with ubiquitin like domain 1	-1.32	-0.40	9.4E-04	0.020
SCRN2	secernin 2	1.37	0.45	1.0E-03	0.020
TSSK4	testis specific serine kinase 4	-2.05	-1.03	1.0E-03	0.020
ALDH1L1	aldehyde dehydrogenase 1 family member L1	2.71	1.44	1.0E-03	0.020
TNNC2	troponin C2, fast skeletal type	2.88	1.53	1.1E-03	0.020
GINS2	GINS complex subunit 2	-2.40	-1.27	1.1E-03	0.020
TIAM1	T-cell lymphoma invasion and metastasis 1	-1.35	-0.43	1.1E-03	0.020
SARDH	sarcosine dehydrogenase	-1.51	-0.59	1.1E-03	0.020
NLRP11	NLR family pyrin domain containing 11	-6.95	-2.80	1.2E-03	0.020
TNFRSF18	TNF receptor superfamily member 18	-3.38	-1.76	1.2E-03	0.020
SLC7A10	solute carrier family 7 member 10	3.28	1.71	1.2E-03	0.020
ZBP1	Z-DNA binding protein 1	-1.94	-0.96	1.3E-03	0.020
FAH	fumarylacetoacetate hydrolase	1.82	0.86	1.3E-03	0.020
SLC10A7	solute carrier family 10 member 7	-1.34	-0.42	1.3E-03	0.020
AQP7P1	aquaporin 7 pseudogene 1	2.24	1.16	1.3E-03	0.020
KCNA3	potassium voltage-gated channel subfamily A member 3	-1.83	-0.87	1.4E-03	0.020
ALOX12	arachidonate 12-lipoxygenase, 12S type	-3.71	-1.89	1.4E-03	0.020
OIP5-AS1	OIP5 antisense RNA 1	-1.27	-0.34	1.4E-03	0.020
LOC729737	uncharacterized LOC729737	-1.98	-0.99	1.4E-03	0.020
KANSL1L	KAT8 regulatory NSL complex subunit 1 like	-1.37	-0.45	1.4E-03	0.020
C15orf57	chromosome 15 open reading frame 57	-1.34	-0.42	1.5E-03	0.020
ELOVL5	ELOVL fatty acid elongase 5	1.58	0.66	1.6E-03	0.021
DAPP1	dual adaptor of phosphotyrosine and 3-phosphoinositides 1	-1.98	-0.99	1.6E-03	0.021
GIPC1	GIPC PDZ domain containing family member 1	1.28	0.36	1.7E-03	0.022
MZB1	marginal zone B and B1 cell specific protein	-1.86	-0.90	1.8E-03	0.022
LOC101930071	uncharacterized LOC101930071	1.91	0.94	1.9E-03	0.023
LOC101927318	uncharacterized LOC101927318	-6.40	-2.68	1.9E-03	0.023
IRF4	interferon regulatory factor 4	-1.71	-0.77	1.9E-03	0.023
CYP20A1	cytochrome P450 family 20 subfamily A member 1	-1.28	-0.35	1.9E-03	0.023
LRRC32	leucine rich repeat containing 32	1.34	0.42	2.0E-03	0.023
MAP7D1	MAP7 domain containing 1	1.34	0.42	2.0E-03	0.023
TNNT1	troponin T1, slow skeletal type	4.82	2.27	2.0E-03	0.023
ESRRA	estrogen related receptor alpha	1.33	0.42	2.2E-03	0.024
FAAHP1	fatty acid amide hydrolase pseudogene 1	-3.42	-1.77	2.3E-03	0.024

LY75	lymphocyte antigen 75	-2.19	-1.13	2.3E-03	0.024
DAPK2	death associated protein kinase 2	1.56	0.64	2.4E-03	0.024
KLHL6	kelch like family member 6	-1.55	-0.63	2.4E-03	0.024
TET2	tet methylcytosine dioxygenase 2	-1.21	-0.27	2.5E-03	0.024
PTTG2	pituitary tumor-transforming 2	-2.59	-1.37	2.5E-03	0.024
NPR1	natriuretic peptide receptor 1	1.33	0.41	2.5E-03	0.024
LOC102723766	uncharacterized LOC102723766	-1.99	-0.99	2.5E-03	0.024
PTPRM	protein tyrosine phosphatase, receptor type M	1.29	0.37	2.5E-03	0.024
SNRNP35	small nuclear ribonucleoprotein U11/U12 subunit 35	1.25	0.32	2.6E-03	0.024
RSPH9	radial spoke head 9 homolog	-2.79	-1.48	2.7E-03	0.024
FZD4	frizzled class receptor 4	1.39	0.48	2.7E-03	0.024
COLCA2	colorectal cancer associated 2	1.54	0.62	2.7E-03	0.024
MFSD2A	major facilitator superfamily domain containing 2A	-2.83	-1.50	2.7E-03	0.024
GPR63	G protein-coupled receptor 63	-2.17	-1.12	2.7E-03	0.024
HK2	hexokinase 2	1.99	0.99	2.7E-03	0.024
LINC00648	long intergenic non-protein coding RNA 648	2.89	1.53	2.8E-03	0.024
FRMD4A	FERM domain containing 4A	1.34	0.42	2.8E-03	0.024
NGLY1	N-glycanase 1	-1.24	-0.32	2.9E-03	0.024
TSPAN15	tetraspanin 15	1.47	0.55	2.9E-03	0.024
FCRL5	Fc receptor like 5	-2.00	-1.00	3.1E-03	0.025
SPAG4	sperm associated antigen 4	-2.06	-1.05	3.3E-03	0.027
LINC01230	long intergenic non-protein coding RNA 1230	3.05	1.61	3.4E-03	0.027
P2RY13	purinergic receptor P2Y13	-2.15	-1.10	3.5E-03	0.027
ACADS	acyl-CoA dehydrogenase, C-2 to C-3 short chain	1.47	0.55	3.5E-03	0.027
PHLDA3	pleckstrin homology like domain family A member 3	1.41	0.50	3.5E-03	0.027
MT1X	metallothionein 1X	1.77	0.83	3.6E-03	0.027
CPO	carboxypeptidase O	-2.23	-1.16	3.6E-03	0.027
COL25A1	collagen type XXV alpha 1 chain	1.42	0.51	3.6E-03	0.027
AJAP1	adherens junctions associated protein 1	1.90	0.93	3.7E-03	0.027
UBAC2-AS1	UBAC2 antisense RNA 1	1.83	0.87	3.8E-03	0.028
PLEKHS1	pleckstrin homology domain containing S1	-2.32	-1.21	3.9E-03	0.028
CTNNBIP1	catenin beta interacting protein 1	1.29	0.37	3.9E-03	0.028
MDFI	MyoD family inhibitor	1.83	0.88	3.9E-03	0.028
EAF2	ELL associated factor 2	-1.54	-0.62	4.0E-03	0.028
TLE4	transducin like enhancer of split 4	-1.23	-0.30	4.0E-03	0.028
FAM213A	family with sequence similarity 213 member A	1.59	0.67	4.2E-03	0.029
IL6R	interleukin 6 receptor	-1.34	-0.43	4.2E-03	0.029
SPATS2	spermatogenesis associated serine rich 2	-1.34	-0.42	4.4E-03	0.029
CCR2	C-C motif chemokine receptor 2	-2.16	-1.11	4.4E-03	0.029
ZNF740	zinc finger protein 740	1.19	0.25	4.4E-03	0.029
RACGAP1	Rac GTPase activating protein 1	-1.57	-0.65	4.4E-03	0.029
FABP4	fatty acid binding protein 4	2.30	1.20	4.6E-03	0.030
PHLDB1	pleckstrin homology like domain family B member 1	1.33	0.41	4.7E-03	0.030
OSBPL3	oxysterol binding protein like 3	-1.25	-0.32	4.8E-03	0.030
PLA2G16	phospholipase A2 group XVI	1.69	0.76	4.9E-03	0.030
ETFB	electron transfer flavoprotein beta subunit	1.42	0.51	4.9E-03	0.030
CCSAP	centriole, cilia and spindle associated protein	-1.28	-0.35	5.1E-03	0.030
FGD5P1	FYVE, RhoGEF and PH domain containing 5 pseudogene 1	1.68	0.75	5.2E-03	0.030
APOBEC3F	apolipoprotein B mRNA editing enzyme catalytic subunit 3F	-1.30	-0.38	5.2E-03	0.030
ZNF818P	zinc finger protein 818, pseudogene	-1.47	-0.56	5.3E-03	0.030
RBM17	RNA binding motif protein 17	1.16	0.21	5.3E-03	0.030
AOC3	amine oxidase, copper containing 3	1.67	0.74	5.3E-03	0.030
PABPN1	poly(A) binding protein nuclear 1	1.36	0.45	5.3E-03	0.030
RHOB	ras homolog family member B	1.35	0.43	5.4E-03	0.030
PLGRKT	plasminogen receptor with a C-terminal lysine	1.38	0.46	5.4E-03	0.030
NAT8L	N-acetyltransferase 8 like	2.54	1.35	5.4E-03	0.030
DNALI1	dynein axonemal light intermediate chain 1	1.32	0.40	5.4E-03	0.030
SIM1	single-minded family bHLH transcription factor 1	2.97	1.57	5.4E-03	0.030
LRP4	LDL receptor related protein 4	-1.38	-0.46	5.4E-03	0.030
LINC00324	long intergenic non-protein coding RNA 324	-1.43	-0.52	5.5E-03	0.030
QRICH2	glutamine rich 2	-1.45	-0.54	5.5E-03	0.030
DUSP4	dual specificity phosphatase 4	1.90	0.92	5.5E-03	0.030
DHX34	DEAH-box helicase 34	-1.30	-0.38	5.7E-03	0.031
DCUN1D3	defective in cullin neddylation 1 domain containing 3	1.44	0.53	5.7E-03	0.031
MUC15	mucin 15, cell surface associated	-3.27	-1.71	6.0E-03	0.031
PIM3	Pim-3 proto-oncogene, serine/threonine kinase	1.39	0.47	6.0E-03	0.031

VPS72	vacuolar protein sorting 72 homolog	1.19	0.25	6.0E-03	0.031
TMEM74B	transmembrane protein 74B	1.78	0.84	6.0E-03	0.031
ADIPOQ-AS1	ADIPOQ antisense RNA 1	2.91	1.54	6.1E-03	0.031
TXNDC11	thioredoxin domain containing 11	-1.28	-0.36	6.1E-03	0.031
SLAMF7	SLAM family member 7	-1.76	-0.81	6.1E-03	0.031
ACAA1	acetyl-CoA acyltransferase 1	1.24	0.31	6.2E-03	0.031
APOBEC3B	apolipoprotein B mRNA editing enzyme catalytic subunit 3B	-2.55	-1.35	6.2E-03	0.031
LOC102724927	uncharacterized LOC102724927	-2.15	-1.11	6.4E-03	0.032
ARL4A	ADP ribosylation factor like GTPase 4A	-1.51	-0.59	6.4E-03	0.032
STYXL1	serine/threonine/tyrosine interacting like 1	-1.43	-0.52	6.4E-03	0.032
MRPL41	mitochondrial ribosomal protein L41	1.40	0.49	6.4E-03	0.032
HIST1H2BF	histone cluster 1 H2B family member f	-1.80	-0.85	6.5E-03	0.032
ZNF571	zinc finger protein 571	-1.34	-0.42	7.1E-03	0.034
MTRNR2L8	MT-RNR2-like 8	-1.86	-0.90	7.4E-03	0.034
MRAS	muscle RAS oncogene homolog	1.44	0.52	7.4E-03	0.034
CTNS	cystinosin, lysosomal cystine transporter	-1.27	-0.35	7.4E-03	0.034
PPIL3	peptidylprolyl isomerase like 3	-1.35	-0.43	7.4E-03	0.034
PHKG1	phosphorylase kinase catalytic subunit gamma 1	1.59	0.67	7.5E-03	0.034
IGFBP3	insulin like growth factor binding protein 3	1.37	0.45	7.5E-03	0.034
BRPF1	bromodomain and PHD finger containing 1	1.21	0.27	7.6E-03	0.034
GLDC	glycine decarboxylase	-2.24	-1.16	7.6E-03	0.034
TSTD3	thiosulfate sulfurtransferase (rhodanese)-like domain containing 3	-1.33	-0.41	7.6E-03	0.034
FRG1B	NA	1.26	0.33	7.6E-03	0.034
GPCPD1	glycerophosphocholine phosphodiesterase 1	-1.23	-0.29	7.8E-03	0.034
PEX5	peroxisomal biogenesis factor 5	1.20	0.26	7.8E-03	0.034
BOK	BOK, BCL2 family apoptosis regulator	1.66	0.73	7.9E-03	0.034
SNORD37	small nucleolar RNA, C/D box 37	-1.83	-0.87	7.9E-03	0.034
LOC202181	SUMO interacting motifs containing 1 pseudogene	-1.25	-0.32	8.0E-03	0.034
LHFPL1	lipoma HMGIC fusion partner-like 1	-2.71	-1.44	8.0E-03	0.034
NFKBIA	NFKB inhibitor alpha	1.22	0.28	8.1E-03	0.034
ADCY6	adenylate cyclase 6	1.35	0.43	8.1E-03	0.034
CYB5A	cytochrome b5 type A	1.58	0.66	8.1E-03	0.034
HIST2H2BF	histone cluster 2 H2B family member f	-1.54	-0.63	8.1E-03	0.034
ZNF778	zinc finger protein 778	-1.24	-0.31	8.2E-03	0.034
CDC37	cell division cycle 37	1.23	0.30	8.3E-03	0.034
MYL2	myosin light chain 2	5.55	2.47	8.4E-03	0.034
PPIF	peptidylprolyl isomerase F	1.29	0.36	8.4E-03	0.034
NDUFA4L2	NDUFA4, mitochondrial complex associated like 2	1.44	0.53	8.4E-03	0.034
NOC2L	NOC2 like nucleolar associated transcriptional repressor	1.23	0.29	8.5E-03	0.034
AAMP	angio associated migratory cell protein	1.25	0.32	8.5E-03	0.034
DDAH2	dimethylarginine dimethylaminohydrolase 2	1.35	0.43	8.6E-03	0.035
BOP1	block of proliferation 1	1.44	0.53	8.7E-03	0.035
PCSK4	proprotein convertase subtilisin/kexin type 4	-1.97	-0.98	8.8E-03	0.035
GFOD1	glucose-fructose oxidoreductase domain containing 1	1.43	0.52	8.8E-03	0.035
CDR1	cerebellar degeneration related protein 1	2.36	1.24	8.8E-03	0.035
TFIP11	tuftelin interacting protein 11	1.22	0.29	8.9E-03	0.035
STX2	syntaxin 2	-1.29	-0.37	9.1E-03	0.035
UNG	uracil DNA glycosylase	1.26	0.33	9.1E-03	0.035
SHCBP1	SHC binding and spindle associated 1	2.23	1.15	9.3E-03	0.035
CENPQ	centromere protein Q	-1.51	-0.60	9.3E-03	0.035
CCR5	C-C motif chemokine receptor 5 (gene/pseudogene)	-1.99	-0.99	9.4E-03	0.035
AIFM2	apoptosis inducing factor, mitochondria associated 2	1.52	0.61	9.4E-03	0.035
CXorf21	chromosome X open reading frame 21	-1.88	-0.91	9.4E-03	0.035
PI4K2B	phosphatidylinositol 4-kinase type 2 beta	-1.22	-0.29	9.4E-03	0.035
GRAMD1B	GRAM domain containing 1B	-1.43	-0.51	9.5E-03	0.035
WBP4	WW domain binding protein 4	1.22	0.29	9.5E-03	0.035
AHR	aryl hydrocarbon receptor	-1.29	-0.37	9.6E-03	0.035
PDK2	pyruvate dehydrogenase kinase 2	1.29	0.37	9.7E-03	0.035
ANKRD20A5P	ankyrin repeat domain 20 family member A5, pseudogene	-1.45	-0.53	9.8E-03	0.035
POLH	DNA polymerase eta	-1.27	-0.34	9.9E-03	0.035
CCDC144NL	coiled-coil domain containing 144 family, N-terminal like	2.80	1.49	9.9E-03	0.035
DECR1	2,4-dienoyl-CoA reductase 1, mitochondrial	1.24	0.31	1.0E-02	0.035
FTSJ3	FtsJ homolog 3	1.24	0.31	1.0E-02	0.035
BMS1P20	BMS1, ribosome biogenesis factor pseudogene 20	-1.27	-0.35	1.0E-02	0.035
SLC2A4RG	SLC2A4 regulator	1.20	0.27	1.0E-02	0.035
RMDN3	regulator of microtubule dynamics 3	1.30	0.37	1.0E-02	0.035

CCNJL	cyclin J like	-1.46	-0.55	1.0E-02	0.035
BLCAP	bladder cancer associated protein	1.22	0.28	1.0E-02	0.035
RBP7	retinol binding protein 7	1.70	0.76	1.0E-02	0.035
TLR1	toll like receptor 1	-1.66	-0.73	1.0E-02	0.035
LOC101926975	uncharacterized LOC101926975	2.63	1.39	1.0E-02	0.035
CXorf65	chromosome X open reading frame 65	2.09	1.06	1.0E-02	0.035
RGS3	regulator of G-protein signaling 3	1.34	0.42	1.0E-02	0.035
HN1	hematological and neurological expressed 1	-1.41	-0.50	1.0E-02	0.035
GATA2	GATA binding protein 2	1.29	0.37	1.0E-02	0.035
FAM212B	family with sequence similarity 212 member B	1.36	0.44	1.0E-02	0.035
CARS	cysteinyl-tRNA synthetase	1.19	0.25	1.0E-02	0.035
FAM50A	family with sequence similarity 50 member A	1.21	0.28	1.1E-02	0.035
ANGPT4	angiopoietin 4	2.04	1.03	1.1E-02	0.035
AGPAT2	1-acylglycerol-3-phosphate O-acyltransferase 2	1.95	0.96	1.1E-02	0.035
C20orf27	chromosome 20 open reading frame 27	1.34	0.43	1.1E-02	0.035
PLIN1	perilipin 1	2.46	1.30	1.1E-02	0.035
ANKRD2	ankyrin repeat domain 2	3.25	1.70	1.1E-02	0.035
RBP5	retinol binding protein 5	1.68	0.75	1.1E-02	0.035
E2F2	E2F transcription factor 2	-3.06	-1.61	1.1E-02	0.035
EVI2A	ecotropic viral integration site 2A	-1.83	-0.87	1.1E-02	0.035
LMCD1	LIM and cysteine rich domains 1	1.27	0.34	1.1E-02	0.035
KIAA0125	NA	-1.76	-0.82	1.1E-02	0.035
TLR7	toll like receptor 7	-1.71	-0.78	1.1E-02	0.035
ABCD2	ATP binding cassette subfamily D member 2	1.75	0.81	1.1E-02	0.035
TYRO3	TYRO3 protein tyrosine kinase	1.64	0.72	1.2E-02	0.035
ACO1	aconitase 1	1.40	0.48	1.2E-02	0.035
IKBKG	inhibitor of kappa light polypeptide gene enhancer in B-cells, kinase gam	1.25	0.32	1.2E-02	0.035
ATP1A2	ATPase Na+/K+ transporting subunit alpha 2	2.14	1.10	1.2E-02	0.035
RBP4	retinol binding protein 4	2.90	1.54	1.2E-02	0.035
KIF22	kinesin family member 22	1.26	0.33	1.2E-02	0.035
NBPF1	neuroblastoma breakpoint family member 1	-1.32	-0.40	1.2E-02	0.035
RUNX1-IT1	RUNX1 intronic transcript 1	-1.95	-0.96	1.2E-02	0.035
TSSC4	tumor suppressing subtransferable candidate 4	1.36	0.45	1.2E-02	0.035
TMEM150A	transmembrane protein 150A	-1.32	-0.40	1.2E-02	0.035
LRRC57	leucine rich repeat containing 57	-1.22	-0.29	1.2E-02	0.036
CCDC42B	NA	-2.73	-1.45	1.2E-02	0.036
ZNF215	zinc finger protein 215	-1.75	-0.81	1.2E-02	0.036
GAS2L3	growth arrest specific 2 like 3	-1.54	-0.62	1.2E-02	0.036
DNAL1	dynein axonemal light chain 1	-1.21	-0.28	1.2E-02	0.036
C14orf180	chromosome 14 open reading frame 180	2.53	1.34	1.2E-02	0.036
RALY-AS1	RALY antisense RNA 1	-1.92	-0.94	1.3E-02	0.036
VTRNA1-1	vault RNA 1-1	-1.67	-0.74	1.3E-02	0.036
HINT2	histidine triad nucleotide binding protein 2	1.38	0.47	1.3E-02	0.036
ITGA7	integrin subunit alpha 7	1.63	0.71	1.3E-02	0.036
PRKAB1	protein kinase AMP-activated non-catalytic subunit beta 1	1.20	0.26	1.3E-02	0.036
GBE1	1,4-alpha-glucan branching enzyme 1	1.36	0.44	1.3E-02	0.036
MIR5091	microRNA 5091	1.82	0.86	1.3E-02	0.036
CATSPER2P1	cation channel sperm associated 2 pseudogene 1	1.56	0.64	1.3E-02	0.036
EMX2OS	EMX2 opposite strand/antisense RNA	-1.63	-0.71	1.3E-02	0.036
DTX1	deltex E3 ubiquitin ligase 1	1.46	0.55	1.3E-02	0.036
MEA1	male-enhanced antigen 1	1.27	0.35	1.3E-02	0.036
FERMT1	fermitin family member 1	-1.74	-0.80	1.3E-02	0.036
MYH7	myosin heavy chain 7	6.45	2.69	1.3E-02	0.036
FAM185A	family with sequence similarity 185 member A	-1.22	-0.28	1.3E-02	0.036
CDH5	cadherin 5	1.31	0.39	1.3E-02	0.036
QDPR	quinoid dihydropteridine reductase	1.36	0.44	1.4E-02	0.036
AKR1C3	aldo-keto reductase family 1 member C3	1.45	0.53	1.4E-02	0.036
CYP39A1	cytochrome P450 family 39 subfamily A member 1	-1.50	-0.58	1.4E-02	0.036
HSD17B13	hydroxysteroid 17-beta dehydrogenase 13	2.09	1.06	1.4E-02	0.036
FRMD3	FERM domain containing 3	1.28	0.35	1.4E-02	0.036
P2RY10	purinergic receptor P2Y10	-2.27	-1.18	1.4E-02	0.036
AGAP1-IT1	AGAP1 intronic transcript 1	1.47	0.56	1.4E-02	0.036
NNMT	nicotinamide N-methyltransferase	1.35	0.43	1.4E-02	0.036
PCED1B-AS1	PCED1B antisense RNA 1	-1.68	-0.75	1.4E-02	0.036
ZBTB16	zinc finger and BTB domain containing 16	1.76	0.82	1.4E-02	0.036
PTP4A3	protein tyrosine phosphatase type IVA, member 3	1.72	0.79	1.4E-02	0.036

SLC9C1	solute carrier family 9 member C1	-2.49	-1.31	1.4E-02	0.036
RRH	retinal pigment epithelium-derived rhodopsin homolog	-1.70	-0.77	1.5E-02	0.036
SIGLEC10	sialic acid binding Ig like lectin 10	-1.93	-0.95	1.5E-02	0.036
ZNF205-AS1	ZNF205 antisense RNA 1	1.60	0.68	1.5E-02	0.036
TEKT4P2	tektin 4 pseudogene 2	-1.60	-0.68	1.5E-02	0.036
TSG1	tumor suppressor TSG1	-4.13	-2.05	1.5E-02	0.036
LRRC3	leucine rich repeat containing 3	1.75	0.81	1.5E-02	0.036
LOC441666	zinc finger protein 91 pseudogene	-1.38	-0.47	1.5E-02	0.036
REPIN1	replication initiator 1	1.17	0.23	1.5E-02	0.036
DUSP28	dual specificity phosphatase 28	1.47	0.56	1.5E-02	0.036
STK38	serine/threonine kinase 38	-1.16	-0.21	1.5E-02	0.036
SLC5A4	solute carrier family 5 member 4	1.72	0.78	1.5E-02	0.036
BAALC	brain and acute leukemia, cytoplasmic	1.52	0.61	1.5E-02	0.036
OSGIN2	oxidative stress induced growth inhibitor family member 2	1.32	0.40	1.5E-02	0.036
LAX1	lymphocyte transmembrane adaptor 1	-1.47	-0.56	1.5E-02	0.036
ZGPAT	zinc finger CCCH-type and G-patch domain containing	1.26	0.33	1.5E-02	0.036
GCOM1	GRINL1A complex locus 1	1.50	0.59	1.6E-02	0.036
ST6GALNAC6	ST6 N-acetylgalactosaminide alpha-2,6-sialyltransferase 6	1.18	0.24	1.6E-02	0.036
FAXC	failed axon connections homolog	-1.69	-0.76	1.6E-02	0.036
RPGR	retinitis pigmentosa GTPase regulator	1.38	0.46	1.6E-02	0.036
APOE	apolipoprotein E	1.50	0.59	1.6E-02	0.036
DOC2A	double C2 domain alpha	-3.52	-1.81	1.6E-02	0.036
ZNF812	NA	-2.09	-1.06	1.6E-02	0.036
PDZD2	PDZ domain containing 2	1.46	0.55	1.6E-02	0.036
NCF1B	neutrophil cytosolic factor 1B pseudogene	-1.73	-0.79	1.6E-02	0.036
CD36	CD36 molecule	1.72	0.78	1.6E-02	0.036
KNTC1	kinetochore associated 1	-1.29	-0.36	1.6E-02	0.036
GHR	growth hormone receptor	1.57	0.65	1.6E-02	0.036
SLC2A12	solute carrier family 2 member 12	-1.63	-0.71	1.6E-02	0.036
TMEM37	transmembrane protein 37	2.01	1.01	1.6E-02	0.036
ACVR1C	activin A receptor type 1C	2.35	1.23	1.6E-02	0.036
ADCY3	adenylate cyclase 3	1.23	0.30	1.6E-02	0.036
NT5C2	5'-nucleotidase, cytosolic II	-1.15	-0.20	1.6E-02	0.036
PLA2G2A	phospholipase A2 group IIA	3.16	1.66	1.6E-02	0.036
CSDC2	cold shock domain containing C2	1.76	0.81	1.6E-02	0.036
PTGFR	prostaglandin F receptor	-1.55	-0.63	1.6E-02	0.036
TINAGL1	tubulointerstitial nephritis antigen like 1	1.43	0.52	1.6E-02	0.036
ACSL6	acyl-CoA synthetase long-chain family member 6	-2.01	-1.01	1.7E-02	0.036
GAS5	growth arrest specific 5 (non-protein coding)	-1.30	-0.38	1.7E-02	0.036
NPEPL1	aminopeptidase-like 1	1.40	0.49	1.7E-02	0.036
MIR1304	microRNA 1304	-2.00	-1.00	1.7E-02	0.036
HIPK1-AS1	HIPK1 antisense RNA 1	-1.80	-0.85	1.7E-02	0.036
CHCHD10	coiled-coil-helix-coiled-coil-helix domain containing 10	1.49	0.58	1.7E-02	0.036
SLC46A1	solute carrier family 46 member 1	-1.21	-0.27	1.7E-02	0.036
FRG1	FSHD region gene 1	1.21	0.27	1.7E-02	0.036
PRR11	proline rich 11	-1.69	-0.76	1.7E-02	0.036
ZNF587	zinc finger protein 587	-1.28	-0.36	1.7E-02	0.036
SAA1	serum amyloid A1	3.17	1.66	1.7E-02	0.036
PPP1R12C	protein phosphatase 1 regulatory subunit 12C	1.24	0.31	1.7E-02	0.036
DBNL	drebrin like	1.19	0.26	1.7E-02	0.036
TUBA3D	tubulin alpha 3d	3.24	1.70	1.7E-02	0.036
JAKMIP1	janus kinase and microtubule interacting protein 1	-2.33	-1.22	1.7E-02	0.036
SLC15A4	solute carrier family 15 member 4	-1.18	-0.24	1.7E-02	0.036
JCHAIN	joining chain of multimeric IgA and IgM	-1.89	-0.92	1.7E-02	0.036
FASN	fatty acid synthase	1.88	0.91	1.7E-02	0.036
CASS4	Cas scaffolding protein family member 4	-1.68	-0.75	1.7E-02	0.036
NMUR1	neuromedin U receptor 1	1.90	0.93	1.7E-02	0.036
RETSAT	retinol saturase	1.65	0.72	1.7E-02	0.036
PPARG	peroxisome proliferator activated receptor gamma	1.40	0.49	1.7E-02	0.036
VAMP5	vesicle associated membrane protein 5	1.30	0.38	1.7E-02	0.036
RPS17	ribosomal protein S17	-1.61	-0.69	1.7E-02	0.036
TTC39C-AS1	TTC39C antisense RNA 1	1.78	0.83	1.7E-02	0.036
AKR1C1	aldo-keto reductase family 1 member C1	1.62	0.70	1.7E-02	0.036
GYG2	glycogenin 2	2.25	1.17	1.7E-02	0.036
ABLIM3	actin binding LIM protein family member 3	1.30	0.38	1.8E-02	0.036
PSMB6	proteasome subunit beta 6	1.33	0.41	1.8E-02	0.036

PABPC1L	poly(A) binding protein cytoplasmic 1 like	-1.44	-0.53	1.8E-02	0.036
CEBPA	CCAAT/enhancer binding protein alpha	1.72	0.78	1.8E-02	0.036
RAB3D	RAB3D, member RAS oncogene family	-1.22	-0.28	1.8E-02	0.036
LYPLA2	lysophospholipase II	1.24	0.31	1.8E-02	0.036
LOC389641	uncharacterized LOC389641	-2.15	-1.10	1.8E-02	0.036
LPL	lipoprotein lipase	2.19	1.13	1.8E-02	0.037
TRIM35	tripartite motif containing 35	1.19	0.25	1.8E-02	0.037
PPM1K	protein phosphatase, Mg2+/Mn2+ dependent 1K	-1.25	-0.32	1.8E-02	0.037
BAD	BCL2 associated agonist of cell death	1.24	0.31	1.8E-02	0.037
OTUD7B	OTU deubiquitinase 7B	1.20	0.26	1.8E-02	0.037
SSRP1	structure specific recognition protein 1	1.17	0.22	1.8E-02	0.037
ZNF566	zinc finger protein 566	-1.22	-0.28	1.8E-02	0.037
MGLL	monoglyceride lipase	1.41	0.49	1.9E-02	0.037
LRRC37BP1	leucine rich repeat containing 37B pseudogene 1	-1.21	-0.27	1.9E-02	0.037
CASP4	caspase 4	-1.18	-0.24	1.9E-02	0.037
SNORD95	small nucleolar RNA, C/D box 95	-1.47	-0.55	1.9E-02	0.037
PFKFB2	6-phosphofructo-2-kinase/fructose-2,6-biphosphatase 2	-1.22	-0.29	1.9E-02	0.037
HSH2D	hematopoietic SH2 domain containing	-1.57	-0.65	1.9E-02	0.037
ACACB	acetyl-CoA carboxylase beta	1.58	0.66	1.9E-02	0.037
DNAJA4	DnaJ heat shock protein family (Hsp40) member A4	1.21	0.27	1.9E-02	0.038
TM4SF18	transmembrane 4 L six family member 18	1.28	0.35	1.9E-02	0.038
HEPACAM	hepatic and glial cell adhesion molecule	2.24	1.16	1.9E-02	0.038
NAIP	NLR family apoptosis inhibitory protein	-1.38	-0.47	1.9E-02	0.038
PFKFB1	6-phosphofructo-2-kinase/fructose-2,6-biphosphatase 1	2.17	1.12	1.9E-02	0.038
NCF4	neutrophil cytosolic factor 4	-1.40	-0.48	2.0E-02	0.038
LAT2	linker for activation of T-cells family member 2	-1.59	-0.67	2.0E-02	0.038
C16orf74	chromosome 16 open reading frame 74	-1.65	-0.72	2.0E-02	0.038
C15orf40	chromosome 15 open reading frame 40	-1.30	-0.38	2.0E-02	0.038
AFG3L2	AFG3 like matrix AAA peptidase subunit 2	1.15	0.20	2.0E-02	0.038
IPMK	inositol polyphosphate multikinase	-1.27	-0.35	2.0E-02	0.038
HECTD3	HECT domain E3 ubiquitin protein ligase 3	1.18	0.23	2.0E-02	0.038
ALDH4A1	aldehyde dehydrogenase 4 family member A1	1.55	0.63	2.0E-02	0.038
SDC1	syndecan 1	-1.39	-0.48	2.0E-02	0.038
G0S2	G0/G1 switch 2	2.33	1.22	2.0E-02	0.038
ZNF519	zinc finger protein 519	-1.25	-0.32	2.0E-02	0.038
CCNB1	cyclin B1	-1.36	-0.45	2.0E-02	0.038
ADIPOQ	adiponectin, C1Q and collagen domain containing	2.24	1.16	2.0E-02	0.039
ENDOU	endonuclease, poly(U) specific	1.68	0.75	2.1E-02	0.039
MIR652	microRNA 652	2.42	1.28	2.1E-02	0.039
GALNT15	polypeptide N-acetylgalactosaminyltransferase 15	1.51	0.59	2.1E-02	0.039
ATXN1L	ataxin 1 like	1.16	0.22	2.1E-02	0.039
COL16A1	collagen type XVI alpha 1 chain	1.32	0.40	2.1E-02	0.039
KCTD21-AS1	KCTD21 antisense RNA 1	-1.66	-0.73	2.1E-02	0.039
AKAP8	A-kinase anchoring protein 8	1.19	0.25	2.1E-02	0.039
SLC29A4	solute carrier family 29 member 4	1.96	0.97	2.1E-02	0.039
TUSC5	tumor suppressor candidate 5	2.22	1.15	2.1E-02	0.039
PPP1R1A	protein phosphatase 1 regulatory inhibitor subunit 1A	2.27	1.18	2.1E-02	0.039
VTI1B	vesicle transport through interaction with t-SNAREs 1B	1.26	0.33	2.1E-02	0.039
TK1	thymidine kinase 1	-2.11	-1.08	2.1E-02	0.039
SAP30BP	SAP30 binding protein	1.17	0.22	2.1E-02	0.039
FKBPL	FK506 binding protein like	-1.58	-0.66	2.1E-02	0.039
DPY19L2P3	DPY19L2 pseudogene 3	-2.06	-1.04	2.1E-02	0.039
CRIP3	cysteine rich protein 3	-1.79	-0.84	2.2E-02	0.039
RGPD4	RANBP2-like and GRIP domain containing 4	-1.95	-0.96	2.2E-02	0.039
U2AF1L4	U2 small nuclear RNA auxiliary factor 1 like 4	1.36	0.44	2.2E-02	0.039
PALMD	palmdelphin	1.35	0.43	2.2E-02	0.039
ROBO4	roundabout guidance receptor 4	1.29	0.36	2.2E-02	0.039
GPR176	G protein-coupled receptor 176	1.37	0.45	2.2E-02	0.039
HOXA1	homeobox A1	1.45	0.54	2.2E-02	0.039
MIRLET7I	microRNA let-7i	1.63	0.70	2.2E-02	0.039
UBXN1	UBX domain protein 1	1.18	0.24	2.2E-02	0.039
C2CD2	C2 calcium dependent domain containing 2	1.29	0.36	2.2E-02	0.039
ZBTB41	zinc finger and BTB domain containing 41	-1.18	-0.24	2.2E-02	0.039
POU2AF1	POU class 2 associating factor 1	-1.53	-0.61	2.2E-02	0.039
PDE2A	phosphodiesterase 2A	1.35	0.43	2.3E-02	0.039
KLF15	Kruppel like factor 15	1.68	0.75	2.3E-02	0.039

SNORA30	small nucleolar RNA, H/ACA box 30	-1.85	-0.89	2.3E-02	0.039
LIMCH1	LIM and calponin homology domains 1	1.37	0.46	2.3E-02	0.040
SNORD2	small nucleolar RNA, C/D box 2	-1.47	-0.55	2.3E-02	0.040
SLC7A6OS	solute carrier family 7 member 6 opposite strand	1.18	0.24	2.3E-02	0.040
GPD1	glycerol-3-phosphate dehydrogenase 1	2.27	1.18	2.3E-02	0.040
KLRC1	killer cell lectin like receptor C1	-2.28	-1.19	2.3E-02	0.040
APOL6	apolipoprotein L6	1.29	0.37	2.3E-02	0.040
KCNN2	potassium calcium-activated channel subfamily N member 2	-1.93	-0.95	2.3E-02	0.040
APMAP	adipocyte plasma membrane associated protein	1.28	0.35	2.3E-02	0.040
OR52N4	olfactory receptor family 52 subfamily N member 4 (gene/pseudogene)	2.50	1.32	2.3E-02	0.040
ZNF850	zinc finger protein 850	-1.22	-0.29	2.3E-02	0.040
ZNF383	zinc finger protein 383	-1.20	-0.26	2.3E-02	0.040
MDH1B	malate dehydrogenase 1B	-1.61	-0.69	2.4E-02	0.040
MRPL40	mitochondrial ribosomal protein L40	1.25	0.32	2.4E-02	0.040
SAA2	serum amyloid A2	3.19	1.67	2.4E-02	0.040
KCNIP2	potassium voltage-gated channel interacting protein 2	1.95	0.96	2.4E-02	0.040
CH25H	cholesterol 25-hydroxylase	-1.65	-0.72	2.4E-02	0.040
LOC100506136	uncharacterized LOC100506136	-1.55	-0.63	2.4E-02	0.040
INF2	inverted formin, FH2 and WH2 domain containing	1.26	0.33	2.4E-02	0.040
PUS7L	pseudouridylate synthase 7 like	-1.20	-0.26	2.4E-02	0.040
PLIN2	perilipin 2	1.30	0.38	2.4E-02	0.040
HOXB-AS1	HOXB cluster antisense RNA 1	1.36	0.44	2.4E-02	0.040
DTX3L	deltex E3 ubiquitin ligase 3L	-1.16	-0.21	2.4E-02	0.040
HEY1	hes related family bHLH transcription factor with YRPW motif 1	1.40	0.48	2.4E-02	0.040
MGA	MGA, MAX dimerization protein	-1.14	-0.18	2.4E-02	0.040
NANOS1	nanos C2HC-type zinc finger 1	1.43	0.51	2.4E-02	0.040
NUGGC	nuclear GTPase, germinal center associated	-1.59	-0.67	2.4E-02	0.040
MIR3064	microRNA 3064	-1.47	-0.56	2.4E-02	0.040
ZNF775	zinc finger protein 775	1.31	0.39	2.4E-02	0.040
PRKAR2B	protein kinase cAMP-dependent type II regulatory subunit beta	1.85	0.89	2.5E-02	0.040
IDS	iduronate 2-sulfatase	-1.19	-0.26	2.5E-02	0.040
LOC100506127	putative uncharacterized protein FLJ37770-like	-1.30	-0.37	2.5E-02	0.040
APIP	APAF1 interacting protein	1.28	0.36	2.5E-02	0.040
DHRS3	dehydrogenase/reductase 3	1.32	0.40	2.5E-02	0.040
CEP89	centrosomal protein 89	-1.18	-0.23	2.5E-02	0.040
LRRC47	leucine rich repeat containing 47	1.16	0.21	2.5E-02	0.040
SNAI1	snail family transcriptional repressor 1	1.52	0.61	2.5E-02	0.040
FAM89A	family with sequence similarity 89 member A	1.52	0.60	2.5E-02	0.040
STON1	stonin 1	1.24	0.31	2.5E-02	0.040
CEP97	centrosomal protein 97	-1.20	-0.27	2.5E-02	0.040
SEL1L3	SEL1L family member 3	-1.30	-0.38	2.5E-02	0.040
ORMDL3	ORMDL sphingolipid biosynthesis regulator 3	1.31	0.39	2.5E-02	0.040
LYRM7	LYR motif containing 7	-1.22	-0.28	2.6E-02	0.040
NIPSNAP3B	nipsnap homolog 3B	1.38	0.46	2.6E-02	0.040
SNORD36C	small nucleolar RNA, C/D box 36C	-1.52	-0.61	2.6E-02	0.040
PRICKLE2-AS3	PRICKLE2 antisense RNA 3	-1.62	-0.69	2.6E-02	0.040
LOC101929066	uncharacterized LOC101929066	-1.76	-0.82	2.6E-02	0.040
PTGER4	prostaglandin E receptor 4	-1.35	-0.43	2.6E-02	0.040
METTL8	methyltransferase like 8	-1.19	-0.25	2.6E-02	0.040
NOD2	nucleotide binding oligomerization domain containing 2	-1.70	-0.76	2.6E-02	0.040
MIR590	microRNA 590	1.61	0.69	2.6E-02	0.040
TSPAN6	tetraspanin 6	1.26	0.33	2.6E-02	0.040
C2orf44	NA	-1.29	-0.37	2.6E-02	0.040
ITPK1	inositol-tetrakisphosphate 1-kinase	1.27	0.34	2.6E-02	0.040
SNORD59A	small nucleolar RNA, C/D box 59A	-1.71	-0.78	2.7E-02	0.040
FABP5	fatty acid binding protein 5	1.50	0.59	2.7E-02	0.040
DNAJB12	DnaJ heat shock protein family (Hsp40) member B12	1.14	0.19	2.7E-02	0.040
TOX2	TOX high mobility group box family member 2	1.47	0.55	2.7E-02	0.040
SLC19A3	solute carrier family 19 member 3	2.53	1.34	2.7E-02	0.041
NFKBIL1	NFKB inhibitor like 1	1.27	0.34	2.7E-02	0.041
ANO3	anoctamin 3	1.75	0.81	2.7E-02	0.041
PMEPA1	prostate transmembrane protein, androgen induced 1	1.39	0.47	2.7E-02	0.041
RGS16	regulator of G-protein signaling 16	1.47	0.55	2.7E-02	0.041
RAB36	RAB36, member RAS oncogene family	-1.42	-0.50	2.7E-02	0.041
KMT2E-AS1	KMT2E antisense RNA 1 (head to head)	1.23	0.30	2.7E-02	0.041
APOB	apolipoprotein B	2.55	1.35	2.7E-02	0.041

MTO1	mitochondrial tRNA translation optimization 1	-1.20	-0.27	2.7E-02	0.041
AMIGO1	adhesion molecule with Ig like domain 1	1.28	0.35	2.7E-02	0.041
FAM210A	family with sequence similarity 210 member A	-1.23	-0.30	2.7E-02	0.041
TMEM88	transmembrane protein 88	1.36	0.45	2.8E-02	0.041
MEOX1	mesenchyme homeobox 1	1.40	0.49	2.8E-02	0.041
LMNB1	lamin B1	-1.43	-0.52	2.8E-02	0.041
ZNF101	zinc finger protein 101	-1.32	-0.40	2.8E-02	0.041
LOC101928324	uncharacterized LOC101928324	-1.57	-0.65	2.8E-02	0.041
SCARB1	scavenger receptor class B member 1	1.32	0.40	2.8E-02	0.041
ZNF581	zinc finger protein 581	-1.29	-0.37	2.8E-02	0.041
SCARNA1	small Cajal body-specific RNA 1	-1.43	-0.52	2.8E-02	0.041
CD300LG	CD300 molecule like family member g	1.67	0.74	2.8E-02	0.041
ZNF418	zinc finger protein 418	1.30	0.38	2.8E-02	0.041
GRM7	glutamate metabotropic receptor 7	-2.03	-1.02	2.8E-02	0.041
RIC8B	RIC8 guanine nucleotide exchange factor B	-1.17	-0.23	2.8E-02	0.041
PDRG1	p53 and DNA damage regulated 1	1.26	0.33	2.9E-02	0.041
AKAP1	A-kinase anchoring protein 1	1.29	0.37	2.9E-02	0.041
LOC100130357	uncharacterized LOC100130357	-1.47	-0.56	2.9E-02	0.041
LOC441155	zinc finger CCCH-type domain-containing-like	1.63	0.70	2.9E-02	0.041
MIR7641-2	microRNA 7641-2	-1.55	-0.63	2.9E-02	0.041
PALM	paralemmin	1.26	0.33	2.9E-02	0.041
ZNF513	zinc finger protein 513	1.36	0.44	2.9E-02	0.041
RCAN3	RCAN family member 3	-1.29	-0.37	2.9E-02	0.041
MLXIPL	MLX interacting protein like	2.14	1.10	2.9E-02	0.041
TMEM259	transmembrane protein 259	1.16	0.22	2.9E-02	0.042
MIR223	microRNA 223	-2.43	-1.28	2.9E-02	0.042
ECM2	extracellular matrix protein 2	1.35	0.43	2.9E-02	0.042
PNPLA2	patatin like phospholipase domain containing 2	1.61	0.69	3.0E-02	0.042
SCG2	secretogranin II	2.34	1.23	3.0E-02	0.042
SEMA3G	semaphorin 3G	1.41	0.49	3.0E-02	0.042
VWF	von Willebrand factor	1.27	0.35	3.0E-02	0.042
APOBEC3G	apolipoprotein B mRNA editing enzyme catalytic subunit 3G	-1.31	-0.38	3.0E-02	0.042
CCDC12	coiled-coil domain containing 12	1.18	0.24	3.0E-02	0.042
EPHX1	epoxide hydrolase 1	1.29	0.37	3.0E-02	0.042
ADCK3	NA	1.40	0.49	3.0E-02	0.042
EPAS1	endothelial PAS domain protein 1	1.30	0.38	3.0E-02	0.042
GFM2	G elongation factor mitochondrial 2	-1.18	-0.24	3.0E-02	0.042
C19orf12	chromosome 19 open reading frame 12	1.25	0.33	3.1E-02	0.042
CUTC	cutC copper transporter	1.16	0.21	3.1E-02	0.042
RXRB	retinoid X receptor beta	1.19	0.26	3.1E-02	0.042
CEACAM21	carcinoembryonic antigen related cell adhesion molecule 21	-1.71	-0.77	3.1E-02	0.042
CWC25	CWC25 spliceosome associated protein homolog	1.14	0.19	3.1E-02	0.042
CYP4F22	cytochrome P450 family 4 subfamily F member 22	2.21	1.14	3.1E-02	0.042
SRSF4	serine and arginine rich splicing factor 4	1.16	0.22	3.1E-02	0.042
ETFDH	electron transfer flavoprotein dehydrogenase	1.26	0.34	3.1E-02	0.042
ASPH	aspartate beta-hydroxylase	1.25	0.32	3.1E-02	0.042
NR2F6	nuclear receptor subfamily 2 group F member 6	1.28	0.35	3.1E-02	0.042
SLC25A20	solute carrier family 25 member 20	1.40	0.49	3.1E-02	0.042
TANC1	tetratricopeptide repeat, ankyrin repeat and coiled-coil containing 1	1.22	0.28	3.1E-02	0.042
RAD1	RAD1 checkpoint DNA exonuclease	-1.16	-0.22	3.1E-02	0.042
C7orf31	chromosome 7 open reading frame 31	-1.32	-0.40	3.1E-02	0.042
PRDX6	peroxiredoxin 6	1.32	0.40	3.2E-02	0.042
SQSTM1	sequestosome 1	1.16	0.22	3.2E-02	0.042
POC1B	POC1 centriolar protein B	-1.24	-0.31	3.2E-02	0.042
PDE8B	phosphodiesterase 8B	1.46	0.54	3.2E-02	0.042
ZNF821	zinc finger protein 821	1.31	0.39	3.2E-02	0.042
GNAI1	G protein subunit alpha i1	1.41	0.49	3.2E-02	0.042
VAX2	ventral anterior homeobox 2	1.57	0.65	3.2E-02	0.043
ALOX5	arachidonate 5-lipoxygenase	-1.38	-0.46	3.2E-02	0.043
SLC46A3	solute carrier family 46 member 3	-1.28	-0.35	3.2E-02	0.043
CD8A	CD8a molecule	-1.72	-0.78	3.2E-02	0.043
PIK3CG	phosphatidylinositol-4,5-bisphosphate 3-kinase catalytic subunit gamma	-1.38	-0.46	3.2E-02	0.043
HARS	histidyl-tRNA synthetase	1.15	0.20	3.2E-02	0.043
CYP4V2	cytochrome P450 family 4 subfamily V member 2	-1.24	-0.31	3.3E-02	0.043
HOXC4	homeobox C4	-1.39	-0.48	3.3E-02	0.043
PSTK	phosphoseryl-tRNA kinase	-1.43	-0.52	3.3E-02	0.043

CDKN2B	cyclin dependent kinase inhibitor 2B	1.62	0.69	3.3E-02	0.043
MX2	MX dynamin like GTPase 2	-1.27	-0.35	3.3E-02	0.043
AMOTL2	angiomotin like 2	1.32	0.40	3.3E-02	0.043
TMEM69	transmembrane protein 69	-1.18	-0.24	3.3E-02	0.043
SLC27A5	solute carrier family 27 member 5	1.32	0.40	3.3E-02	0.043
NSUN4	NOP2/Sun RNA methyltransferase family member 4	-1.20	-0.26	3.4E-02	0.043
MARC1	mitochondrial amidoxime reducing component 1	1.83	0.87	3.4E-02	0.043
GNAO1	G protein subunit alpha o1	-1.50	-0.59	3.4E-02	0.043
NOSTRIN	nitric oxide synthase trafficking	1.35	0.43	3.4E-02	0.043
KCNQ1	potassium voltage-gated channel subfamily Q member 1	-1.38	-0.47	3.4E-02	0.043
STRN4	striatin 4	1.14	0.19	3.4E-02	0.043
PDGFB	platelet derived growth factor subunit B	1.34	0.42	3.4E-02	0.043
MAOA	monoamine oxidase A	1.62	0.70	3.4E-02	0.043
DMRT3	doublesex and mab-3 related transcription factor 3	1.54	0.62	3.4E-02	0.044
ILVBL	ilvB acetolactate synthase like	1.20	0.26	3.4E-02	0.044
CXCL3	C-X-C motif chemokine ligand 3	-1.94	-0.95	3.4E-02	0.044
LINC00996	long intergenic non-protein coding RNA 996	-1.63	-0.70	3.5E-02	0.044
C1orf35	chromosome 1 open reading frame 35	1.25	0.32	3.5E-02	0.044
LINS	NA	-1.25	-0.33	3.5E-02	0.044
CCNB1IP1	cyclin B1 interacting protein 1	-1.23	-0.30	3.5E-02	0.044
LOC100505549	uncharacterized LOC100505549	-1.34	-0.43	3.5E-02	0.044
MMAB	methylmalonic aciduria (cobalamin deficiency) cblB type	1.24	0.31	3.5E-02	0.044
LOC100506474	uncharacterized LOC100506474	1.68	0.75	3.5E-02	0.044
ZNF561	zinc finger protein 561	-1.19	-0.25	3.5E-02	0.044
PDCD2L	programmed cell death 2 like	1.24	0.31	3.5E-02	0.044
PHLDB2	pleckstrin homology like domain family B member 2	1.27	0.35	3.5E-02	0.044
CAPN2	calpain 2	1.16	0.22	3.6E-02	0.044
DHRS4L2	dehydrogenase/reductase 4 like 2	1.27	0.34	3.6E-02	0.044
LOC101928370	uncharacterized LOC101928370	1.43	0.51	3.6E-02	0.044
MMD	monocyte to macrophage differentiation associated	1.78	0.83	3.6E-02	0.044
CPNE5	copine 5	-1.39	-0.48	3.6E-02	0.044
LGI4	leucine rich repeat LGI family member 4	1.42	0.51	3.6E-02	0.044
EMC6	ER membrane protein complex subunit 6	1.36	0.45	3.6E-02	0.045
STBD1	starch binding domain 1	1.84	0.88	3.6E-02	0.045
SNORD56	small nucleolar RNA, C/D box 56	-1.70	-0.77	3.6E-02	0.045
FPGT	fucose-1-phosphate guanylyltransferase	-1.28	-0.36	3.7E-02	0.045
RGS14	regulator of G-protein signaling 14	-1.31	-0.39	3.7E-02	0.045
ESYT1	extended synaptotagmin 1	1.25	0.32	3.7E-02	0.045
CARD8-AS1	CARD8 antisense RNA 1	-1.40	-0.48	3.7E-02	0.045
APAF1	apoptotic peptidase activating factor 1	-1.25	-0.32	3.7E-02	0.045
CS	citrate synthase	1.29	0.36	3.7E-02	0.045
COL13A1	collagen type XIII alpha 1 chain	1.49	0.57	3.7E-02	0.045
AOC4P	amine oxidase, copper containing 4, pseudogene	1.85	0.89	3.7E-02	0.045
FAM86JP	family with sequence similarity 86, member A pseudogene	-1.94	-0.96	3.7E-02	0.045
NOP9	NOP9 nucleolar protein	1.16	0.21	3.7E-02	0.045
CPT1C	carnitine palmitoyltransferase 1C	-1.40	-0.48	3.8E-02	0.045
CAV1	caveolin 1	1.27	0.34	3.8E-02	0.045
LOC730183	uncharacterized LOC730183	1.57	0.66	3.8E-02	0.045
GJA4	gap junction protein alpha 4	1.42	0.51	3.8E-02	0.045
RBM45	RNA binding motif protein 45	-1.22	-0.29	3.8E-02	0.045
PTPRVP	protein tyrosine phosphatase, receptor type V, pseudogene	-1.69	-0.76	3.8E-02	0.045
ZNF219	zinc finger protein 219	1.26	0.33	3.8E-02	0.045
FLJ42627	uncharacterized LOC645644	-1.52	-0.60	3.8E-02	0.045
COL4A2	collagen type IV alpha 2 chain	1.36	0.44	3.8E-02	0.045
LRRTM2	leucine rich repeat transmembrane neuronal 2	1.42	0.51	3.9E-02	0.045
CDK9	cyclin dependent kinase 9	1.19	0.25	3.9E-02	0.045
LOC101928489	uncharacterized LOC101928489	-1.69	-0.76	3.9E-02	0.045
ZNF225	zinc finger protein 225	-1.15	-0.20	3.9E-02	0.045
SNORD59B	small nucleolar RNA, C/D box 59B	-1.72	-0.78	3.9E-02	0.045
MIR1254-1	microRNA 1254-1	-1.69	-0.76	3.9E-02	0.045
KLHL3	kelch like family member 3	1.25	0.32	3.9E-02	0.045
ZNF235	zinc finger protein 235	-1.20	-0.26	3.9E-02	0.045
PFAS	phosphoribosylformylglycinamidine synthase	1.21	0.27	3.9E-02	0.046
PHF20	PHD finger protein 20	-1.14	-0.19	4.0E-02	0.046
GPT2	glutamic--pyruvic transaminase 2	1.57	0.65	4.0E-02	0.046
LINC01232	long intergenic non-protein coding RNA 1232	-1.55	-0.63	4.0E-02	0.046

RNU6-2	RNA, U6 small nuclear 2	-1.37	-0.45	4.0E-02	0.046
CCDC120	coiled-coil domain containing 120	-1.36	-0.44	4.0E-02	0.046
ZRANB2-AS1	ZRANB2 antisense RNA 1	1.63	0.71	4.0E-02	0.046
RAB26	RAB26, member RAS oncogene family	-2.00	-1.00	4.0E-02	0.046
EID2B	EP300 interacting inhibitor of differentiation 2B	-1.31	-0.39	4.1E-02	0.047
PTH2R	parathyroid hormone 2 receptor	1.66	0.73	4.1E-02	0.047
BTN2A3P	butyrophilin subfamily 2 member A3, pseudogene	-1.29	-0.36	4.1E-02	0.047
SHISA3	shisa family member 3	-1.75	-0.81	4.1E-02	0.047
VPS8	VPS8, CORVET complex subunit	-1.16	-0.22	4.1E-02	0.047
CTIF	cap binding complex dependent translation initiation factor	1.25	0.32	4.1E-02	0.047
TNIP1	TNFAIP3 interacting protein 1	1.19	0.25	4.1E-02	0.047
SWSAP1	SWIM-type zinc finger 7 associated protein 1	-1.47	-0.55	4.1E-02	0.047
TAF11	TATA-box binding protein associated factor 11	1.22	0.29	4.1E-02	0.047
BAIAP2L2	BAI1 associated protein 2 like 2	1.53	0.61	4.2E-02	0.047
SPN	sialophorin	-1.52	-0.61	4.2E-02	0.047
AQP7P3	aquaporin 7 pseudogene 3	2.10	1.07	4.2E-02	0.047
LZTS2	leucine zipper tumor suppressor 2	1.17	0.23	4.2E-02	0.047
WDR60	WD repeat domain 60	1.17	0.22	4.2E-02	0.047
BTNL9	butyrophilin like 9	1.63	0.70	4.2E-02	0.047
ANO2	anoctamin 2	1.40	0.49	4.2E-02	0.047
CA4	carbonic anhydrase 4	1.79	0.84	4.2E-02	0.047
PRKRIP1	PRKR interacting protein 1 (IL11 inducible)	1.26	0.33	4.2E-02	0.047
AQP7	aquaporin 7	1.89	0.92	4.2E-02	0.047
CHKB-AS1	CHKB antisense RNA 1 (head to head)	-1.35	-0.43	4.2E-02	0.047
CDC23	cell division cycle 23	-1.15	-0.21	4.2E-02	0.047
CYTIP	cytohesin 1 interacting protein	-1.59	-0.67	4.2E-02	0.047
SPSB1	splA/ryanodine receptor domain and SOCS box containing 1	1.36	0.45	4.2E-02	0.047
MEI1	meiotic double-stranded break formation protein 1	-1.50	-0.59	4.3E-02	0.047
HP09053	uncharacterized LOC101929357	-2.08	-1.06	4.3E-02	0.047
TXNL4B	thioredoxin like 4B	-1.21	-0.28	4.3E-02	0.047
XCR1	X-C motif chemokine receptor 1	-2.57	-1.36	4.3E-02	0.047
AKR1E2	aldo-keto reductase family 1 member E2	1.28	0.35	4.3E-02	0.047
MKNK2	MAP kinase interacting serine/threonine kinase 2	1.18	0.23	4.3E-02	0.047
CCDC168	coiled-coil domain containing 168	-1.71	-0.77	4.3E-02	0.047
RAB3IP	RAB3A interacting protein	-1.38	-0.46	4.3E-02	0.047
MAP3K6	mitogen-activated protein kinase kinase kinase 6	1.26	0.33	4.3E-02	0.047
C17orf100	chromosome 17 open reading frame 100	-1.50	-0.58	4.3E-02	0.047
CFAP45	cilia and flagella associated protein 45	1.50	0.59	4.3E-02	0.047
CDO1	cysteine dioxygenase type 1	1.46	0.55	4.3E-02	0.047
FAM96A	family with sequence similarity 96 member A	-1.17	-0.23	4.4E-02	0.047
PMS1	PMS1 homolog 1, mismatch repair system component	-1.19	-0.25	4.4E-02	0.047
PHOSPHO2	phosphatase, orphan 2	-1.37	-0.46	4.4E-02	0.047
TIGD4	tigger transposable element derived 4	-1.57	-0.65	4.4E-02	0.047
ZNF557	zinc finger protein 557	-1.14	-0.19	4.4E-02	0.047
SLC45A4	solute carrier family 45 member 4	-1.20	-0.26	4.4E-02	0.047
ADAT1	adenosine deaminase, tRNA specific 1	-1.18	-0.24	4.4E-02	0.047
GSE1	Gse1 coiled-coil protein	1.18	0.24	4.4E-02	0.048
INIP	INTS3 and NABP interacting protein	-1.22	-0.29	4.4E-02	0.048
MIR7161	microRNA 7161	1.93	0.95	4.4E-02	0.048
MIR5586	microRNA 5586	-1.88	-0.91	4.5E-02	0.048
LOC101927550	uncharacterized LOC101927550	-1.35	-0.43	4.5E-02	0.048
KMO	kynurenine 3-monooxygenase	-1.73	-0.79	4.5E-02	0.048
FNDC3A	fibronectin type III domain containing 3A	-1.12	-0.17	4.5E-02	0.048
RDH16	retinol dehydrogenase 16 (all-trans)	1.50	0.59	4.5E-02	0.048
LIPE	lipase E, hormone sensitive type	1.96	0.97	4.5E-02	0.048
LOC101926960	uncharacterized LOC101926960	2.24	1.16	4.5E-02	0.048
SOX2-OT	SOX2 overlapping transcript	-1.88	-0.91	4.5E-02	0.048
PTRF	polymerase I and transcript release factor	1.26	0.33	4.5E-02	0.048
BIN2	bridging integrator 2	-1.45	-0.54	4.6E-02	0.048
NFU1	NFU1 iron-sulfur cluster scaffold	1.24	0.31	4.6E-02	0.048
RSRP1	arginine and serine rich protein 1	-1.17	-0.23	4.6E-02	0.048
KANK3	KN motif and ankyrin repeat domains 3	1.37	0.45	4.6E-02	0.048
PTTG1	pituitary tumor-transforming 1	-1.75	-0.81	4.6E-02	0.048
GCAT	glycine C-acetyltransferase	-1.50	-0.58	4.6E-02	0.048
AGAP5	ArfGAP with GTPase domain, ankyrin repeat and PH domain 5	-1.26	-0.34	4.6E-02	0.048
ISCA2	iron-sulfur cluster assembly 2	1.25	0.32	4.6E-02	0.048

LINC00426	long intergenic non-protein coding RNA 426	-1.82	-0.87	4.6E-02	0.048
EDEM1	ER degradation enhancing alpha-mannosidase like protein 1	-1.12	-0.17	4.6E-02	0.048
COPRS	coordinator of PRMT5 and differentiation stimulator	1.21	0.28	4.6E-02	0.048
FGF2	fibroblast growth factor 2	1.26	0.34	4.6E-02	0.048
PMM1	phosphomannomutase 1	1.26	0.33	4.7E-02	0.048
SIAH2	siah E3 ubiquitin protein ligase 2	-1.24	-0.31	4.7E-02	0.048
CLDND2	claudin domain containing 2	1.62	0.70	4.7E-02	0.048
NUDT6	nudix hydrolase 6	1.24	0.31	4.7E-02	0.048
C1QTNF9B-AS	C1QTNF9B antisense RNA 1	-1.80	-0.85	4.7E-02	0.049
ADGRF5	adhesion G protein-coupled receptor F5	1.27	0.35	4.7E-02	0.049
ANGPTL4	angiopoietin like 4	1.90	0.93	4.7E-02	0.049
SKAP1	src kinase associated phosphoprotein 1	-1.42	-0.51	4.7E-02	0.049
MIR623	microRNA 623	-1.99	-1.00	4.8E-02	0.049
GALR1	galanin receptor 1	2.21	1.15	4.8E-02	0.049
SNCG	synuclein gamma	1.47	0.56	4.8E-02	0.049
MIR126	microRNA 126	1.90	0.92	4.8E-02	0.049
SIAE	sialic acid acetylesterase	1.18	0.24	4.8E-02	0.049
MIR6809	microRNA 6809	1.46	0.55	4.8E-02	0.049
TMED1	transmembrane p24 trafficking protein 1	1.28	0.35	4.8E-02	0.049
KLHL5	kelch like family member 5	1.13	0.18	4.8E-02	0.049
ZNF266	zinc finger protein 266	-1.27	-0.35	4.8E-02	0.049
ADIPOR2	adiponectin receptor 2	1.18	0.23	4.9E-02	0.049
DRAP1	DR1 associated protein 1	1.28	0.35	4.9E-02	0.049
FASTKD3	FAST kinase domains 3	-1.24	-0.31	4.9E-02	0.049
RILP	Rab interacting lysosomal protein	1.23	0.30	4.9E-02	0.049
SNORD96A	small nucleolar RNA, C/D box 96A	-1.44	-0.52	5.0E-02	0.050
PTS	6-pyruvoyltetrahydropterin synthase	1.25	0.32	5.0E-02	0.050
HIST1H4E	histone cluster 1 H4 family member e	-1.22	-0.29	5.0E-02	0.050

* FC: Fold Change

Supplementary Table 6: List of genes differentially expressed in breast adipose tissue in susceptible versus healthy breasts.

Symbol	geneName	FC	log2FC	PValue	FDR
LOC101929680	uncharacterized LOC101929680	6.76	2.76	9.3E-06	0.001
C19orf35	chromosome 19 open reading frame 35	-6.25	-2.64	3.6E-04	0.013
LINC00667	long intergenic non-protein coding RNA 667	1.71	0.78	4.2E-04	0.013
ZKSCAN2	zinc finger with KRAB and SCAN domains 2	1.46	0.54	5.4E-04	0.013
CPA4	carboxypeptidase A4	-6.15	-2.62	5.8E-04	0.013
FCHO1	FCH domain only 1	-2.67	-1.41	5.8E-04	0.013
IDH2	isocitrate dehydrogenase (NADP(+)) 2, mitochondrial	1.67	0.74	8.3E-04	0.013
CES4A	carboxylesterase 4A	-2.09	-1.06	8.3E-04	0.013
RIMS2	regulating synaptic membrane exocytosis 2	9.54	3.25	8.4E-04	0.013
SPTB	spectrin beta, erythrocytic	2.50	1.32	8.8E-04	0.013
CAMK2B	calcium/calmodulin dependent protein kinase II beta	-4.86	-2.28	1.0E-03	0.014
NTS	neurotensin	7.33	2.87	1.6E-03	0.020
BRIP1	BRCA1 interacting protein C-terminal helicase 1	2.07	1.05	2.1E-03	0.020
ETV2	ETS variant 2	3.64	1.87	2.1E-03	0.020
SNORD85	NA	-2.16	-1.11	2.2E-03	0.020
FAM46C	family with sequence similarity 46 member C	-2.42	-1.28	2.5E-03	0.020
GADD45B	growth arrest and DNA damage inducible beta	1.64	0.72	2.6E-03	0.020
ASGR1	asialoglycoprotein receptor 1	-4.19	-2.07	2.7E-03	0.020
SNORD58A	small nucleolar RNA, C/D box 58A	-1.75	-0.81	2.7E-03	0.020
ZDHHC11	zinc finger DHHC-type containing 11	-2.09	-1.06	2.8E-03	0.020
MAMLD1	mastermind like domain containing 1	1.44	0.52	3.1E-03	0.022
APOBEC3G	apolipoprotein B mRNA editing enzyme catalytic subun	-1.73	-0.79	3.5E-03	0.023
SLC6A13	solute carrier family 6 member 13	3.67	1.88	3.6E-03	0.023
KLF9	Kruppel like factor 9	1.53	0.61	3.9E-03	0.023
UCP3	uncoupling protein 3	-2.45	-1.29	3.9E-03	0.023
DPYSL4	dihydropyrimidinase like 4	-2.83	-1.50	4.1E-03	0.023
SCP2	sterol carrier protein 2	1.51	0.59	4.2E-03	0.023
FKBP5	FK506 binding protein 5	1.94	0.96	4.4E-03	0.023
RTN1	reticulon 1	1.69	0.76	5.0E-03	0.025
ADCY10	adenylate cyclase 10, soluble	3.48	1.80	5.1E-03	0.025
PPIL6	peptidylprolyl isomerase like 6	-1.72	-0.78	5.3E-03	0.025
TSNAXIP1	translin associated factor X interacting protein 1	-2.53	-1.34	5.5E-03	0.025
TMEM91	transmembrane protein 91	-1.74	-0.80	5.6E-03	0.025
LOC100507639	uncharacterized LOC100507639	4.32	2.11	5.8E-03	0.025
AP4M1	adaptor related protein complex 4 mu 1 subunit	-1.54	-0.62	6.0E-03	0.025
LINC00324	long intergenic non-protein coding RNA 324	-1.83	-0.87	6.7E-03	0.027
GRIA1	glutamate ionotropic receptor AMPA type subunit 1	2.68	1.42	7.6E-03	0.030
BLNK	B-cell linker	-1.79	-0.84	7.9E-03	0.030
LOC105376360	uncharacterized LOC105376360	5.37	2.42	8.1E-03	0.030
SLC22A14	solute carrier family 22 member 14	-4.65	-2.22	8.1E-03	0.030
GALNT13	polypeptide N-acetylgalactosaminyltransferase 13	2.15	1.11	8.2E-03	0.030
CYP26C1	cytochrome P450 family 26 subfamily C member 1	-4.50	-2.17	8.7E-03	0.031
CERS6	ceramide synthase 6	1.32	0.40	9.1E-03	0.031
KLHL6	kelch like family member 6	-1.55	-0.63	9.5E-03	0.031
MIR5690	microRNA 5690	3.51	1.81	9.5E-03	0.031
NFKBIA	NFKB inhibitor alpha	1.40	0.48	1.0E-02	0.031
LIPG	lipase G, endothelial type	-5.31	-2.41	1.0E-02	0.031
C8orf89	chromosome 8 open reading frame 89	-3.70	-1.89	1.0E-02	0.031
CHURC1	churchill domain containing 1	1.33	0.41	1.0E-02	0.031
KCTD14	potassium channel tetramerization domain containing 1	-6.16	-2.62	1.1E-02	0.031
SLC27A2	solute carrier family 27 member 2	3.84	1.94	1.1E-02	0.031
FIGN	fidgetin, microtubule severing factor	1.65	0.72	1.1E-02	0.031

MFI2-AS1	MFI2 antisense RNA 1	-1.92	-0.94	1.1E-02	0.031
CA13	carbonic anhydrase 13	-2.09	-1.07	1.1E-02	0.031
LIFR	leukemia inhibitory factor receptor alpha	1.43	0.52	1.2E-02	0.031
MT1X	metallothionein 1X	2.00	1.00	1.2E-02	0.031
USP6NL	USP6 N-terminal like	1.44	0.53	1.2E-02	0.031
FOXN2	forkhead box N2	1.47	0.56	1.3E-02	0.032
DIRC3	disrupted in renal carcinoma 3	1.51	0.59	1.3E-02	0.034
ADRB3	adrenoceptor beta 3	4.74	2.24	1.4E-02	0.036
CD27	CD27 molecule	-3.48	-1.80	1.5E-02	0.036
ZNF677	zinc finger protein 677	1.38	0.46	1.6E-02	0.037
LINC00312	long intergenic non-protein coding RNA 312	1.59	0.67	1.6E-02	0.038
GLUL	glutamate-ammonia ligase	1.91	0.93	1.6E-02	0.038
LINC00562	long intergenic non-protein coding RNA 562	1.97	0.98	1.7E-02	0.038
TMEM132C	transmembrane protein 132C	1.47	0.55	1.7E-02	0.038
AMIGO1	adhesion molecule with Ig like domain 1	1.56	0.64	1.7E-02	0.038
SLC16A5	solute carrier family 16 member 5	-1.58	-0.66	1.8E-02	0.038
SMOC1	SPARC related modular calcium binding 1	2.47	1.30	1.8E-02	0.038
CCDC150	coiled-coil domain containing 150	-1.82	-0.86	1.8E-02	0.038
SLC38A3	solute carrier family 38 member 3	3.31	1.73	1.9E-02	0.038
TSGA10	testis specific 10	1.53	0.62	1.9E-02	0.038
THRB	thyroid hormone receptor beta	1.34	0.42	1.9E-02	0.038
SNORA16B	small nucleolar RNA, H/ACA box 16B	-2.51	-1.33	1.9E-02	0.038
ACVR1C	activin A receptor type 1C	2.06	1.04	1.9E-02	0.038
MIR921	microRNA 921	-3.51	-1.81	2.0E-02	0.038
DNAH6	dynein axonemal heavy chain 6	2.10	1.07	2.0E-02	0.038
SENP3	SUMO1/sentrin/SMT3 specific peptidase 3	2.07	1.05	2.0E-02	0.038
BPIFB2	BPI fold containing family B member 2	4.92	2.30	2.1E-02	0.039
DENND1C	DENN domain containing 1C	-1.70	-0.77	2.1E-02	0.040
RPS10P7	ribosomal protein S10 pseudogene 7	-3.57	-1.84	2.3E-02	0.041
KLHL8	kelch like family member 8	1.33	0.42	2.3E-02	0.041
SNORD74	small nucleolar RNA, C/D box 74	-2.61	-1.38	2.3E-02	0.041
CYP2B7P	cytochrome P450 family 2 subfamily B member 7, pseu	2.27	1.18	2.3E-02	0.041
MIR21	microRNA 21	2.29	1.20	2.3E-02	0.041
LOC105747689	uncharacterized LOC105747689	2.00	1.00	2.4E-02	0.041
SAA4	serum amyloid A4, constitutive	4.64	2.21	2.4E-02	0.041
KANSL1-AS1	KANSL1 antisense RNA 1	2.13	1.09	2.5E-02	0.042
COL8A1	collagen type VIII alpha 1 chain	2.27	1.18	2.6E-02	0.042
PRKAG2-AS1	PRKAG2 antisense RNA 1	2.61	1.38	2.7E-02	0.044
LOC101927237	uncharacterized LOC101927237	2.55	1.35	2.7E-02	0.044
EIF4EBP1	eukaryotic translation initiation factor 4E binding protein	1.67	0.74	2.8E-02	0.044
NUDT9	nudix hydrolase 9	1.27	0.34	2.8E-02	0.044
NPPA-AS1	NPPA antisense RNA 1	-2.62	-1.39	2.8E-02	0.044
LOC642361	uncharacterized LOC642361	1.58	0.66	2.8E-02	0.044
HCG22	HLA complex group 22	2.51	1.33	2.8E-02	0.044
SHC3	SHC adaptor protein 3	-1.83	-0.87	3.0E-02	0.045
SPATA9	spermatogenesis associated 9	2.14	1.10	3.0E-02	0.046
TMED6	transmembrane p24 trafficking protein 6	-2.08	-1.06	3.1E-02	0.046
TMEM218	transmembrane protein 218	1.40	0.49	3.1E-02	0.046
FKBP1B	FK506 binding protein 1B	-1.91	-0.93	3.1E-02	0.046
SBF2-AS1	SBF2 antisense RNA 1	1.58	0.66	3.2E-02	0.046
CPT1B	carnitine palmitoyltransferase 1B	2.39	1.26	3.4E-02	0.049
NRIP1	nuclear receptor interacting protein 1	1.41	0.49	3.5E-02	0.049
C14orf39	chromosome 14 open reading frame 39	1.90	0.92	3.6E-02	0.049
ATG16L2	autophagy related 16 like 2	-1.54	-0.63	3.6E-02	0.049
DARS-AS1	DARS antisense RNA 1	2.28	1.19	3.6E-02	0.049
MIR548AR	microRNA 548ar	1.96	0.97	3.7E-02	0.049

PTPN4	protein tyrosine phosphatase, non-receptor type 4	1.27	0.35	3.8E-02	0.049
FBXO16	F-box protein 16	-4.60	-2.20	3.8E-02	0.049
AMOTL2	angiomotin like 2	1.58	0.66	3.9E-02	0.049
PEX10	peroxisomal biogenesis factor 10	1.34	0.42	3.9E-02	0.049
KCNJ2	potassium voltage-gated channel subfamily J member [illegible]	-1.77	-0.82	3.9E-02	0.049
LINC01277	long intergenic non-protein coding RNA 1277	2.96	1.57	3.9E-02	0.049
PAX3	paired box 3	2.45	1.29	4.0E-02	0.049
LONRF1	LON peptidase N-terminal domain and ring finger 1	1.28	0.36	4.0E-02	0.049
DGKI	diacylglycerol kinase iota	1.44	0.53	4.2E-02	0.049
SNORD56	small nucleolar RNA, C/D box 56	-1.90	-0.93	4.2E-02	0.049
PKD1L2	polycystin 1 like 2 (gene/pseudogene)	2.02	1.01	4.3E-02	0.049
EFNA5	ephrin A5	1.45	0.54	4.3E-02	0.049
LOC100126784	uncharacterized LOC100126784	-2.95	-1.56	4.3E-02	0.049
VSTM4	V-set and transmembrane domain containing 4	1.35	0.43	4.3E-02	0.049
SIM1	single-minded family bHLH transcription factor 1	2.94	1.55	4.3E-02	0.049
ANO3	anoctamin 3	1.79	0.84	4.3E-02	0.049
AXIN2	axin 2	1.54	0.62	4.4E-02	0.049
LOC100287015	uncharacterized LOC100287015	-1.77	-0.83	4.4E-02	0.049
LOC728730	uncharacterized LOC728730	1.57	0.65	4.4E-02	0.049
DNAL4	dynein axonemal light chain 4	1.36	0.44	4.4E-02	0.049
FBXL17	F-box and leucine rich repeat protein 17	1.24	0.31	4.4E-02	0.049
PGBD4	piggyBac transposable element derived 4	1.64	0.72	4.5E-02	0.049
DTX3	deltex E3 ubiquitin ligase 3	-1.62	-0.70	4.5E-02	0.049
MIR7-1	microRNA 7-1	-5.14	-2.36	4.5E-02	0.049
KLHDC10	kelch domain containing 10	1.24	0.31	4.7E-02	0.049
LOC101927932	uncharacterized LOC101927932	1.94	0.96	4.7E-02	0.049
GZMM	granzyme M	-3.68	-1.88	4.7E-02	0.049
FMO2	flavin containing monooxygenase 2	1.69	0.75	4.7E-02	0.049
IRAK3	interleukin 1 receptor associated kinase 3	1.27	0.35	4.7E-02	0.049
SYTL1	synaptotagmin like 1	-2.11	-1.08	4.8E-02	0.049
PCDHB7	protocadherin beta 7	1.36	0.45	4.8E-02	0.049
MS4A1	membrane spanning 4-domains A1	2.22	1.15	4.8E-02	0.049
TIPIN	TIMELESS interacting protein	1.51	0.60	4.8E-02	0.049
LOC100506551	uncharacterized LOC100506551	2.54	1.34	4.8E-02	0.049
LOC101927865	uncharacterized LOC101927865	1.71	0.77	4.8E-02	0.049
CENPH	centromere protein H	1.57	0.65	4.8E-02	0.049
RGS22	regulator of G-protein signaling 22	1.55	0.63	4.8E-02	0.049
RRS1-AS1	RRS1 antisense RNA 1 (head to head)	1.63	0.70	4.9E-02	0.049
RAI2	retinoic acid induced 2	1.37	0.45	5.0E-02	0.050
LIN7A	lin-7 homolog A, crumbs cell polarity complex compone[illegible]	1.59	0.67	5.0E-02	0.050

*FC: Fold Change

Supplementary Table 7: Canonical pathway analysis of the microdissected breast epithelium RNAseq dataset

	Ingenuity Canonical Pathways	-log(p-value)	Ratio	Molecules
Upregulated Genes	PPAR signaling pathway	4.95	0.06	PPARG,APOB,LPL,MAPK8,MAPK10,NR5A2,RBP4
	Adipogenesis pathway	3.79	0.04	PPARG,FZD4,LEP,PLIN1,LPL,FABP4
	AMPK Signaling	3.47	0.03	LEP,ACACB,ADRA2A,CHRNB2,ADIPOQ,LIPE,KLB
	PPARα/RXRα Activation	3.11	0.03	GPD1,CD36,LPL,ADIPOQ,MAPK8,ACVR1C
	TR/RXR Activation	2.53	0.04	KLF9,AKR1C1/AKR1C2,PDE3B,KLB
	Paxillin Signaling	2.31	0.04	MAPK8,MAPK10,KLB,ACTA1
	LXR/RXR Activation	2.21	0.03	APOB,CD36,LPL,RBP4
	Insulin signaling pathway	1.98	0.03	PDE3B,MAPK8,LIPE,KLB
	cAMP-mediated signaling	1.91	0.02	PDE2A,ADRA2A,PDE3B,RAPGEF3,PKIA
	Retinol Biosynthesis	1.58	0.05	LPL,LIPE
Downregulated Genes	Metalloproteases Inhibition	2.43	0.05	MMP27,SDC2
	PCP pathway	2.05	0.03	ROR2,SMO
	Th1/Th2 Activation	2.04	0.02	CXCR6,IL27RA,CD8A

Supplementary Table 8: Ingenuity network analysis of the microdissected breast epithelium-related RNAseq dataset: upregulated (Up) and downregulated (Down) genes are analyzed together or separately

ID	Analysis	Molecules in Network	Score	Focus	Top Diseases and Functions
1	Epithelium Up and Down	ACACB,Adipokine,AMH,C/ebp,CIDEC,Cpla2,CPT1,CXCR5,ERK1/2,F8,FABP,G0S2,GPRIN2,GUC	31	18	Connective Tissue Development and Function, Lipid Metabolism, Small Molecule Biochemistry
2	Epithelium Up and Down	ACAC,ADIPOQ,AIM2,AKR1C1/AKR1C2,CD36,Collagen Alpha1,Collagen type I,EFNB3,ELOVL5,F	29	17	Lipid Metabolism, Molecular Transport, Small Molecule Biochemistry
3	Epithelium Up and Down	26s Proteasome,ACTA1,AMPK,APOB,Cg,DHRS12,EMX2,FSH,Gsk3,Hdac,Histone h3,Histone h4	29	17	Endocrine System Disorders, Metabolic Disease, Cardiovascular Disease
4	Epithelium Up and Down	ABCB5,ABCC6,AKR1C1/AKR1C2,ALDH1L1,CCS,CDH1,CHRNB2,CHST6,CTSF,CTSG,FAM129	29	17	Cancer, Organismal Injury and Abnormalities, Reproductive System Disease
5	Epithelium Up and Down	AR,ARMCX2,Ca2+,CGNL1,creatine,DNASE1,elaidic acid,FGF23,GHSR,GSK3B,H19,HEPACAM,	22	14	Connective Tissue Disorders, Organismal Injury and Abnormalities, Skeletal and Muscular Disorders
6	Epithelium Up and Down	ACVR1C,Akt,Alp,BCR (complex),CIDEA,CPA4,DLEU1,E2f,EMP1,Frizzled,HDC,IFN Beta,Ige,IgG,	20	13	Cellular Movement, Reproductive System Development and Function, Organ Morphology
7	Epithelium Up and Down	ADCY,ADRB,AQP7,Beta Arrestin,Calcineurin protein(s),CaMKII,Cofilin,COL8A2,collagen,Creb,cyt	20	13	Cell Morphology, Organ Morphology, Skeletal and Muscular System Development and Function
8	Epithelium Up and Down	ADRA2A,Ap1,Collagen(s),CXCR6,estrogen receptor,Focal adhesion kinase,FZD4,G protein alphai,	20	13	Cardiovascular Disease, Gastrointestinal Disease, Hepatic System Disease
9	Epithelium Up and Down	ANKRD29,APOBEC3G,APP,BTNL9,C19orf18,CAVIN4,Chil3/Chil4,CLDN16,CLDN19,CLIC5,FAN	20	13	Neurological Disease, Organismal Injury and Abnormalities, Hematological Disease
10	Epithelium Up and Down	Akr1b7,APOA1,Apoc3,C1orf21,CDCA7,CDH3,COL11A1,COL12A1,CYP39A1,CYP4A22,dihydrote	20	13	Connective Tissue Development and Function, Tissue Morphology, Cancer
11	Epithelium Up and Down	Actin,AIM2,ANG,ARPP21,ATF6,caspase,CD3,CD8A,CLCN1,cytokine,EMX2OS,FAS,FYB2,GATA	18	12	Cell Death and Survival, Cancer, Hereditary Disorder
12	Epithelium Up and Down	ANAPC13,AREG,ARRB2,CCDC32,CDCA2,CDK4,CMKLR1,CPEB2,DRD3,Endothelin,FFAR4,FO	16	11	Endocrine System Development and Function, Molecular Transport, Small Molecule Biochemistry
13	Epithelium Up and Down	ACAT1,BICD2,C19orf73,Calmodulin,CDC123,CEP19,CEP85,COMMD2,CPNE5,EPB41L2,EXOC	16	11	Cellular Development, Cellular Growth and Proliferation, Embryonic Development
14	Epithelium Up and Down	ARMCX4,DPAGT1,PDE4DIP	2	1	Developmental Disorder, Hereditary Disorder, Immunological Disease

ID	Analysis	Molecules in Network	Score	Focus	Top Diseases and Functions
1	Epithelium Up	ABCC6,ACAC,ACACB,Adipokine,AMH,CD36,CIDEC,Cpla2,CPT1,CXCR5,ERK1/2,FABP,FABP4,	41	21	Lipid Metabolism, Small Molecule Biochemistry, Connective Tissue Development and Function
2	Epithelium Up	ABCB5,Acaa1b,Akr1b7,AKR1C4,AKR1C1/AKR1C2,ALDH1L1,AREG,C1orf21,Ca2+,CDH1,CPEB	29	16	Lipid Metabolism, Small Molecule Biochemistry, Developmental Disorder
3	Epithelium Up	ACVR1C,ADCY,ADRB,AIM2,AKR1C1/AKR1C2,C/ebp,Calcineurin protein(s),CaMKII,collagen,Coll	24	14	Cell Morphology, Organ Morphology, Skeletal and Muscular System Development and Function
4	Epithelium Up	acetic acid,ANKRD29,ARRB2,BTNL9,C9orf116,CAMK2N2,CLEC10A,cytokine,FAM221B,FAM81E	24	14	Cellular Compromise, Cellular Development, Cellular Growth and Proliferation
5	Epithelium Up	beta-estradiol,C19orf73,FAM214A,GABRP,GATA2,GPX3,Gypa,HEPACAM,HGFAC,Iigp1,Kap,KR	22	13	Infectious Diseases, Nervous System Development and Function, Cancer
6	Epithelium Up	APP,ATF6,C19orf18,CLDN19,CLIC5,creatine,FAM129C,FCHO1,GSK3B,GYG2,HINT2,HOXA9,H	22	13	Hematological System Development and Function, Hematopoiesis, Lymphoid Tissue Structure and Development
7	Epithelium Up	ADIPOQ,Akt,Alp,AMPK,AQP7,CIDEA,Cofilin,Collagen type I,ELOVL5,EMP1,GPD1,IFN Beta,Ige,I	20	12	Cell Morphology, Connective Tissue Development and Function, Tissue Morphology
8	Epithelium Up	26s Proteasome,ACTA1,Actin,caspase,Cg,cytochrome C,DHRS12,F Actin,FSH,FZD4,GNRH,Gsk	20	12	Skeletal and Muscular System Development and Function, Cellular Assembly and Organization, Cellular Development
9	Epithelium Up	3,5-diiodothyronine,A1CF,AADAC,Alpha 1 antitrypsin,ANAPC13,ANGPTL3,APOA1,APOA5,APOB	12	8	Lipid Metabolism, Molecular Transport, Small Molecule Biochemistry
10	Epithelium Up	ADRA2A,Ap1,BCR (complex),Beta Arrestin,CD3,CHRNB2,Collagen(s),DLEU1,E2f,ERK,estrogen r	10	7	Neurological Disease, Skeletal and Muscular Disorders, Cancer
11	Epithelium Up	ARMCX4,DPAGT1,PDE4DIP	2	1	Developmental Disorder, Hereditary Disorder, Immunological Disease

ID	Analysis	Molecules in Network	Score	Focus	Top Diseases and Functions
1	Epithelium Down	5'-methylthioadenosine,CD3,CD8A,CPA4,CXCR6,EFNB3,EMX2,ERK,GPR18,GUCY1A1,GUCY1E	42	18	Cardiovascular System Development and Function, Embryonic Development, Organ Development
2	Epithelium Down	APP,AR,ARMCX2,BRD3,Ca2+,CALB2,CAVIN4,CEP19,COL4A6,COL8A2,DNMT1,EGFL6,ESR1,	30	14	Gene Expression, DNA Replication, Recombination, and Repair, Organ Morphology
3	Epithelium Down	5'-methylthioadenosine,CCDC32,CCND1,CHST6,CYP39A1,DLK1,DNMT1,ESR2,FGF2,GATA4,gl	25	12	Gene Expression, Cellular Development, Cellular Growth and Proliferation
4	Epithelium Down	AAAS,ARL1,ARL2,BICD2,CCDC47,CDCA2,CGNL1,CHMP1A,CHMP2A,CPNE5,DNMT1,EED,FA	11	6	Cell Cycle, DNA Replication, Recombination, and Repair, Molecular Transport
5	Epithelium Down	EMX2OS,NFYB	2	1	Cellular Development, Gene Expression, Cancer
6	Epithelium Down	FAS,HLA-B,HLA-B27,LILRA1,LILRA3	2	1	Dermatological Diseases and Conditions, Organismal Injury and Abnormalities, Gastrointestinal Disease

Supplementary Table 9: List of canonical pathways linked with the genes differentially expressed between susceptible and matched healthy control breast stroma samples

	Ingenuity Canonical Pathways	-log(p-value	Ratio	Molecules
UPREGULATED	PPARα/RXRα Activation	7.61	0.09	GPD1,PRKAB1,ACAA1,CD36,ADCY3,ADCY6,ADIPOQ,IKBKG,GHR,PRKAR2B,NFKBIA,FASN,LPL,MRAS,ADIPOR2,ACVR1C
	RAR Activation	6.49	0.08	DHRS3,AKR1C3,ADCY3,ADCY6,TNIP1,PRKAR2B,RBP7,RDH16,IGFBP3,MAPK10,NR2F6,RBP5,RXRB,ZBTB16,RBP4
	Retinol Biosynthesis	5.45	0.17	DHRS3,RBP7,AKR1C3,LPL,LIPE,RBP5,PNPLA2
	FXR/RXR Activation	5.34	0.09	APOE,SLC27A5,MLXIPL,APOB,SCARB1,FASN,SAA1,LPL,MAPK10,SAA2,RBP4
	LXR/RXR Activation	4.69	0.08	APOE,MLXIPL,APOB,FASN,CD36,SAA1,LPL,SAA2,RXRB,RBP4
	Relaxin Signaling	3.71	0.06	PDE2A,IKBKG,NFKBIA,PRKAR2B,NPR1,ADCY3,MRAS,ADCY6,GNAI1,PDE8B
	Protein Kinase A Signaling	3.22	0.04	PDE2A,MYL2,AKAP8,BAD,ADCY3,GNAI1,ADCY6,LIPE,PTPRM,IKBKG,PRKAR2B,NFKBIA,DUSP4,PDE8B,AKAP1,PHKG1
	Triacylglycerol Degradation	2.86	0.09	LPL,LIPE,MGLL,PNPLA2,PRDX6
DOWNREGULATED	Anti-viral Immunity	4.16	0.38	APOBEC3B,APOBEC3G,TK1
	TREM1 Signaling	2.86	0.07	CXCL3,NOD2,TLR1,TLR7,LAT2
	Eicosanoid Signaling	2.2	0.06	PTGFR,ALOX12,ALOX5,PTGER4
	Granzyme B Signaling	1.89	0.13	APAF1,LMNB1
	Th1 Pathway	1.78	0.04	CCR5,PIK3CG,IL6R,CD8A,KLRC1
	G-Protein Coupled Receptor Signaling	1.48	0.03	GRM7,P2RY13,PIK3CG,GNAO1,RGS14,PTGER4,XCR1
	Mitotic Roles of Polo-Like Kinase	1.45	0.05	PTTG1,CDC23,CCNB1
	cAMP-mediated signaling	1.4	0.03	GRM7,P2RY13,GNAO1,RGS14,PTGER4,XCR1
	Th1 and Th2 Activation Pathway	1.28	0.03	CCR5,PIK3CG,IL6R,CD8A,KLRC1

Supplementary Table 10: Ingenuity network analysis of the microdissected breast stroma-related RNAseq dataset: upregulated (Up) and downregulated (Down) genes are analyzed together or separately

ID	Analysis	Molecules in Network	Score	Focus M	Top Diseases and Functions
1	Stroma Up and Down	ASPH,BAALC,Cbp/p300,CCDC12,CCDC120,CDC37,CDK9,CEP97,COQ8A,DMRT3,EAF2,FR	46	30	Gene Expression, Developmental Disorder, Hereditary Disorder
2	Stroma Up and Down	ACVR1C,Alpha tubulin,AMOTL2,APOBEC3B,APOBEC3F,APOBEC3G,B-cell receptor,CCNJL	41	28	Antimicrobial Response, RNA Post-Transcriptional Modification, Cell Signaling
3	Stroma Up and Down	26s Proteasome,AAMP,AIFM2,CA4,CEBPA,CYP39A1,EPHX1,estrogen receptor,FAM212B,FA	37	26	Cellular Development, Cellular Growth and Proliferation, Hematological System Development and Function
4	Stroma Up and Down	AFG3L2,AKAP,AKAP1,Akt,ANGPTL4,CDK4/6,CYP19,ELOVL5,FADS3,FNDC3A,G0S2,GRAM	35	25	Lipid Metabolism, Small Molecule Biochemistry, Infectious Diseases
5	Stroma Up and Down	ACAC,AHR,APAF1,APIP,BOP1,Cdk,CDKN2B,CFAP206,CPT1,CPT1C,Cyclin A,Cyclin D,Cycli	35	25	Cell Cycle, DNA Replication, Recombination, and Repair, Endocrine System Disorders
6	Stroma Up and Down	ABLIM3,Actin,ADCY3,Alpha Actinin,Alpha catenin,Cadherin,Calmodulin,CAV1,CAVIN1,CDH5,	33	24	Connective Tissue Disorders, Developmental Disorder, Hereditary Disorder
7	Stroma Up and Down	ACADS,APC (complex),chemokine receptor,chymotrypsin,COL13A1,COL16A1,COL25A1,COL	31	23	Cancer, Connective Tissue Disorders, Organismal Injury and Abnormalities
8	Stroma Up and Down	ADIPOQ,ADIPOR2,AGPAT2,AMPK,CDO1,Cofilin,CYB5A,cytochrome C,cytochrome-c oxidase	31	23	Lipid Metabolism, Small Molecule Biochemistry, Connective Tissue Disorders
9	Stroma Up and Down	ABCD2,ACAA1,AKR1C3,AKR1C1/AKR1C2,ANO3,DHRS3,ECM2,FABP,GAS2,JUN/JUNB/JUN	29	22	Endocrine System Development and Function, Lipid Metabolism, Small Molecule Biochemistry
10	Stroma Up and Down	20s proteasome,ACO1,ANGPT4,ANKRD2,ATP1A2,Atrial Natriuretic Peptide,Collagen Alpha1	27	21	Developmental Disorder, Small Molecule Biochemistry, Hereditary Disorder
11	Stroma Up and Down	AMIGO1,Ap1,APOL6,BLCAP,caspase,CCNB1,CCNB1IP1,CD3,CDC23,Cg,Creb,CS,Cyclin B,F	27	21	Cell Cycle, Hair and Skin Development and Function, Cardiovascular Disease
12	Stroma Up and Down	AKAP8,AOC3,AQP7,Cdc2,CH25H,CYTIP,DUSP4,Erm,ETFDH,Gamma tubulin,GLDC,HOXC4	25	20	Humoral Immune Response, Protein Synthesis, Developmental Disorder
13	Stroma Up and Down	ACACB,ACSL6,ALOX5,ALOX12,APOB,BCR (complex),BLNK,CD36,CD3 group,Collagen type	23	19	Lipid Metabolism, Small Molecule Biochemistry, Energy Production
14	Stroma Up and Down	Adaptor protein 2,ADGRF5,CCR2,CCR5,chemokine,FZD4,G protein alphai,G protein beta gar	23	19	Behavior, Organismal Injury and Abnormalities, Tissue Morphology
15	Stroma Up and Down	ALT,APOE,C19orf12,Complement,CXCL3,cytokine,DNAJA4,FAH,GOT,HARS,hemoglobin,HIS	23	19	Organismal Injury and Abnormalities, Digestive System Development and Function, Hepatic System Development and Function
16	Stroma Up and Down	14-3-3,ADRB,ARL4A,BAD,BOK,Calcineurin protein(s),CaMKII,Caspase 3/7,CUTC,CXorf21,DE	21	18	Hematological System Development and Function, Hematopoiesis, Humoral Immune Response
17	Stroma Up and Down	CD38,CD8A,Chuk-Ikbkb-Ikbkg,GAS5,GCAT,GFOD1,Ifn,IFN alpha/beta,IFN Beta,IFN type 1,Ifn	21	18	Cell-To-Cell Signaling and Interaction, Hematological System Development and Function, Immune Cell Trafficking
18	Stroma Up and Down	ACVR1C,APMAP,ATAD2,ATPase,ATXN1L,COPRS,DQX1,FAM210A,FBXO24,FNBP1,HIST1H	21	18	Cardiac Dilation, Cardiovascular Disease, Hereditary Disorder
19	Stroma Up and Down	Alp,calpain,CAPN2,Collagen(s),EDEM1,ESYT1,FERMT1,FGF2,HEY1,Hif1,Integrin,Laminin (c	20	17	Organismal Development, Cellular Development, Cellular Growth and Proliferation
20	Stroma Up and Down	ALDH4A1,ANO2,APP,BCAT2,beta-estradiol,C1orf87,CDCA7,CDR1,COL13A1,DNAL1,DNALI1	20	17	Cellular Assembly and Organization, Cell-To-Cell Signaling and Interaction, Inflammatory Response
21	Stroma Up and Down	ABCD2,ACOT4,ADAT1,BCL2L1,CASS4,CEP89,CETN3,CRK,CYP3A7,FRMD4A,GYS2,HBP1,	20	17	Cell Morphology, Cellular Function and Maintenance, Connective Tissue Development and Function
22	Stroma Up and Down	ADGRE1,AGAP5 (includes others),BIN2,Calm1 (includes others),CCDC32,CD44,DAPK2,DAF	18	16	Cell Signaling, Nucleic Acid Metabolism, Small Molecule Biochemistry
23	Stroma Up and Down	ADCY,ADCY6,Ampa Receptor,C-C chemokine receptor,CASP4,CTNNBIP1,G protein,G protei	16	15	Cell Signaling, Molecular Transport, Nucleic Acid Metabolism
24	Stroma Up and Down	ALDH4A1,ALOX12,APP,ARHGEF5,BCAT2,C1orf35,C7orf31,CCDC144NL,CCL13,CDH3,CEL	16	15	Hematological Disease, Metabolic Disease, Cellular Compromise
25	Stroma Up and Down	ACACB,ADAM11,ADAM22,ADAM23,C2CD2,CD8B,CFAP45,CREB3L2,DCUN1D3,ELAVL1,FA	16	15	Nervous System Development and Function, Developmental Disorder, Hereditary Disorder

ID	Analysis	Molecules in Network	Score	Focus M	Top Diseases and Functions
1	Stroma Up	ACVR1C,AMOTL2,ASPH,Cadherin,CCDC12,CDH5,COL13A1,COQ8A,CTNNBIP1,DTX1,ERK	51	29	Developmental Disorder, Embryonic Development, Organismal Development
2	Stroma Up	ADIPOQ,ADIPOR2,AGPAT2,APOE,AQP7,Atrial Natriuretic Peptide,ATXN1L,chymotrypsin,DD	45	27	Carbohydrate Metabolism, Connective Tissue Disorders, Organismal Injury and Abnormalities
3	Stroma Up	Actin,Alpha catenin,APOL6,BAALC,C19orf12,caspase,CD3,CDC37,Ck2,cytokine,DMRT3,ESY	34	22	Cell Death and Survival, Dermatological Diseases and Conditions, Developmental Disorder
4	Stroma Up	20s proteasome,ACADS,ACO1,ANGPT4,ANKRD2,APOB,ATP1A2,creatine kinase,ERK1/2,Fe	32	21	Molecular Transport, Small Molecule Biochemistry, Developmental Disorder
5	Stroma Up	AKAP,AKAP1,Akt,ALT,ANGPTL4,CD36,CDK4/6,CYP19,DDIT4,ELOVL5,FASN,FXR ligand-FX	32	21	
6	Stroma Up	ABCD2,ACAA1,ACAC,ACACB,AKR1C3,AKR1C1/AKR1C2,ANO3,CPT1,DHRS3,FABP,G0S2,I	30	20	Energy Production, Lipid Metabolism, Small Molecule Biochemistry
7	Stroma Up	26s Proteasome,AFG3L2,Alpha tubulin,BOP1,BRPF1,CA4,CDK9,CEBPA,CSDC2,Cyclin A,HA	27	19	Cell Cycle, Connective Tissue Development and Function, Cell Morphology
8	Stroma Up	7beta-hydroxycholesterol,ABLIM3,APP,BCL2,C19orf12,Ca2+,D-serine,DNAJB12,DRAP1,ECM	27	19	Skeletal and Muscular System Development and Function, Cell Morphology, Renal and Urological System Development and Function
9	Stroma Up	AMIGO1,AOC3,COL16A1,COL25A1,COL4A2,collagen,Collagen type I,Collagen type IV,Collac	24	17	Organismal Development, Cancer, Organismal Injury and Abnormalities
10	Stroma Up	ADGRE1,ADGRV1,AIFM2,APOBEC3B,ATP1A2,CD300LG,CEP55,CWC25,FANCD2,FTSJ3,G	22	16	Protein Trafficking, Hereditary Disorder, Ophthalmic Disease
11	Stroma Up	AAMP,ADCY,Ampa Receptor,CaMKII,Cdk,Chuk-Ikbkb-Ikbkg,Collagen Alpha1,Cyclin D,E2f,ETI	20	15	Developmental Disorder, Endocrine System Disorders, Hereditary Disorder
12	Stroma Up	ADAM11,ADAM23,APIP,APMAP,ASAP2,BLCAP,C2CD2,C9orf64,CFAP45,DCUN1D3,DUSP28	20	15	Connective Tissue Disorders, Developmental Disorder, Hereditary Disorder
13	Stroma Up	ADGRF5,AGTR1,ANO2,BCAT2,beta-estradiol,CDCA7,CDR1,CLDND2,CMKLR1,COPRS,CRE	20	15	Post-Translational Modification, Cell-To-Cell Signaling and Interaction, Nervous System Development and Function
14	Stroma Up	ADGRF5,ALDH1L1,CD44,CTRB2,CUTC,D-erythro-C16-ceramide,DICER1,ENDOU,F11,FRMI	20	15	Cardiovascular System Development and Function, Cell-To-Cell Signaling and Interaction, Hematological System Development and Function
15	Stroma Up	ADCY3,ADGRF5,AKAP8,AMPK,Ap1,Calmodulin,CAPN2,CAV1,CS,DAPK2,estrogen receptor,	18	14	Cell Signaling, Nucleic Acid Metabolism, Small Molecule Biochemistry
16	Stroma Up	ALDH1B1,ALDH4A1,C9orf116,CAMK2N2,CTIF,DHRS4,DHRS4L2,DNASE1,EPHX1,FKBP3,ga	18	14	Cell Morphology, Hematological Disease, Metabolic Disease
17	Stroma Up	14-3-3,ADCY6,ADRB,BAD,BOK,Calcineurin protein(s),CD3 group,CDO1,Cg,Creb,DBNL,DUS	16	13	Cell Death and Survival, Embryonic Development, Cellular Growth and Proliferation
18	Stroma Up	AJAP1,ANXA9,ARL4D,ATP5PO,C20orf27,CDKN2B,CHCHD10,COL12A1,CTNNB1,Cyclin D1/	16	13	Connective Tissue Development and Function, Skeletal and Muscular System Development and Function, Tissue Development
19	Stroma Up	ADPGK,AGRN,APP,ATM,C1orf35,CARS,CCDC144NL,CCNB1IP1,CCNG2,CRHR2,DAPP1,DF	15	12	Nervous System Development and Function, Organ Morphology, Tissue Morphology
20	Stroma Up	Alp,BCR (complex),CAVIN1,Complement,CYB5A,cytochrome C,cytochrome-c oxidase,EGLN,	13	11	Developmental Disorder, Gastrointestinal Disease, Hematological Disease
21	Stroma Up	FAM89A,UBXN2B	2	1	Cell Cycle, Cellular Assembly and Organization, Cancer
22	Stroma Up	BTNL9,STAT1	2	1	Cellular Development, Cellular Growth and Proliferation, Hematological System Development and Function
23	Stroma Up	ABL1,TMEM74B	2	1	Cancer, Cardiovascular Disease, Cellular Assembly and Organization
24	Stroma Up	MIR5091,miR-5091 (miRNAs w/seed CGGAGAC)	2	1	
25	Stroma Up	MIR6079,miR-6079 (miRNAs w/seed UGGAAGC)	2	1	

ID	Analysis	Molecules in Network	Score	Focus M	Top Diseases and Functions
1	Stroma Down	AHR,Akt,APAF1,APC (complex),CCNB1,CCNB1IP1,CD38,Cdc2,CDC23,Cyclin A,Cyclin D,Cyc	37	22	Cell Cycle, Cell Death and Survival, Hepatic System Development and Function
2	Stroma Down	B-cell receptor,BCR (complex),BLNK,CH25H,DAPP1,Eotaxin,ERK1/2,FCRL5,HOXC4,HSH2D	33	20	Humoral Immune Response, Protein Synthesis, Cell-To-Cell Signaling and Interaction
3	Stroma Down	ALOX5,CCR2,CCR5,chemokine,chemokine receptor,Cofilin,Collagen type II,Collagen(s),CXCI	30	19	Cellular Movement, Hematological System Development and Function, Immune Cell Trafficking
4	Stroma Down	ADGRF5,APP,C7orf31,CPO,CYP20A1,DNAL1,DOC2A,DOC2B,DYNLT1,FASTKD3,Gpcr,GPF	26	17	Cell-To-Cell Signaling and Interaction, Cell Death and Survival, Cellular Compromise
5	Stroma Down	AGAP5 (includes others),ALOX12,ARMT1,ATAD2,beta-estradiol,C16orf74,CCDC32,CPT1C,D	26	17	Lipid Metabolism, Small Molecule Biochemistry, Organ Morphology
6	Stroma Down	A2M,ACSL6,ARL4A,CASS4,CCL24,CCNJL,CEP89,CNR1,CRK,ECM2,GCAT,GFM2,GPR84,G	26	17	Cell-To-Cell Signaling and Interaction, Dermatological Diseases and Conditions, Organismal Injury and Abnormalities
7	Stroma Down	APOBEC3B,APOBEC3F,APOBEC3G,C-C chemokine receptor,CASP4,CD8A,cytidine deamin	24	16	Antimicrobial Response, Inflammatory Response, Cell Signaling
8	Stroma Down	14-3-3,Actin,ALOX12,Alpha tubulin,CYTIP,ERK,F Actin,Fcer1,FERMT1,GAS2,GAS2L3,HDL,H	24	16	Cancer, Endocrine System Disorders, Gastrointestinal Disease
9	Stroma Down	ACOX3,ADAT1,CHKB-AS1,Coup-Tf,DAPK2,E2F4,E2F7,FBXW2,FPGT,GINS2,HIF1A,HIST2H	24	16	Cellular Function and Maintenance, Cell Cycle, Cancer
10	Stroma Down	AFMID,ALOX12,BCL2L1,CCSAP,CCT4,CTNS,CYP39A1,EID2B,EMX2OS,ESR1,GRB2,HBP1,	24	16	Cellular Growth and Proliferation, Cell Cycle, Tissue Morphology
11	Stroma Down	ADCY,ADRB,AMPK,Calmodulin,Cg,CPNE5,Creb,FNDC3A,G protein alphai,G protein beta gar	22	15	Cancer, Dermatological Diseases and Conditions, Organismal Injury and Abnormalities
12	Stroma Down	26s Proteasome,ALOX12,Ap1,caspase,CD3,CYP4V2,FSH,GLDC,HDL-cholesterol,HIST1H4D	22	15	Nucleic Acid Metabolism, Small Molecule Biochemistry, Cellular Development
13	Stroma Down	ADAT1,ANKRD49,APH1A,CAMK2N1,DPY19L4,DTX3L,ELAVL1,EVI2A,FAM210A,FAM46C,G/	22	15	Developmental Disorder, Hereditary Disorder, Neurological Disease
14	Stroma Down	12-hydroxyeicosatetraenoic acid,ALOX12,APOB,BIN2,CEP97,DERL3,ECE1,EGFR,ERN1,F2,F	18	13	Lipid Metabolism, Small Molecule Biochemistry, Vitamin and Mineral Metabolism
15	Stroma Down	ADCYAP1,CCDC120,CCDC168,Ccl6,CEACAM,CEACAM21,CENPQ,CFAP206,CREBBP,CUL	16	12	Cellular Development, Behavior, Cancer
16	Stroma Down	MEI1,NEDD8	2	1	Post-Translational Modification, Cell Cycle, Reproductive System Development and Function
17	Stroma Down	KANSL1L,OPTN	2	1	Cell Death and Survival, Cell Morphology, Cellular Function and Maintenance
18	Stroma Down	miR-92a-3p (and other miRNAs w/seed AUUGCAC),OIP5-AS1	2	1	Cancer, Organismal Injury and Abnormalities, Gastrointestinal Disease
19	Stroma Down	mir-1254,miR-1254 (and other miRNAs w/seed GCCUGGA)	2	1	
20	Stroma Down	mir-7641,miR-7641 (miRNAs w/seed UGAUCUC)	2	1	
21	Stroma Down	NALP,NLRP11	2	1	Cancer, Gastrointestinal Disease, Hepatic System Disease
22	Stroma Down	MIR5586,miR-5586-3p (miRNAs w/seed AGAGUGA),miR-5586-5p (miRNAs w/seed AUCCAC	2	1	
23	Stroma Down	mir-3064,miR-3064-3p (miRNAs w/seed GCCACAC),miR-3064-3p (miRNAs w/seed UGCCAC	1	1	

Supplementary Table 11: List of canonical pathway involving the genes differentially expressed in breast adipose tissue between susceptible and matched healthy control samples.

	Ingenuity Canonical Pathways	-log(p-value)	Ratio	Molecules
UPREGULATED	Mitochondrial L-carnitine Shuttle Pathway	2.79	0.118	SLC27A2,CPT1B
	PPARα/RXRα Activation	2.41	0.0222	NFKBIA,CPT1B,ADCY10,ACVR1C
	Immune response regulation	2.36	0.0326	NFKBIA,IRAK3,ADCY10
	Fatty Acid β-oxidation I	2.24	0.0625	SLC27A2,SCP2
	Glutamate Receptor Signaling	1.76	0.0351	GRIA1,GLUL
	AMPK Signaling	1.38	0.0139	CPT1B,ADRB3,EIF4EBP1
	GABA Receptor Signaling	1.35	0.0211	ADCY10,SLC6A13
	Sumoylation Pathway	1.34	0.0208	NFKBIA,SENP3
	TR/RXR Activation	1.32	0.0204	KLF9,THRB
	ATM Signaling	1.32	0.0204	NFKBIA,GADD45B
DOWNREGULATED	Retinol Biosynthesis	2.67	0.0476	CES4A,LIPG
	Salvage Pathways of Pyrimidine Deoxyribor	1.88	0.125	APOBEC3G
	B Cell Development	1.72	0.0154	BLNK,CAMK2B
	B Cell Receptor Signaling	1.4	0.0104	BLNK,CAMK2B

Supplementary Table 12: Ingenuity network analysis of the microdissected breast adipose tissue-related RNAseq dataset: Upregulated (Up) and downregulated (Down) genes are analyzed together or separately.

ID	Analysis	Molecules in Network	Score	Focus M	Top Diseases and Functions
1	Adipose Tissue Up and Down	ACVR1C,ADCY,ADCY10,ADRB3,Akt,AMOTL2,AMPK,AXIN2,c	32	17	Cardiovascular Disease, Cardiac Arrythmia, Organismal Injury and Abnormalities
2	Adipose Tissue Up and Down	AMIGO1,ASGR1,BCR (complex),BLNK,Calcineurin protein(s),(	30	16	Cell Death and Survival, Tissue Morphology, Cellular Compromise
3	Adipose Tissue Up and Down	ADRB,ANO3,Ap1,APOBEC3G,CaMKII,CERS6,Cg,Ck2,COL8A	30	16	Cellular Function and Maintenance, Molecular Transport, Lipid Metabolism
4	Adipose Tissue Up and Down	ACOX3,ARMC1,ASCC1,BPIFB2,BRIP1,BST1,C21orf59,CCDC	25	14	Cell Morphology, Cellular Function and Maintenance, Cell Death and Survival
5	Adipose Tissue Up and Down	ACOT7,APP,ARMC1,ASCC1,ATXN1,BRCA1,CAMK2B,CHURC	23	13	Cell-To-Cell Signaling and Interaction, Nervous System Development and Function, Cellular Assembly and Organization
6	Adipose Tissue Up and Down	AR,ATAD2,beta-estradiol,CES4A,CLCN1,DNAH6,DNAH8,DNA	20	12	Organ Morphology, Reproductive System Development and Function, Cellular Growth and Proliferation
7	Adipose Tissue Up and Down	10-nitrooleate,22(R)-hydroxycholesterol,AP4M1,ATG16L2,ATP	18	11	Small Molecule Biochemistry, Connective Tissue Development and Function, Tissue Morphology
8	Adipose Tissue Up and Down	26s Proteasome,ACSF3,ADRB3,alpha-hydroxyglutarate,ANK1,	12	8	Organ Morphology, Renal Dilation, Renal and Urological System Development and Function
9	Adipose Tissue Up and Down	mir-921,miR-921 (miRNAs w/seed UAGUGAG)	2	1	
10	Adipose Tissue Up and Down	MIR5690,miR-5690 (miRNAs w/seed CAGCUAC)	2	1	
11	Adipose Tissue Up and Down	CYP26C,CYP26C1	2	1	Dermatological Diseases and Conditions, Developmental Disorder, Drug Metabolism
12	Adipose Tissue Up and Down	CA13,Carbonic anhydrase	2	1	Ophthalmic Disease, Organismal Injury and Abnormalities, Developmental Disorder
13	Adipose Tissue Up and Down	PARVG,TAL1,WFDC1,ZNF677	2	1	Cardiovascular System Development and Function, Cell Cycle, Cellular Development

ID	Analysis	Molecules in Network	Score	Focus M	Top Diseases and Functions
1	Adipose Tissue Up	ADCY10,APP,ATXN1,beta-estradiol,BPIFB2,CFL2,CHURC1,C	41	19	Nucleic Acid Metabolism, RNA Post-Transcriptional Modification, Small Molecule Biochemistry
2	Adipose Tissue Up	ADCY,ADCY10,ADRB,Calcineurin protein(s),CaMKII,Cg,CPT1	38	18	Amino Acid Metabolism, Cancer, Endocrine System Disorders
3	Adipose Tissue Up	AMIGO1,AR,BRIP1,CCND1,CENPH,COL11A1,COL8A1,COL8	30	15	Psychological Disorders, Cancer, Organismal Injury and Abnormalities
4	Adipose Tissue Up	ACVR1C,AMPK,ANO3,BCR (complex),caspase,CD3,CERS6,C	23	12	Lipid Metabolism, Small Molecule Biochemistry, Hereditary Disorder
5	Adipose Tissue Up	ADRB1,ADRB3,adrenoceptor,Akt,AMOTL2,AXIN2,Camkk,CAP	18	10	Cardiac Arrythmia, Cardiovascular Disease, Organismal Injury and Abnormalities
6	Adipose Tissue Up	AURKA,ZKSCAN2	2	1	Cancer, Cell Cycle, Cell Death and Survival
7	Adipose Tissue Up	PPP1CA,TMEM132C	2	1	Cellular Compromise, Cell-To-Cell Signaling and Interaction, Nervous System Development and Function
8	Adipose Tissue Up	PPP1CC,VSTM4	2	1	Cell Cycle, Cellular Movement, Cancer
9	Adipose Tissue Up	MIR5690,miR-5690 (miRNAs w/seed CAGCUAC)	2	1	
10	Adipose Tissue Up	PARVG,TAL1,WFDC1,ZNF677	2	1	Cardiovascular System Development and Function, Cell Cycle, Cellular Development

ID	Analysis	Molecules in Network	Score	Focus M	Top Diseases and Functions
1	Adipose Tissue Down	ACOT7,AP4M1,APOBEC3G,APP,ARMC1,ASGR1,BLNK,CAMI	42	17	Cell-To-Cell Signaling and Interaction, Small Molecule Biochemistry, Cell Death and Survival
2	Adipose Tissue Down	22(R)-hydroxycholesterol,ADRB,ASGR1,ATG16L2,CES4A,CH	36	15	Endocrine System Development and Function, Organ Development, Molecular Transport
3	Adipose Tissue Down	mir-921,miR-921 (miRNAs w/seed UAGUGAG)	3	1	
4	Adipose Tissue Down	CYP26C,CYP26C1	3	1	Dermatological Diseases and Conditions, Developmental Disorder, Drug Metabolism
5	Adipose Tissue Down	CA13,Carbonic anhydrase	3	1	Ophthalmic Disease, Organismal Injury and Abnormalities, Developmental Disorder
6	Adipose Tissue Down	CETN1,FBXO16,SKP1	2	1	Hereditary Disorder, Ophthalmic Disease, Organismal Injury and Abnormalities

Supplementary Table 13: Relative leukocyte fractions evaluated by CIBERSORT analysis on the stroma RNAseq dataset.

Input Sample	B cells naive	B cells memory	Plasma cells	T cells CD8	T cells CD4 naive	T cells CD4 memory resting	CD4 memory activated	T cells follicular helper	T cells regulatory (Tregs)	T cells gamma delta	NK cells resting	NK cells activated	Monocytes	Macrophages M0	Macrophages M1	Macrophages M2	Dendritic cells resting	Dendritic cells activated	Mast cells resting	Mast cells activated	Eosinophils	Neutrophils
Susceptible 1	0.150	0	0.162	0.013	0	0.418	0	0	0	0	0.038	0	0.061	0.036	0.052	0	0.009	0	0.061	0	0	0
Susceptible 2	0.139	0	0.299	0	0.043	0.104	0	0.030	0	0	0.003	0.065	0.184	0.029	0.013	0	0.052	0	0.039	0	0	0
Susceptible 3	0.217	0	0.145	0	0	0.132	0	0.002	0.041	0	0	0.030	0.086	0	0.025	0.056	0.075	0	0.191	0	0	0
Susceptible 4	0.116	0	0.188	0.007	0.035	0.080	0	0	0	0	0.040	0.035	0.180	0.018	0.014	0.135	0.023	0	0.129	0	0	0
Susceptible 5	0.177	0	0.138	0.033	0	0.052	0	0	0	0	0	0.093	0.041	0.033	0.021	0.059	0.070	0	0.284	0	0	0
Susceptible 6	0.300	0	0.123	0.062	0.015	0.034	0	0.006	0	0	0.011	0.034	0.134	0	0.020	0.063	0.115	0	0.082	0	0	0
Susceptible 7	0.229	0	0.252	0	0.026	0.225	0	0	0	0	0.035	0.044	0.021	0	0	0.053	0.040	0	0.073	0	0	0
HC 1	0.219	0	0.164	0.010	0	0.106	0	0.008	0	0	0	0.043	0.132	0	0.033	0.095	0.087	0	0.102	0	0	0
HC 2	0.088	0	0.034	0.036	0.031	0.446	0	0	0	0	0	0.109	0.211	0	0	0.015	0	0.011	0.019	0	0	0
HC 3	0.205	0	0.161	0	0.083	0.145	0	0	0	0	0	0.054	0.083	0.002	0.003	0.013	0.137	0	0.113	0	0	0
HC 4	0.129	0	0.186	0.022	0	0.284	0	0	0.005	0	0.016	0.080	0.093	0.014	0.023	0.010	0.010	0	0.128	0	0	0
HC 5	0.081	0	0.071	0.012	0	0.229	0	0	0	0	0.017	0.025	0.247	0	0	0	0.042	0	0.092	0	0	0.184
HC 6	0.075	0	0.086	0.004	0	0.092	0	0.005	0.021	0.001	0	0.039	0.530	0	0.038	0	0.040	0	0.069	0	0	0
HC 7	0.220	0	0.290	0	0.038	0	0.029	0.074	0	0.009	0.019	0	0.034	0.043	0.123	0	0.044	0	0.070	0	0.007	0
HC 8	0.260	0	0.210	0	0	0.110	0	0.006	0	0	0	0.061	0.033	0	0	0.079	0.098	0	0.144	0	0	0
HC 9	0.211	0	0.129	0.011	0	0.137	0	0.032	0	0	0	0.050	0.131	0	0.013	0.098	0.088	0	0.101	0	0	0
HC 10	0.156	0	0.404	0	0.029	0.131	0	0	0	0	0.006	0.052	0.092	0	0	0.041	0	0	0.088	0	0	0
HC 11	0.032	0	0.370	0.002	0	0.257	0	0	0.067	0	0	0.038	0.029	0	0	0	0	0	0.206	0	0	0
HC 12	0.071	0	0.103	0.015	0	0.371	0	0	0.026	0	0.020	0	0.069	0.021	0.029	0.039	0.087	0	0.148	0	0	0
HC 13	0.272	0	0.245	0	0	0.141	0	0.011	0	0	0	0.083	0.019	0.005	0	0.028	0.077	0	0.118	0	0	0
HC 14	0.179	0	0.174	0.060	0.073	0.208	0	0	0	0	0.005	0.046	0.067	0.002	0.013	0.002	0.074	0	0.097	0	0	0
HC 15	0.221	0	0.302	0	0.008	0.149	0	0	0	0	0	0.080	0.045	0	0.015	0.016	0.112	0	0.052	0	0	0
HC 16	0.104	0	0.171	0	0	0.205	0	0	0.014	0	0	0.034	0.083	0.029	0.005	0.038	0.196	0	0.120	0	0	0
p value	0.341		0.873	0.514	0.966	0.478	0.521	0.698	0.766	0.484	**0.025**	0.622	0.734	0.157	0.852	0.201	0.559	0.521	0.498		0.521	0.521
fold change											3.54											

HC: Healthy Control; RMSE = root mean squared error

Supplementary Table 14: Co-expression analysis of the microdissected epithelium (Epi)-, Stroma- and adipose tissue (Fat)- related data in Susceptible (Susc) and Healthy (HC) samples.

**Stroma-HC**	**Fat-HC**	**statistic**	***p* value**	**FDR**	**beta**
KCNF1	GJB1	71.25	1.7E-12	0.00	1.03
KDELC2	ST6GALNAC2	-36.07	3.8E-10	0.03	-1.59
TRAPPC2B	LOC100506388	35.94	3.9E-10	0.03	0.80
GRN	PAPD5	-34.86	5.0E-10	0.03	-0.75
AGTRAP	EMBP1	34.68	5.2E-10	0.03	3.02
METTL21EP	PLAG1	34.40	5.6E-10	0.03	0.88
HIST1H3E	CASP8	34.04	6.1E-10	0.03	0.17
VWA8	RPGR	32.27	9.3E-10	0.04	2.07
FBXL8	LOC100506388	30.16	1.6E-09	0.05	0.75
STAP1	ELP5	-29.93	1.7E-09	0.05	-0.12
SNORA23	TMEM176A	-29.80	1.7E-09	0.05	-0.97
MIB1	SH3KBP1	-28.94	2.2E-09	0.06	-1.52
CENPN	LINC00968	28.47	2.5E-09	0.06	4.02
SNORD17	RDH16	27.42	3.4E-09	0.07	1.21
FBXO7	BEGAIN	-27.11	3.7E-09	0.07	-4.65
LOC101929066	KLRD1	26.88	3.9E-09	0.07	1.06
C21orf62-AS1	RORA-AS1	26.78	4.1E-09	0.07	2.03
FAM171A1	ARRDC2	-26.67	4.2E-09	0.07	-1.62
ZNF611	TMEM161A	26.46	4.5E-09	0.07	1.43
RFESD	FBXO48	26.22	4.8E-09	0.07	0.69
LOC101928979	LOC100506388	26.04	5.1E-09	0.07	0.99
RASA4	MIR616	25.77	5.5E-09	0.07	0.83
INADL	SLC37A2	-25.75	5.5E-09	0.07	-1.33
GPD2	MEPCE	25.66	5.7E-09	0.07	0.85
JADE1	ISLR	-25.63	5.8E-09	0.07	-1.93
FOXN3-AS1	LOC100506497	25.42	6.2E-09	0.07	1.20
MYOZ1	KIAA0196	25.26	6.5E-09	0.08	0.17
LRG1	SLC25A37	24.92	7.2E-09	0.08	0.57
AKAP9	CREBZF	24.83	7.4E-09	0.08	1.46
HIST1H2AI	KIFC1	24.52	8.2E-09	0.09	1.15

**Epi-HC**	**Stroma-HC**	**statistic**	***p* value**	**FDR**	**beta**
COL5A2	COL3A1	13.31	1.0E-09	0.07	1.31
WISP2	WISP2	13.22	1.1E-09	0.07	0.64
TNFSF4	LOC441455	13.15	1.2E-09	0.07	1.87
LAMTOR2	FOXF1	13.10	1.3E-09	0.07	3.45
GSTT1	LOC391322	13.05	1.4E-09	0.07	0.83
HMCN1	COL3A1	12.90	1.6E-09	0.07	2.19
MIR26A2	SHROOM1	12.78	1.8E-09	0.07	0.70
SCN3B	BATF3	12.77	1.8E-09	0.07	0.98
DCST1	ZNF663P	12.65	2.1E-09	0.07	1.29
PTTG2	PILRB	12.21	3.4E-09	0.10	0.72
COL6A3	SPARC	11.96	4.5E-09	0.10	0.73
JADE1	PXYLP1	-11.94	4.6E-09	0.10	-3.75
ARRDC5	C16orf87	11.93	4.7E-09	0.10	0.39
PLIN4	PTPN2	-11.80	5.4E-09	0.10	-0.21
SNORD116-18	SNORD116-18	11.77	5.6E-09	0.10	0.84
SPATA5L1	PMS2P5	11.70	6.1E-09	0.10	1.71
CYP4F24P	CYP4F24P	11.66	6.4E-09	0.10	1.13
DLSTP1	LOC102724190	11.61	6.8E-09	0.10	0.86
RABL2B	PNPLA6	11.59	6.9E-09	0.10	0.63
LINC00906	MIR601	-11.57	7.1E-09	0.10	-3.27
RASSF2	RNVU1-8	-11.52	7.6E-09	0.10	-2.18
SLC28A1	NHP2L1	11.50	7.7E-09	0.10	0.09
LINC00176	QKI	-11.49	7.8E-09	0.10	-0.12
JADE1	ADAMTS8	-11.42	8.5E-09	0.10	-5.92
RPGR	ATF6B	11.38	8.9E-09	0.10	0.63

**Epi-HC**	**Fat-HC**	**statistic**	***p* value**	**FDR**	**beta**
ALDH1L1-AS2	MEAT6	Inf	0.0E+00	0.00	1.83
ADGRG4	UGT2B15	Inf	0.0E+00	0.00	1.87
ALDH1L1-AS2	UGT2B11	Inf	0.0E+00	0.00	3.20
LINC01028	MEAT6	Inf	0.0E+00	0.00	1.83
LINC01028	UGT2B11	Inf	0.0E+00	0.00	3.20
MIR3612	C1QL2	Inf	0.0E+00	0.00	1.35
MIR3612	FLJ31662	Inf	0.0E+00	0.00	1.03
ADGRG4	CRISP3	189812531.25	6.6E-64	0.00	2.19
LOC101928223	MEAT6	189812531.25	6.6E-64	0.00	0.82
LOC101928223	UGT2B11	134217728.00	1.1E-62	0.00	1.44
PGAP1	ZFPM2-AS1	34.94	4.9E-10	0.01	2.77
NOMO1	RFC1	-32.76	8.2E-10	0.02	-0.65
NRXN3	FRMD3	-29.79	1.7E-09	0.04	-1.32
DERL2	NIPSNAP3B	29.74	1.8E-09	0.04	4.62
HM13	RAB40B	28.62	2.4E-09	0.05	1.13
RPL41	AGTR1	26.46	4.5E-09	0.09	1.95
THAP7-AS1	FOXO1	26.13	4.9E-09	0.09	0.79
SLC2A8	YOD1	25.93	5.2E-09	0.09	0.95
GTF2H2C	BCDIN3D-AS1	24.94	7.1E-09	0.12	1.33
RERE	FNTA	-24.23	9.0E-09	0.14	-1.51
CDH1	PYGL	-23.93	9.9E-09	0.15	-1.51

**Stroma-Sus**	**Fat-Sus**	**statistic**	***p* value**	**FDR**	**beta**
snps	gene	statistic	pvalue	FDR	beta
SSRP1	HIST2H3D	1689.49	4.6E-10	0.14	7.83
SLC1A2	KLHL20	-832.70	3.8E-09	0.57	-0.15
HS2ST1	ADCY8	698.79	6.5E-09	0.64	14.29

**Epi-Susc**	**Stroma-Susc**	**statistic**	***p* value**	**FDR**	**beta**
AKR1B1	COLGALT2	150.69	2.4E-10	0.07	3.50
MIR4523	AARD	-102.06	1.7E-09	0.25	-1.06
ZNF763	APPL1	-81.75	5.2E-09	0.51	-0.37
HSD17B7P2	TRPV2	-72.65	9.4E-09	0.60	-0.48

**Epi-Susc**	**Fat-Susc**	**statistic**	***p* value**	**FDR**	**beta**
LOC101926964	CA6	Inf	0.0E+00	0.00	1.43
LOC101926908	CYP24A1	Inf	0.0E+00	0.00	1.44
LOC101926964	CR2	Inf	0.0E+00	0.00	2.59
LOC101926964	FLJ31662	Inf	0.0E+00	0.00	1.43
LOC101926908	KIAA2012	Inf	0.0E+00	0.00	2.84
LOC101926964	LINC01122	Inf	0.0E+00	0.00	2.13
LOC101926964	LINC01132	Inf	0.0E+00	0.00	3.06
LOC101926964	NEBL-AS1	Inf	0.0E+00	0.00	1.43
LOC101926908	TCN1	Inf	0.0E+00	0.00	1.44
LOC101926964	SRMS	Inf	0.0E+00	0.00	3.21
ALDH1L1-AS2	IL22RA1	116235962.09	1.4E-24	0.00	0.66
ALDH1L1-AS2	NUPR1L	116235962.09	1.4E-24	0.00	0.49
LOC101926964	C2CD4D	116235962.09	1.4E-24	0.00	2.32
LOC101926908	CTAGE15	116235962.09	1.4E-24	0.00	1.92
LOC101926964	CEMP1	116235962.09	1.4E-24	0.00	2.32
LOC101926964	FDCSP	116235962.09	1.4E-24	0.00	4.07
LOC101926908	KRT86	116235962.09	1.4E-24	0.00	1.92
LOC101926964	LIPG	116235962.09	1.4E-24	0.00	2.32
LOC101926964	MEAT6	116235962.09	1.4E-24	0.00	3.87
LOC101926964	SIX3	116235962.09	1.4E-24	0.00	2.32
LOC101926908	TNFSF11	116235962.09	1.4E-24	0.00	1.92
LOC101926964	ZSCAN1	116235962.09	1.4E-24	0.00	2.47
LHCGR	SYCE3	1405.28	7.9E-10	0.01	1.43
MAGT1	CYB5D2	-1064.12	1.8E-09	0.02	-0.75
TOB1	FBXO28	682.04	7.0E-09	0.08	0.79
