# Supplementary material for: Upregulation of lipid metabolism genes in the breast prior to cancer diagnosis
Source: NPJ Breast Cancer. 2020 Oct 6;6:50. doi: 10.1038/s41523-020-00191-8 (PMC7538898; doi:10.1038/s41523-020-00191-8)
Supplement: Supplementary file 2 — Reporting Summary [file 41523_2020_191_MOESM2_ESM.pdf]

nature research

Corresponding author(s): Natascia Marino
Last updated by author(s): 2020/05/30

Reporting Summary

Nature Research wishes to improve the reproducibility of the work that we publish. This form provides structure for consistency and transparency in reporting. For further information on Nature Research policies, see our Editorial Policies and the Editorial Policy Checklist.

Statistics

For all statistical analyses, confirm that the following items are present in the figure legend, table legend, main text, or Methods section.

n/a	Confirmed	
☐	☒	The exact sample size (*n*) for each experimental group/condition, given as a discrete number and unit of measurement
☐	☒	A statement on whether measurements were taken from distinct samples or whether the same sample was measured repeatedly
☐	☒	The statistical test(s) used AND whether they are one- or two-sided<br>*Only common tests should be described solely by name; describe more complex techniques in the Methods section.*
☒	☐	A description of all covariates tested
☐	☒	A description of any assumptions or corrections, such as tests of normality and adjustment for multiple comparisons
☐	☒	A full description of the statistical parameters including central tendency (e.g. means) or other basic estimates (e.g. regression coefficient) AND variation (e.g. standard deviation) or associated estimates of uncertainty (e.g. confidence intervals)
☐	☒	For null hypothesis testing, the test statistic (e.g. *F*, *t*, *r*) with confidence intervals, effect sizes, degrees of freedom and *P* value noted<br>*Give P values as exact values whenever suitable.*
☒	☐	For Bayesian analysis, information on the choice of priors and Markov chain Monte Carlo settings
☐	☒	For hierarchical and complex designs, identification of the appropriate level for tests and full reporting of outcomes
☐	☒	Estimates of effect sizes (e.g. Cohen's *d*, Pearson's *r*), indicating how they were calculated

Our web collection on statistics for biologists contains articles on many of the points above.

Software and code

Policy information about availability of computer code

Data collection	Transcriptomic data were generated with 2×75bp paired-end configuration on the HiSeq4000 (Illumina) using HiSeq 3000/4000 PE SBS Kit. Images of the hematoxylin and eosin staining as well as the immunostainings were obtained using the the Aperio ScanScope CS system (Aperio, Vista, CA).
Data analysis	The sequencing data were first assessed using FastQC (Babraham Bioinformatics, Cambridge, UK) for quality control. Transcriptome sequenThe data was normalized using TMM (trimmed mean of M values) method. Differential expression analysis was performed using EdgeR cing recovered ~36-40 million raw reads from each of the 23 breast specimens. After normalization, ~23-30 million reads were uniquely mapped using STAR v2.5 and UCSC hg19 as reference genome 52 and ~9-14.6 million reads were assigned to annotated genes. Immunostaining was quantified using Aperio Image Scope v12.3.2 and QuPath v0.2.0 softwares.

For manuscripts utilizing custom algorithms or software that are central to the research but not yet described in published literature, software must be made available to editors and reviewers. We strongly encourage code deposition in a community repository (e.g. GitHub). See the Nature Research guidelines for submitting code & software for further information.

Data

Policy information about availability of data

All manuscripts must include a data availability statement. This statement should provide the following information, where applicable:

- Accession codes, unique identifiers, or web links for publicly available datasets
- A list of figures that have associated raw data
- A description of any restrictions on data availability

The datasets supporting the conclusions of this article are available in the GEO repository (GSE141828, https://www.ncbi.nlm.nih.gov/geo/query/acc.cgi?acc=GSE141828).

Field-specific reporting

Please select the one below that is the best fit for your research. If you are not sure, read the appropriate sections before making your selection.

☒ Life sciences ☐ Behavioural & social sciences ☐ Ecological, evolutionary & environmental sciences

For a reference copy of the document with all sections, see nature.com/documents/nr-reporting-summary-flat.pdf

Life sciences study design

All studies must disclose on these points even when the disclosure is negative.

Sample size	the transcriptomic analysis of the microdissected breast epithelium, stroma and adipose tissue was performed on the breast tissue cores donated by 23 women (7 susceptible and 16 healthy control). Immunohistochemistry experiments were performed on breast tissue sections from additional 62 women.
Data exclusions	the microdissected epithelium, stroma and adipose tissue from two breasts donated by healthy women resulted to be outliers (from PCA analysis) and were removed from the study.
Replication	all the data were obtained from multiple samples i.e.microdissected breast epithelial compartments from 7 susceptible women were compared with breast from different subjects whithin the same group.
Randomization	Randomization of the transcriptomic data was not requested. Susceptible donors were identified through an annual medical follow-up as individuals who had a breast cancer diagnosis post-tissue donation and lacking of any mutation in known breast cancer predisposition genes upon genetic testing. The subjects in the susceptible normal and healthy controls cohorts were matched (at a ratio of either 1:2 or 1:3) according to age, racial background, body mass index and follow-up time, the latter defined as the interval from date of tissue donation to last medical follow-up.
Blinding	The persons performing microdissection and statistical analysis were unaware of the sample identity. (susceptible vs healthy control).

Reporting for specific materials, systems and methods

We require information from authors about some types of materials, experimental systems and methods used in many studies. Here, indicate whether each material, system or method listed is relevant to your study. If you are not sure if a list item applies to your research, read the appropriate section before selecting a response.

Materials & experimental systems

n/a	Involved in the study	
☐	☒	Antibodies
☐	☒	Eukaryotic cell lines
☒	☐	Palaeontology and archaeology
☒	☐	Animals and other organisms
☒	☐	Human research participants
☒	☐	Clinical data
☒	☐	Dual use research of concern

Methods

n/a	Involved in the study	
☒	☐	ChIP-seq
☒	☐	Flow cytometry
☒	☐	MRI-based neuroimaging

Antibodies

Antibodies used	1. AKR1C1 (GeneTex, GTX105620, 1:100)<br>2. HSL (ThermoFisher Scientific, PA5-2638, 1:250),<br>3. CD36 (Sigma, HPA002018, 1:500)<br>4. AQP7 (Novus, NBP1-30862, 1:1000).<br>5. CD45 (Dako, M0701, 1:100).<br>6. CD68 (Dako M0876, 1:50),<br>7. ki67 (Dako M7240, 1:50)<br>8. CD4 (Leica Biosystems, NCL-L-CD4-368, 1:50),<br>9. CD8 (Dako, M7103, 1:200).<br>10. CD20 (Dako, M0755, 1:200).<br>11. LC3B (GeneTex, GTX116080, 1:500).<br>12. TOMM20 (Abcam, ab56783, 1:150)
Validation	In order to find the best working conditions, the antibodies were tested first on breast tumor sections at different concentraztions and incubation times that were chosen accordingly to the manufacturer' datasheet.<br>1. https://www.genetex.com/Product/Detail/AKR1C1-antibody/GTX105620<br>2. https://www.thermofisher.com/antibody/product/HSL-Antibody-Polyclonal/PA5-26383

3. https://www.sigmaaldrich.com/catalog/product/sigma/hpa002018?lang=en&region=US
4. https://www.novusbio.com/products/aquaporin-7-antibody_nbp1-30862
5. https://www.agilent.com/en/product/immunohistochemistry/antibodies-controls/primary-antibodies/cd45-leucocyte-common-antigen-(concentrate)-76507
6. https://www.agilent.com/en/product/immunohistochemistry/antibodies-controls/primary-antibodies/cd68-(concentrate)-76550
7. https://www.agilent.com/store/en_US/LCat-SubCat3ECS_86510/Ki-67-Antigen-MIB-1-Concentrate-.
8.. https://shop.leicabiosystems.com/us/ihc-ish/ihc-primary-antibodies/pid-cd4
9. https://www.agilent.com/en/product/immunohistochemistry/antibodies-controls/primary-antibodies/cd8-(concentrate)-76631
10. https://www.agilent.com/en/product/immunohistochemistry/antibodies-controls/primary-antibodies/cd20cy-(concentrate)-76520
11. https://www.genetex.com/Product/Detail/LC3B-antibody-N1C3/GTX116080
12. https://www.abcam.com/tomm20-antibody-mitochondrial-marker-ab56783.html

Eukaryotic cell lines

Policy information about cell lines

Cell line source(s)	In this study we used primary breast epithelial cells (and primary fibroblasts as staining control)
Authentication	The cells have not been deeply authenticated. As shown in supplementary figure S5D, staining with Ecadherin, epithelial marker, and vimentin, mesenchimal marker, is used to define the epithelial lineage of the primary culture (human primary fibroblasts are shown as control for vimentin).
Mycoplasma contamination	Cells were grown without penicillin/ streptomicin. PCR-based mycoplasma test (https://www.thermofisher.com/order/catalog/product/4460623?SID=srch-srp-4460623#/4460623?SID=srch-srp-4460623)
Commonly misidentified lines (See ICLAC register)	No commonly misidentified cell lines were used.
